# Supplementary material for: NimA promotes cell adhesion at the blood brain barrier of the Drosophila nervous system
Source: EMBO Rep. 2026 Mar 3;27(7):1648–65. doi: 10.1038/s44319-026-00728-1 (PMC13076899; doi:10.1038/s44319-026-00728-1)
Supplement: Supplementary file 1 — Appendix [file 44319_2026_728_MOESM1_ESM.pdf]

Appendix

NimA promotes cell adhesion at the blood brain barrier of the *Drosophila* nervous system

List:

- Appendix_Supplementary_Methods_CRISPR_Design_Report_NimA-Gal4

CRISPR Design Report

**Report No.:**	RWG9275	**Date:**	2021.05.20
**Reporter:**	Dr. Pei-Tseng Lee	**E-mail:**	peitseng.lee@wellgenetics.com

****

Gene: *NimA/CG42282* **Case No.:** 210198

Project: making a null allele of *NimA* by deleting entire CDS and knocking in Gal4 and a floxed selection marker to facilitate genetic screening

Method: CRISPR/Cas9-mediated genome editing by homology-dependent repair (HDR) using two guide RNA(s) and a dsDNA plasmid donor

Gene and Method

Gene: *NimA/CG42282*

Location: 2L (34E5-34E5)

Method: CRISPR/Cas9-mediated genome editing by homology-dependent repair (HDR) using two guide RNAs and a dsDNA plasmid donor

Knock-in cassette: Gal4-3xP3-RFP

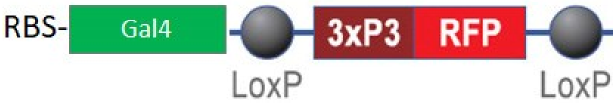

Injection strain: *w[1118]*

Genome Editing:

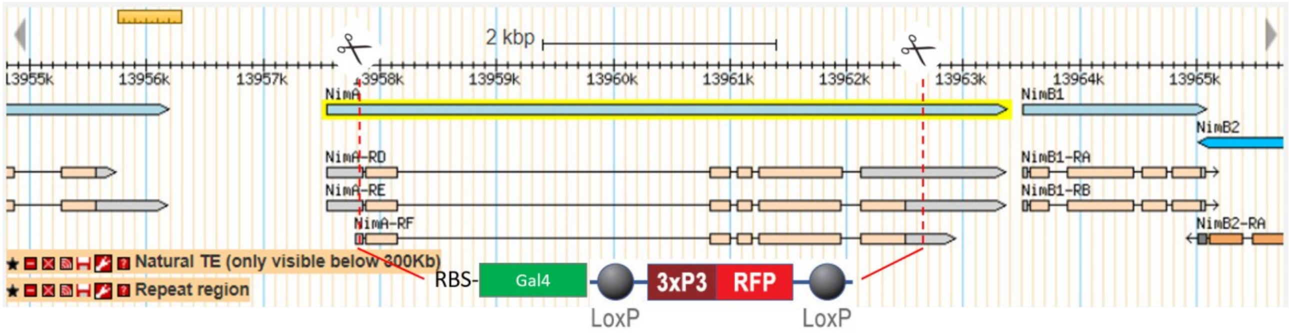

(1) The entire CDS region of *NimA* will be deleted and replaced by cassette Gal4-3xP3-RFP.

(2) Cassette Gal4-3xP3-RFP contains ribosome binding sequence (RBS), Gal4, SV40 polyA terminator, and floxed 3xP3-RFP as the selection marker.

(3) The selection marker 3xP3-RFP contains loxP site, the artificial 3xP3 promoter (three tandem copies of the Pax-6 homodimer binding site and TATA-homology of *hsp70*), RFP, alpha-Tubulin 3’UTR and loxP site. It facilitates the genetic screening and can be flipped out by Cre recombinase.

☎ +8862-2651-1809 ✉ info@wellgenetics.com 2F.-2, No.32, Sec. 1, Chenggong Rd., Taipei City 11570, Taiwan (R.O.C.)
wellgenetics.com

CRISPR Target Sites

Guide RNA Quality Instruction

Guide RNA quality is shown in color dots for your references.

	Distance	Off target	GC Content	T #
Strong site	<50bp	0	45%-70%	≦1
Weak site	50-100bp	Potential off targets on untargeted chromosomes	40%, 75%	2
Bad site	>100bp	Potential off targets on targeted chromosomes	< 40%, >75%	≧3

1. The distance from knockin site or deletion breakpoint to cutting site of Cas9.
2. Predicted off targets on other chromosomes are indicated.
3. GC content 45% to 70% of target sequence provides the best efficiency.
4. High thymine numbers at 17th to 20th nucleotide of the guide RNA target site may decrease the efficiency.

gRNA 1 (upstream)

CRISPR Target Site [PAM]: ACTGCTCCTCCTGCTTGCAA[TGG]

CRISPR Target Strand in the Genome: plus

Cutting Site: +43 nt from ATG of *NimA*

Distance: +82 nt from upstream breakpoint to cutting site of Cas9

GC Content: 55%

T number at 17th to 20th Nucleotides: 0

Off target: 0

Guide RNA Primers:

Sense oligo 5’- CTTCGACTGCTCCTCCTGCTTGCAA

Antisense oligo 5’- AAACTTGCAAGCAGGAGGAGCAGTC

PAM mutation: not required

Upstream Homology Arm: 997bp, the -1,036 nt to -40 nt from ATG of *NimA*

Forward Oligo 5’- ATTTAACTGCCATGTCTTGCAC

Reverse Oligo 5’- GGCATTCACTTTATTCCTCGTT

Downstream Homology Arm: same as below

+8862-2651-1809 info@wellgenetics.com 2F.-2, No.32, Sec. 1, Chenggong Rd., Taipei City 11570, Taiwan (R.O.C.)
wellgenetics.com

WELLGENETICS

gRNA 2 (downstream)

CRISPR Target Site [PAM]: GTGCCCTTCTAACATATACC[AGG]

CRISPR Target Strand in the Genome: plus

Cutting Site: +124 nt from Stop Codon of *NimA-RE/F*

Distance: -56 nt from downstream breakpoint to cutting site of Cas9

GC Content: 45%

T number at 17th to 20th Nucleotides: 1

Off target: 0

Guide RNA Primers:

Sense oligo 5’- CTTCGTGCCCTTCTAACATATACC

Antisense oligo 5’- AAACGGTATATGTTAGAAGGGCAC

PAM mutation: not required

Upstream Homology Arm: same as above

Downstream Homology Arm: 986bp, the +181 nt to +1,166 nt from Stop Codon of *NimA-RE/F*

Forward Oligo: 5’- AGTCTGCGAGTTTTTACAACCA

Reverse Oligo: 5’- AATCGCCGTACTCCGTATTC

Donor Design

Annotation

NNNNN: Homology Arm / Coding Region

NNNNN: Homology Arm / UTR

nnnnn: Homology Arm/ Intron

nnnnn: Homology Arm/ Intergenic Region

nnnnn: gRNA target sequence

nnnnnn: Cloning Site

nnnnn: LoxP

nnnnn: 3xP3 Promoter

NNNNN: RFP

nnnnn: alpha Tubulin 3'UTR

Sequence

atttaactgccatgtcttgcacttaagtattaattacgtttatgtttatcgtagatttaatctcactcttaagttaaacgcattttaatcttggcactttt
aatcaagcaacatattttcttagattgaaaagcaatataatatactaatttatcgttgattaaatttgatttagaacatatcttgcaaatctcagttt
tgccacctaatacccagttgtggatcatcgcttaattgggtctgaagaaaagcgcaagtcgatttgaaatgaaatcaaaacaaaagtgactac
gaaaacaactggcgtagtcgcgtttttgcaccgcaatgacagccgcatgttaaggacatgatcaacatttcaattagatgcaatcatcgccctcg
ccgcaatcgcggggctagtgggtatggggtgtaatcgctttggggagttgagtttcattcacatgcagtggagacttcgctgctcaccggcatgc
tttacatttcactttcgctttgtagaattaccatgaattgtagaattgggtgtgcagtgcgtacttccgctctccgatctcaagtccctcgccacccc
ggacggcctttattggctcgacgacaatttgcaatgactactgtattttgttgtagctttccgtagtttactttatttcgtgaaggaacattcggagt
gagtaacagaatcgccgccaacggggaaacagtttttacgagactcgCGATCCAGACGTACACGCAGCGCACATCAAACGGT
TTAAATTGATGGATGTAAGCGGATAAGATCCTCGTTTGGACCAGAACTAGATCAGAATATTAAATATTGTAGGA
ATGTGTTATGCGAATTAATAGTTCAAGGTCCTAAAAATAATAAACGGAGATGGGCAAAAGTGATTAAGGTTTTC
GGGGTATCAAAAGAATTTTCATCAATTAAAAAAAATTTGCGATCAGTAATCATTGATGGATATGGTAAACGAGG
AATAAAGTGAATGCC

agatctataacttcgtataatgtatgctatacgaagttat**ggtacc**ggatctaattcaattagagactaattcaattagagctaattcaattagga
tccaagcttatcgatttcgaaccctcgaccgccggagtataaatagaggcgcttcgtctacggagcgacaattcaattcaaacaagcaaagtga
acacgtcgctaagcgaaagctaagcaaataaacaagcgcagctgaacaagctaaacaatcgggcggccgcactagagccggtcgccaccA
TGAGGTCTTCCAAGAATGTTATCAAGGAGTTCATGAGGTTTAAGGTTCGCATGGAAGGAACGGTCAATGGGC
ACGAGTTTGAAATAGAAGGCGAAGGAGAGGGGAGGCCATACGAAGGCCACAATACCGTAAAGCTTAAGGTA
ACCAAGGGGGGACCTTTGCCATTTGCTTGGGATATTTTGTCACCACAATTTCAGTATGGAAGCAAGGTATATGT
CAAGCACCCTGCCGACATACCAGACTATAAAAAGCTGTCATTTCCTGAAGGATTTAAATGGGAAAGGGTCATG
AACTTTGAAGACGGTGGCGTCGTTACTGTAACCCAGGATTCCAGTTTGCAGGATGGCTGTTTCATCTACAAGG

TCAAGTTCATTGGCGTGAACTTTCCTTCCGATGGACCTGTTATGCAAAAGAAGACAATGGGCTGGGAAGCCA
GCACTGAGCGTTTGTATCCTCGTGATGGCGTGTTGAAAGGAGAGATTCATAAGGCTCTGAAGCTGAAAGACG
GTGGTCATTACCTAGTTGAATTCAAAAGTATTTACATGGCAAAGAAGCCTGTGCAGCTACCAGGGTACTACTAT
GTTGACTCCAAACTGGATATAACAAGCCACAACGAAGACTATACAATCGTTGAGCAGTATGAAAGAACCGAG
GGACGCCACCATCTGTTCCTTTAGcggccatcgaattcgagctcgcccactaagcgtcgcgccacttcaacgctcgatgggagcgtc
attggtgggcggggtaaccgtcgaaatcagtgtttacgcttccaatcgcaacaaaaaattcactgcaacactgaaaagcatacgaaaacgatg
aagattgtacgagaaaccataaagtattttatccacaaagacacgtatagcagaaaagccaagttaactcggcgataagttgtgtacacaaga
ataaaatcggccagattcagtgttgtcagaaataagaaaaccccactatgtttttctttgccttttctttctcccagcgatcattcatttcgtggtga
aagaacggggtcattgcacggagtttcgactgcgggaaagcagagctgccgttcacttcgtctataattagcgctttctattttccccgattcggg
ccgctgctgcgcttttccgcctgctgtttgtggcaagtgtagcagcaggctgtgcacgcagtgtggcatgcacttggctttccaccgttggtatcg
attctctgggacgatgagtcattcctttcggggccacagcataatcgttgccagctcaccgaaatggtgacttcatttcttaactgccgtcaagca
tgcgattgtacatacatacatatttatatatgtacatatttatgtgactatggtaggtcgatataatagcaatcaacgcaagcaaatgtgtcagtcc
tgcttacaggaacgattctatttagtaattttcgttgtataaagtaattatgtatgtatgtaagccccataaatctgaaacaattaggcaaaaccat
gcgaagct**ctgcag**ataacttcgtataatgtatgctatacgaagttat**gctagc**
AGTCTGCGAGTTTTTACAACCACACTTATTTTTATAACATACTTTAGATCAGTAATAAATTTATGTACAGAAGCCA
AATGTTAAGTGAGCATACATTTTGATTACATCATTATTTTAAGCTGCCCACGAATTATTATAACATTATATTATGGA
AGATATGATATGAACTGGGAAAAATAACATTAATTAAACATAAAAAAAAAAATTAATTCATAAATCATATAAGAA
TTGGATTGGCGATTATCAAACTAATAGAGTTTCACCTAATTTCTATTACTTTTTAAAGGTGATTTGCATGGTTAAA
GGTTTCTGCATTATTTAAGGGTAGTCTTTGATTAGATTTAACACTTTTATATCCATAAAAACCTATGTGTTAGACT
TTAAGTTTCTTTAAAGTCTACGATTGTGGCGTGTGTAGTTTCCGCATAGTTGTAACGTTTTAAATTTTTAAAAGT
ATGTTTGCTGTAGCAACAGTTTTAAAAATAAAAAATGTTATGACTTCAAAAAATATATAAAACTAATTATATTTAT
AGTTAAAAATAAAAAACAGCTATAATAACATTACTATATTATAAAACATATTATATTTATGCAAAAGCTGATTCATA
AAATAATTATTAGAATAAAGCCATGTAGCCACATTTTGTAAGTTAAAGACTATTATTTAATAAAATCGTCTAATAA
AACaatatgtgctggattgaaaagggcaacttaatgttttcatttttctctgaatgttttcgtttcgagactgagagttgatgctgataaggtctttt
gagtagattcaaaccccctggactgcctgttCATTCGCTACTCGACATCGAAGCTCAGAACACCAAGACGGGATGCACTGT
AGTGGACCGTTGGCCGGGCTTACCGCTCTACTGACGCTGGTGGCATTTCCTGTCCAGATTAAGACCGATTCCA
CGGTTTCCACGGAGCTCTACGGCGACATAGAGAATACGGAGTACGGCGATT
