# Supplementary figures and images for: NimA promotes cell adhesion at the blood brain barrier of the Drosophila nervous system
Source: EMBO Rep. 2026 Mar 3;27(7):1648–65. doi: 10.1038/s44319-026-00728-1 (PMC13076899; doi:10.1038/s44319-026-00728-1)

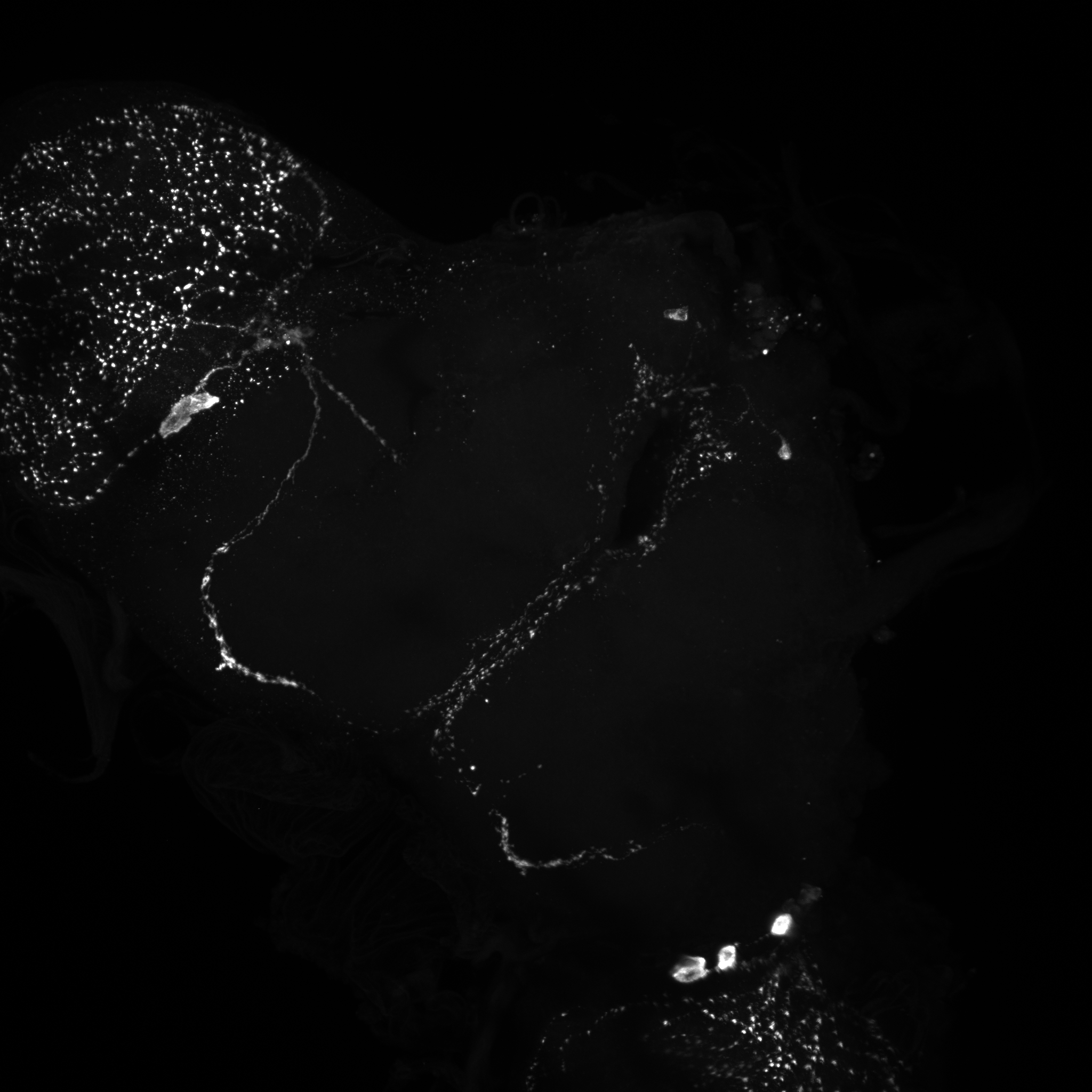

Supplement: Supplementary file 6 — Source data Fig. 2 [file 44319_2026_728_MOESM6_ESM.zip › Source_Data_Fig2/Fig2M-V/Source_Fig2P-R_Max_NimAhomo_0do.tif]

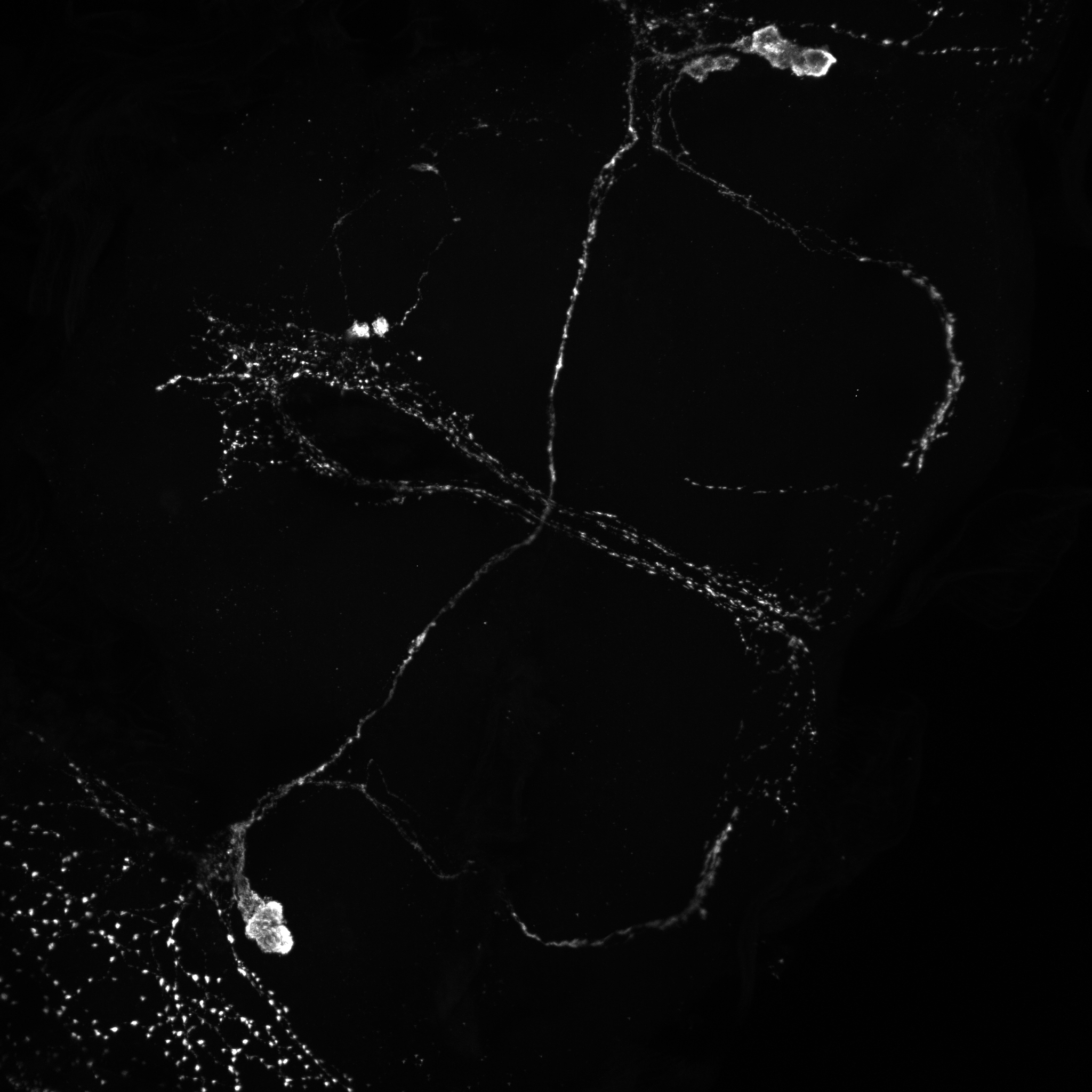

Supplement: Supplementary file 6 — Source data Fig. 2 [file 44319_2026_728_MOESM6_ESM.zip › Source_Data_Fig2/Fig2M-V/Source_Fig2M-O_Max_NimAhet_0do.tif]

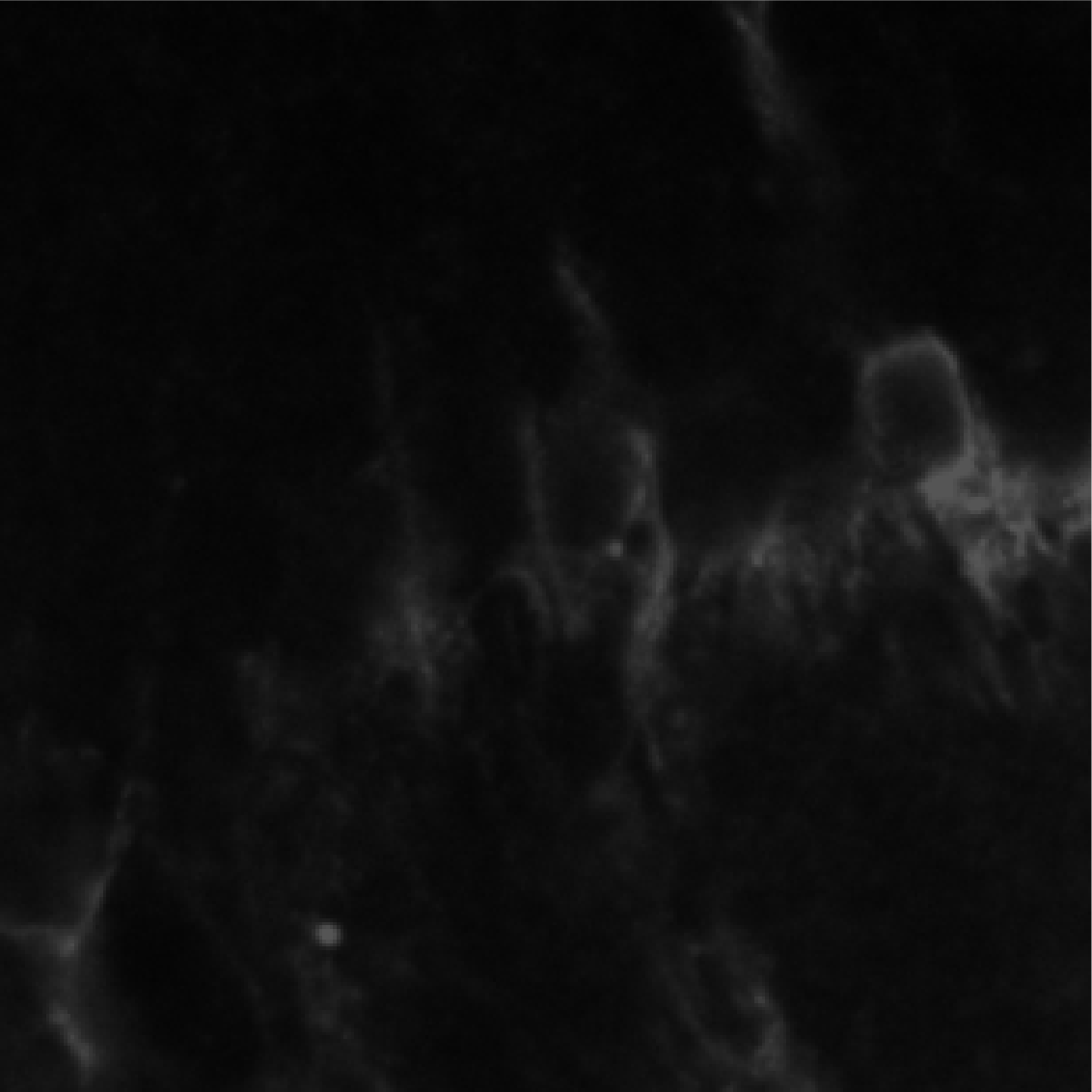

Supplement: Supplementary file 6 — Source data Fig. 2 [file 44319_2026_728_MOESM6_ESM.zip › Source_Data_Fig2/Fig2A-I/Source_Fig2H.tif]

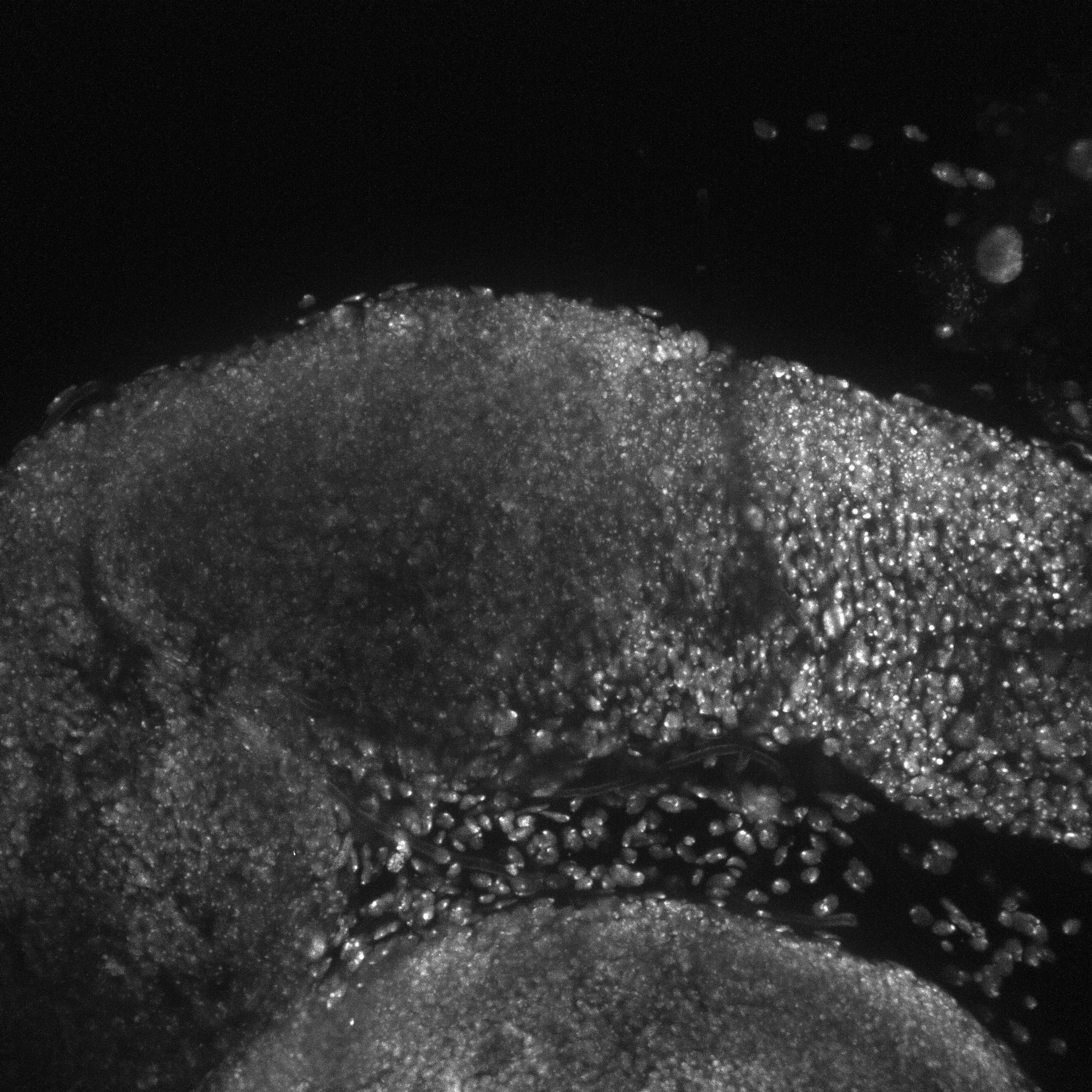

Supplement: Supplementary file 6 — Source data Fig. 2 [file 44319_2026_728_MOESM6_ESM.zip › Source_Data_Fig2/Fig2A-I/Source_Fig2F.tif]

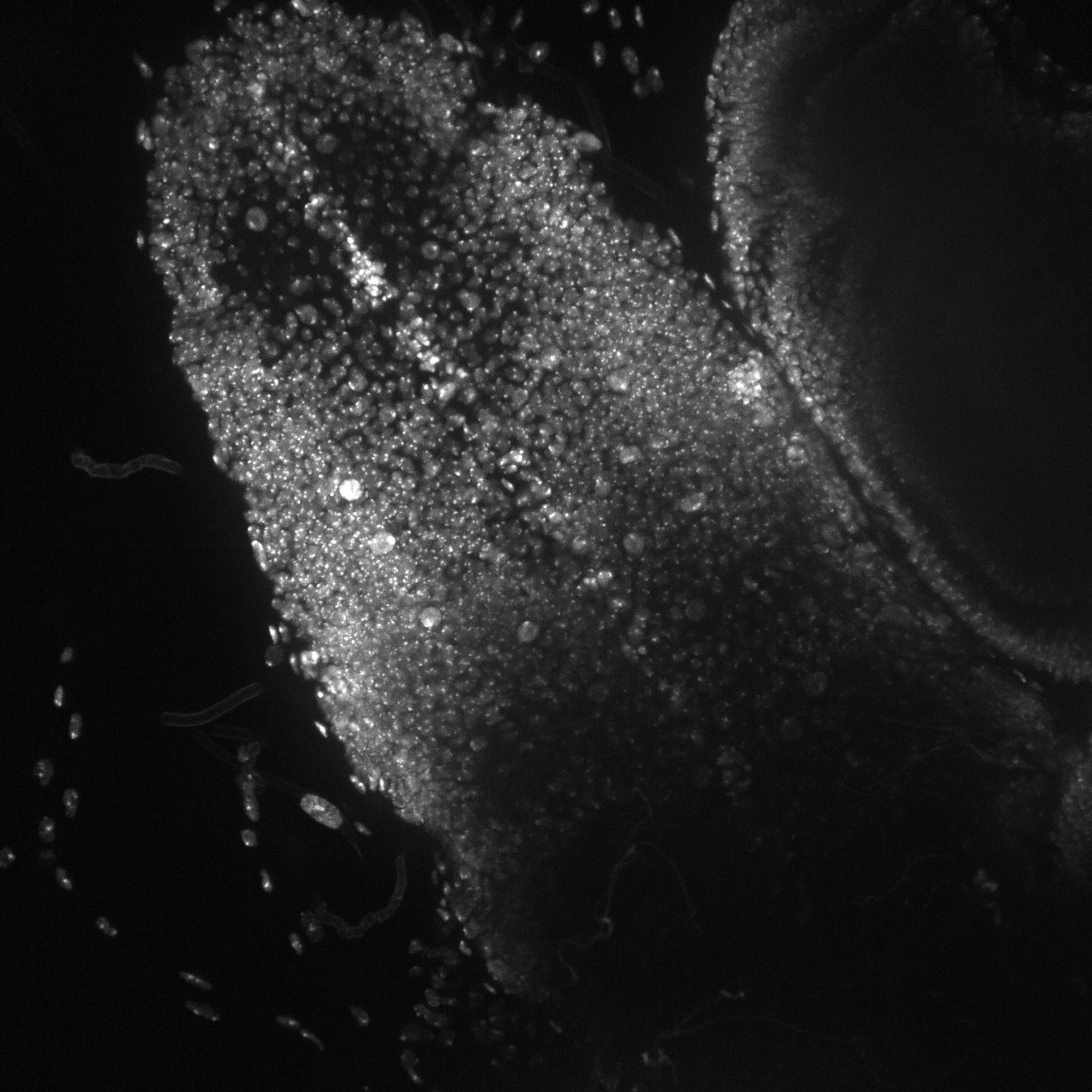

Supplement: Supplementary file 6 — Source data Fig. 2 [file 44319_2026_728_MOESM6_ESM.zip › Source_Data_Fig2/Fig2A-I/Source_Fig2E.tif]

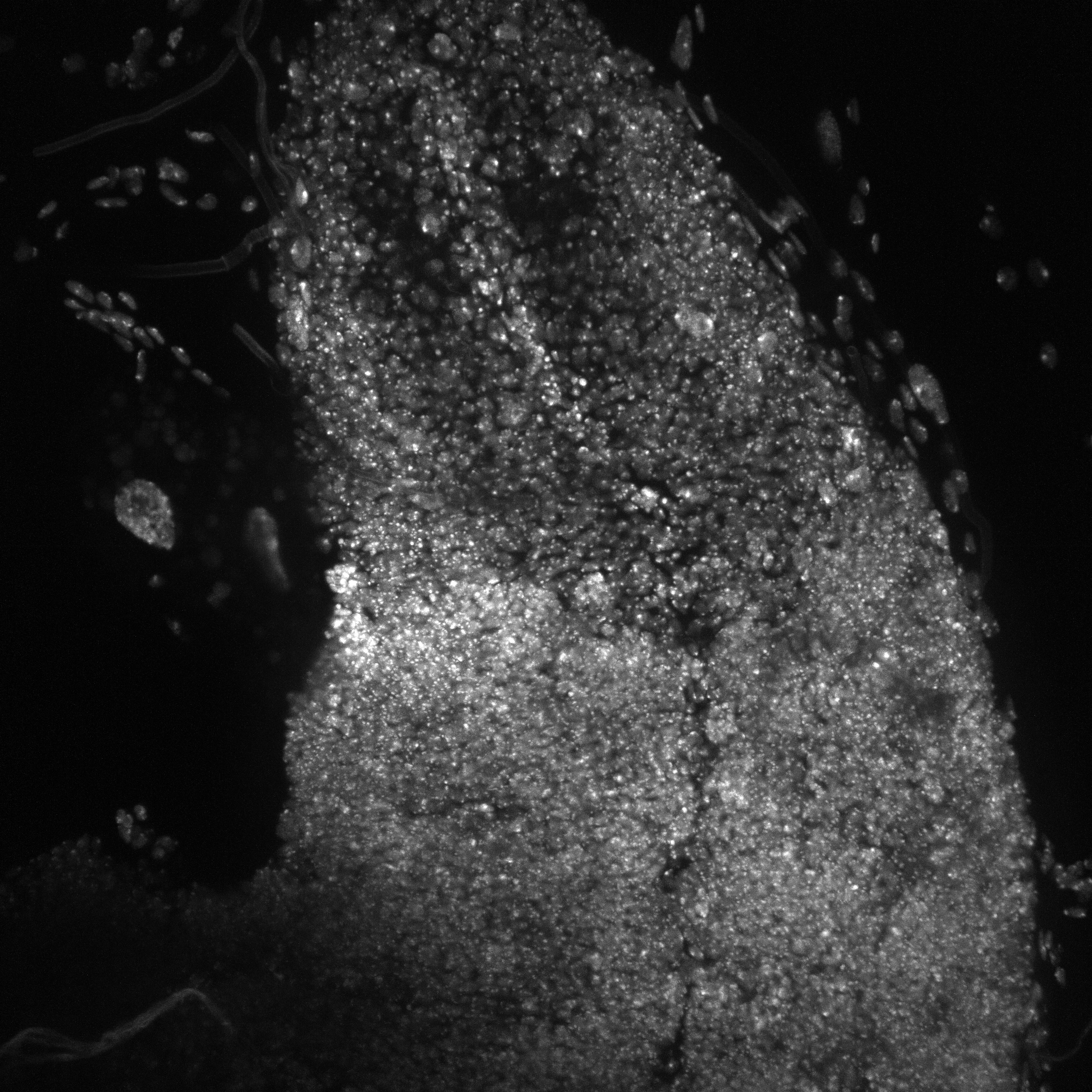

Supplement: Supplementary file 6 — Source data Fig. 2 [file 44319_2026_728_MOESM6_ESM.zip › Source_Data_Fig2/Fig2A-I/Source_Fig2D.tif]

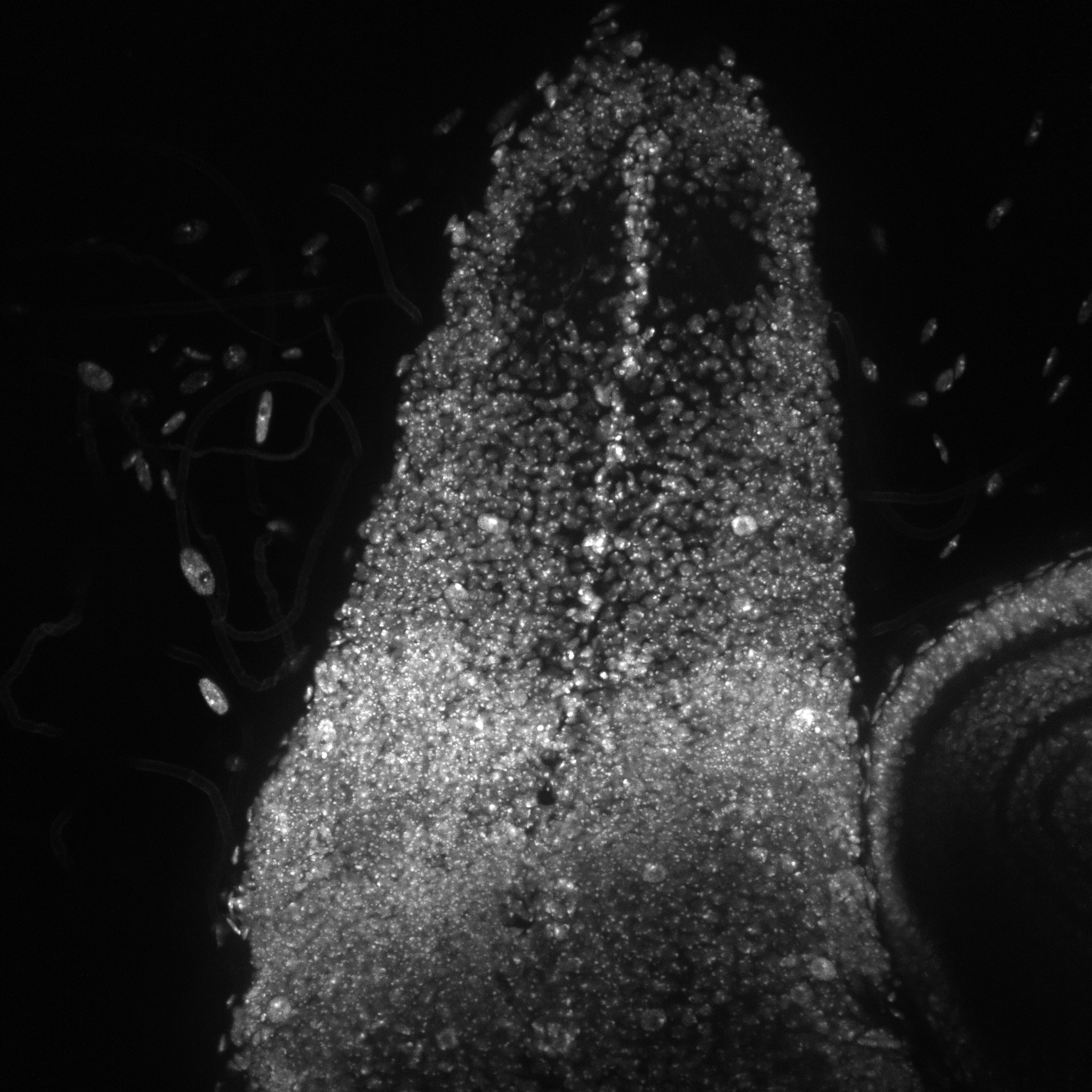

Supplement: Supplementary file 6 — Source data Fig. 2 [file 44319_2026_728_MOESM6_ESM.zip › Source_Data_Fig2/Fig2A-I/Source_Fig2C.tif]

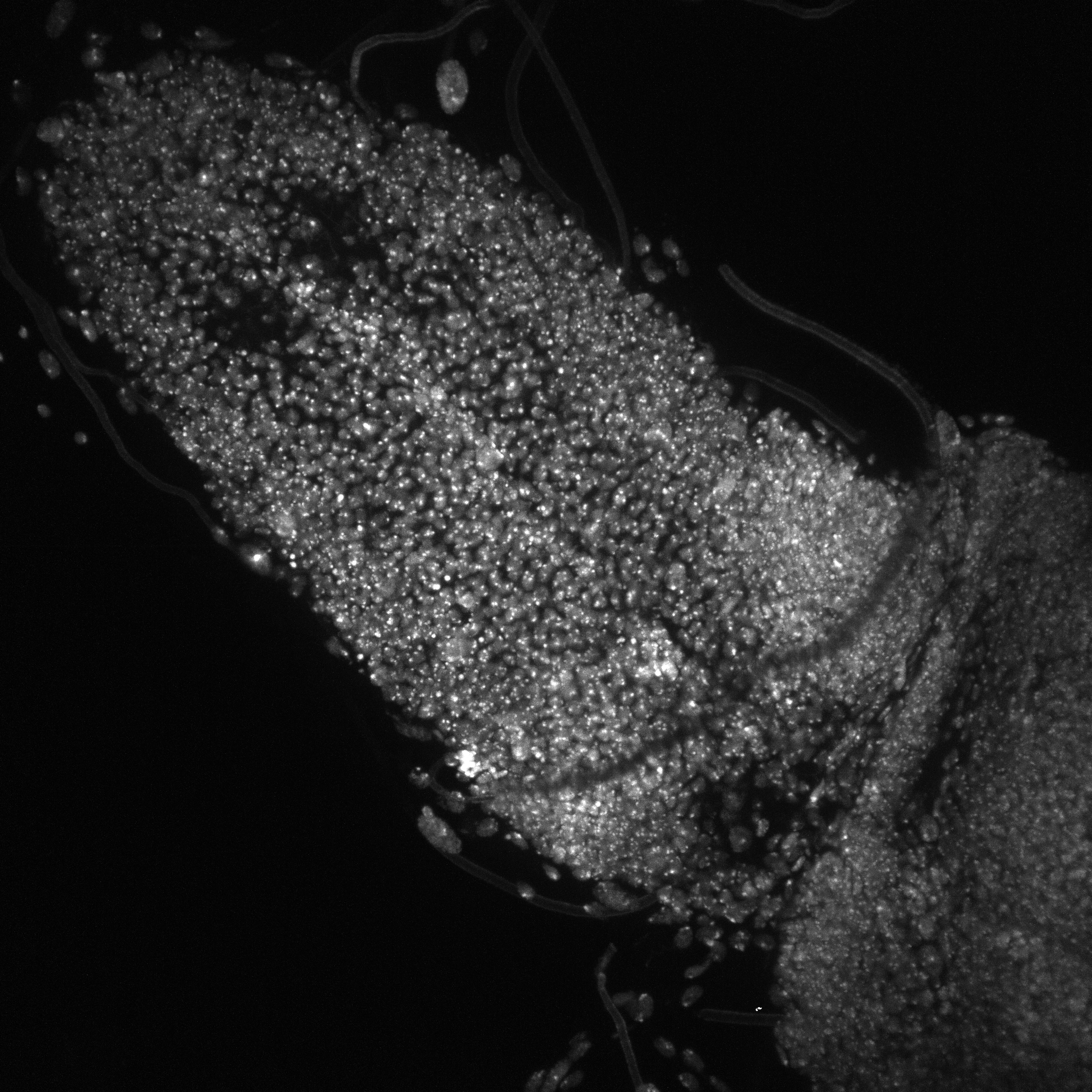

Supplement: Supplementary file 6 — Source data Fig. 2 [file 44319_2026_728_MOESM6_ESM.zip › Source_Data_Fig2/Fig2A-I/Source_Fig2B.tif]

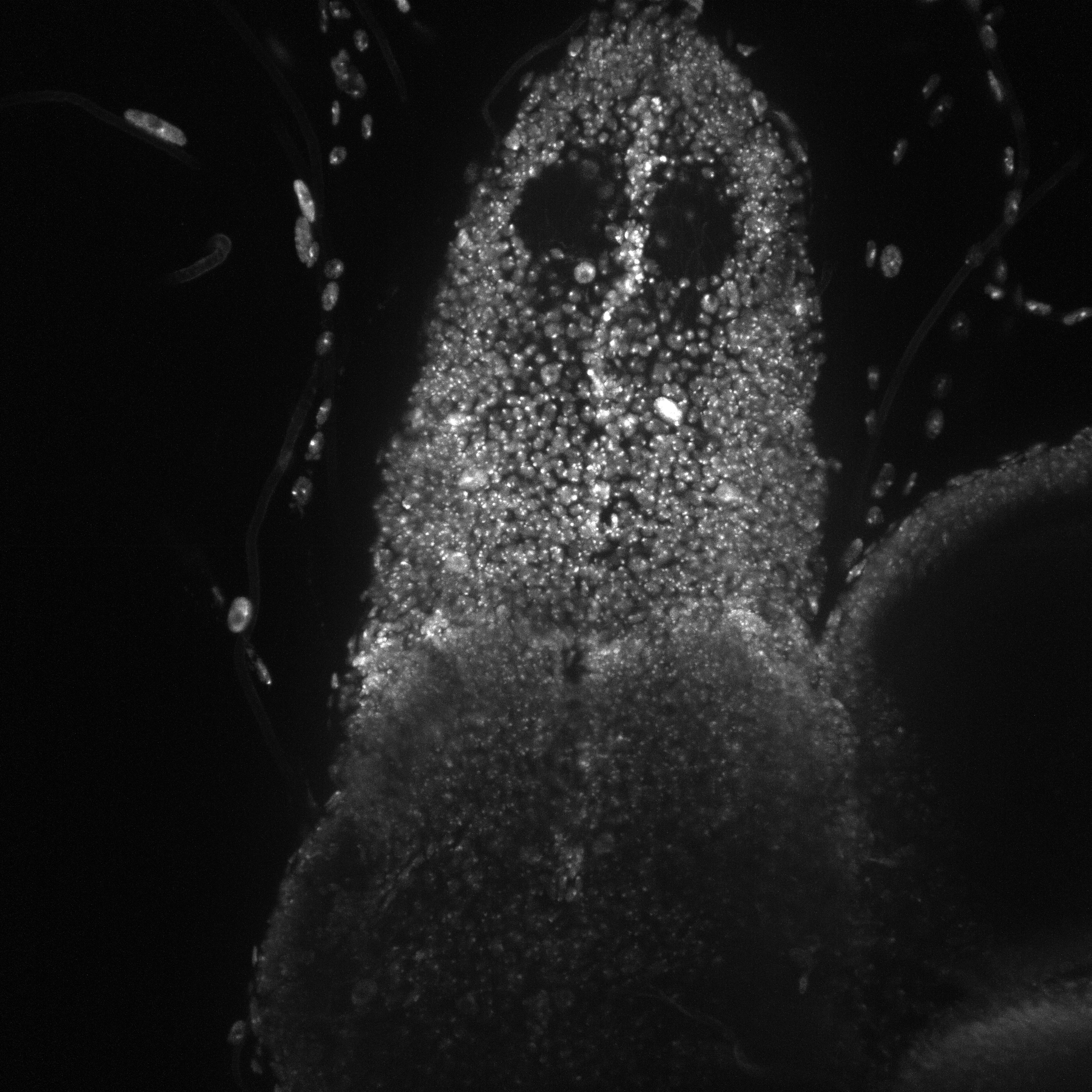

Supplement: Supplementary file 6 — Source data Fig. 2 [file 44319_2026_728_MOESM6_ESM.zip › Source_Data_Fig2/Fig2A-I/Source_Fig2A.tif]

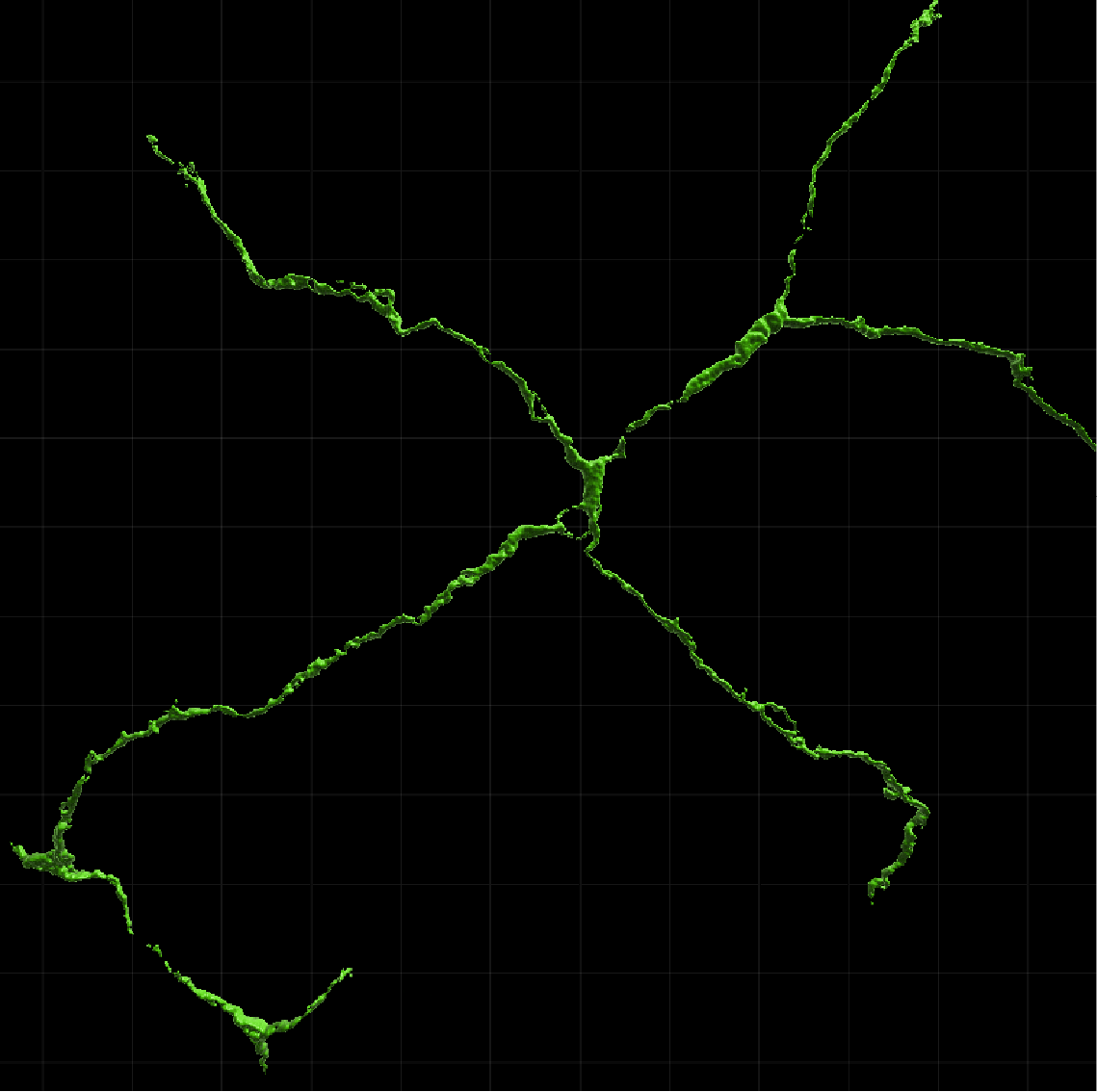

Supplement: Supplementary file 7 — Source data Fig. 3 [file 44319_2026_728_MOESM7_ESM.zip › Source_Data_Fig3/Fig3I-M/Source_Fig3K.tif]

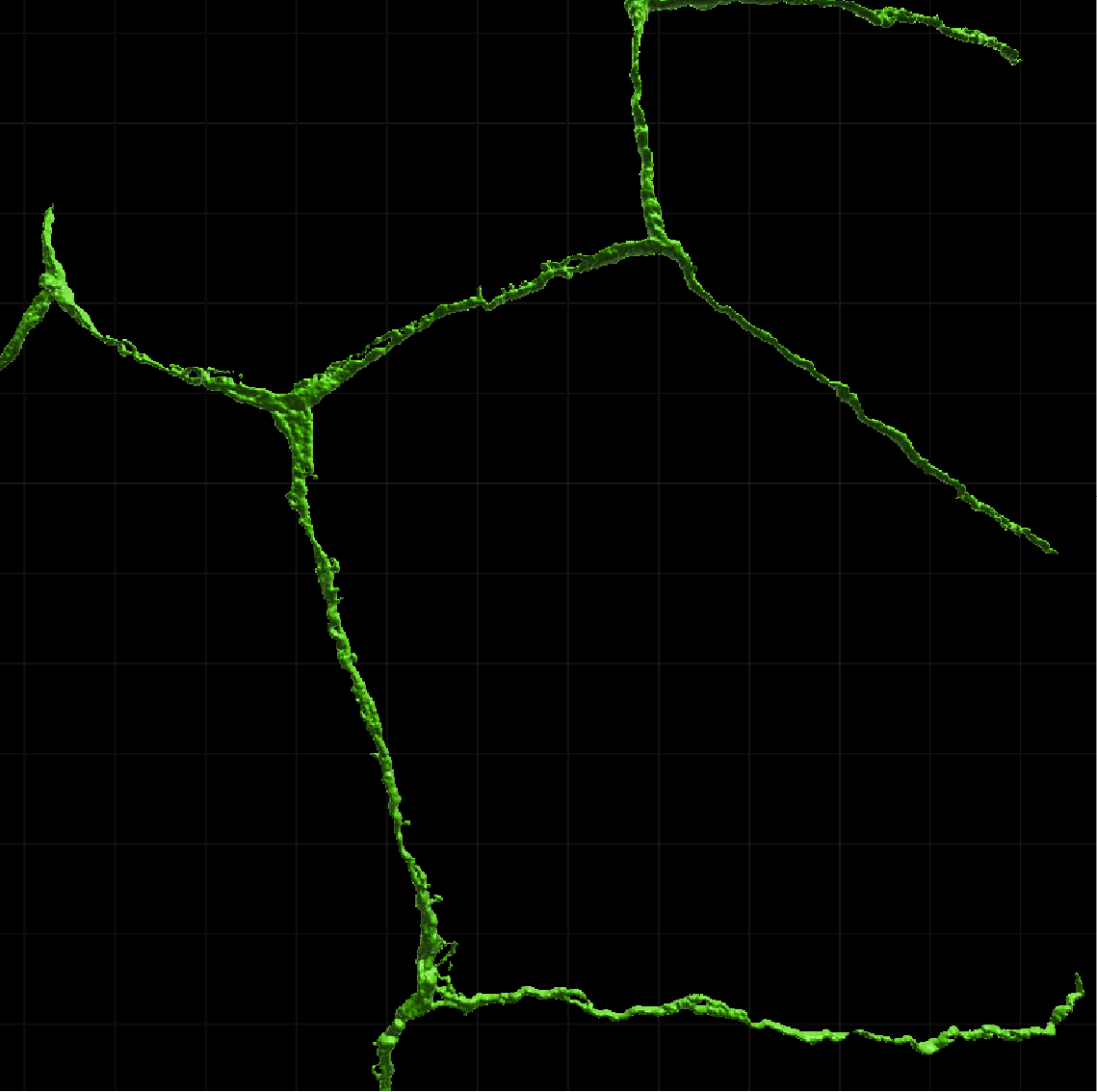

Supplement: Supplementary file 7 — Source data Fig. 3 [file 44319_2026_728_MOESM7_ESM.zip › Source_Data_Fig3/Fig3I-M/Source_Fig3J.tif]

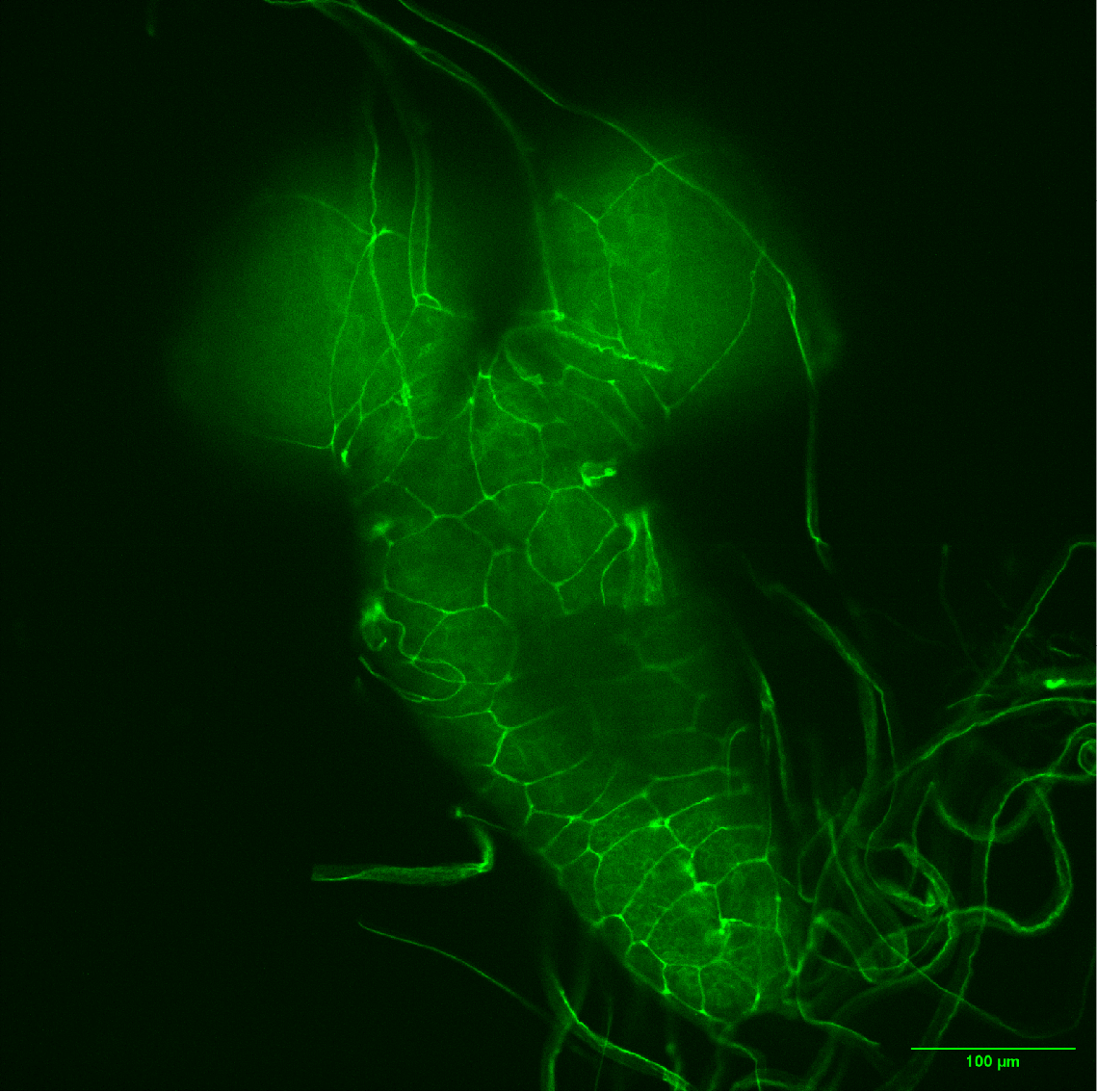

Supplement: Supplementary file 7 — Source data Fig. 3 [file 44319_2026_728_MOESM7_ESM.zip › Source_Data_Fig3/Fig3I-M/Source_Fig3I.tif]

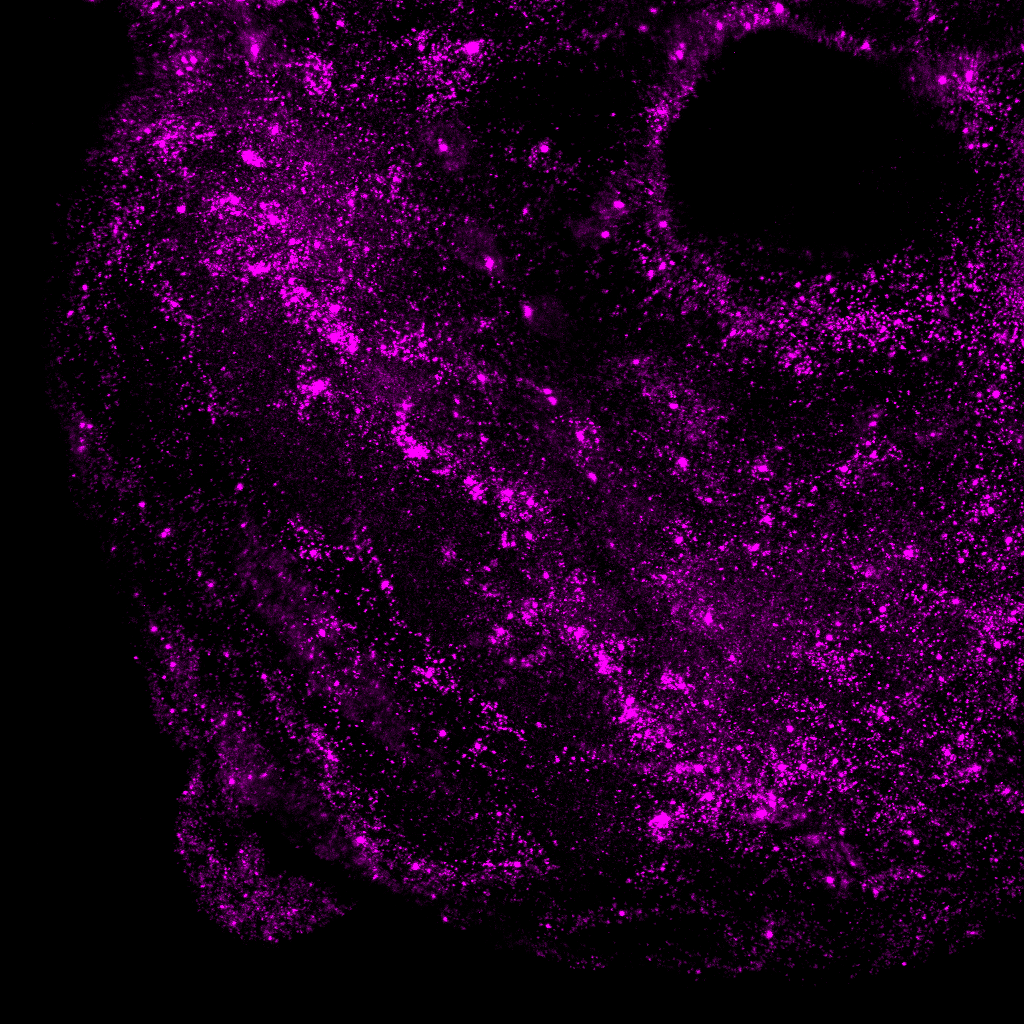

Supplement: Supplementary file 7 — Source data Fig. 3 [file 44319_2026_728_MOESM7_ESM.zip › Source_Data_Fig3/Fig3C-D/MAX_20251016_MoodyG4UASmCD8GFP_wL3_CNS_ISH_DAPI_647NimA_488GFP_561Repo.lif - 1_63X_Repo.tif]

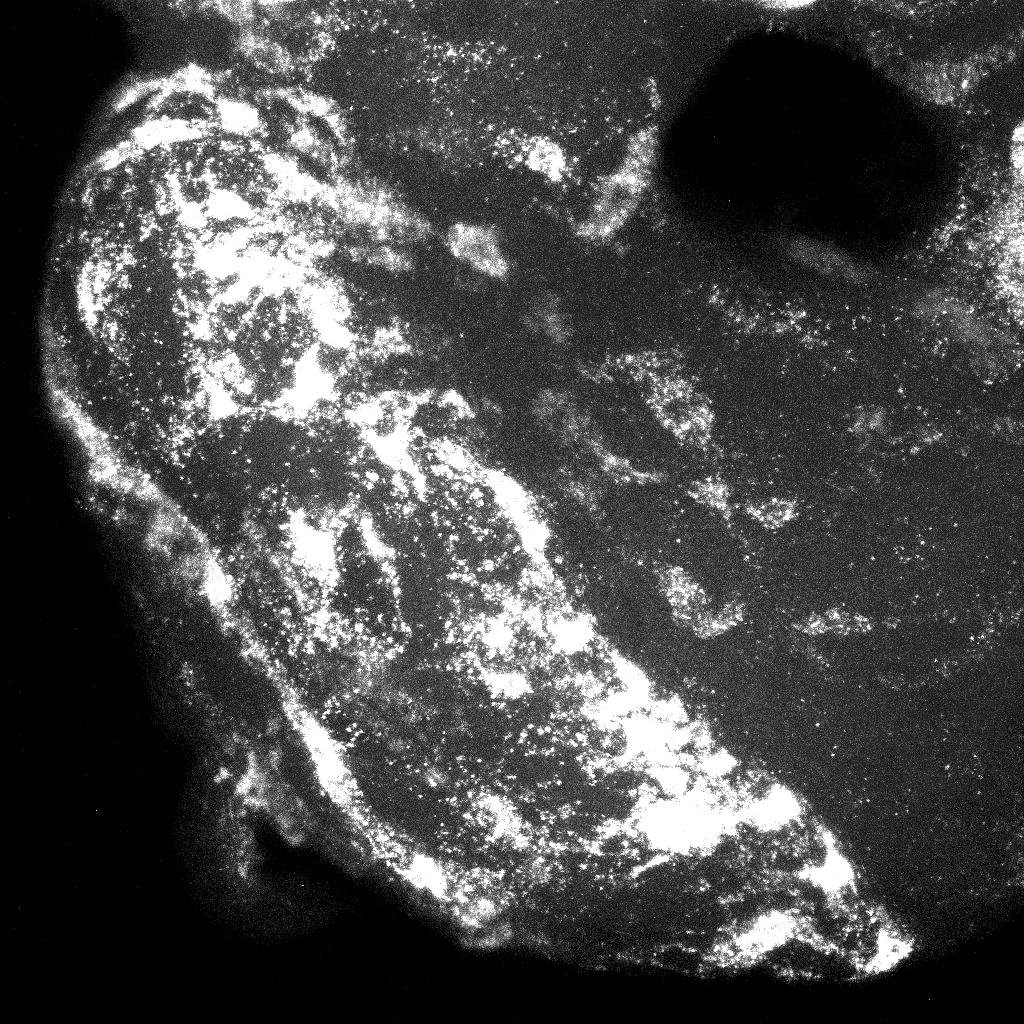

Supplement: Supplementary file 7 — Source data Fig. 3 [file 44319_2026_728_MOESM7_ESM.zip › Source_Data_Fig3/Fig3C-D/MAX_20251016_MoodyG4UASmCD8GFP_wL3_CNS_ISH_DAPI_647NimA_488GFP_561Repo.lif - 1_63X_NimA.tif]

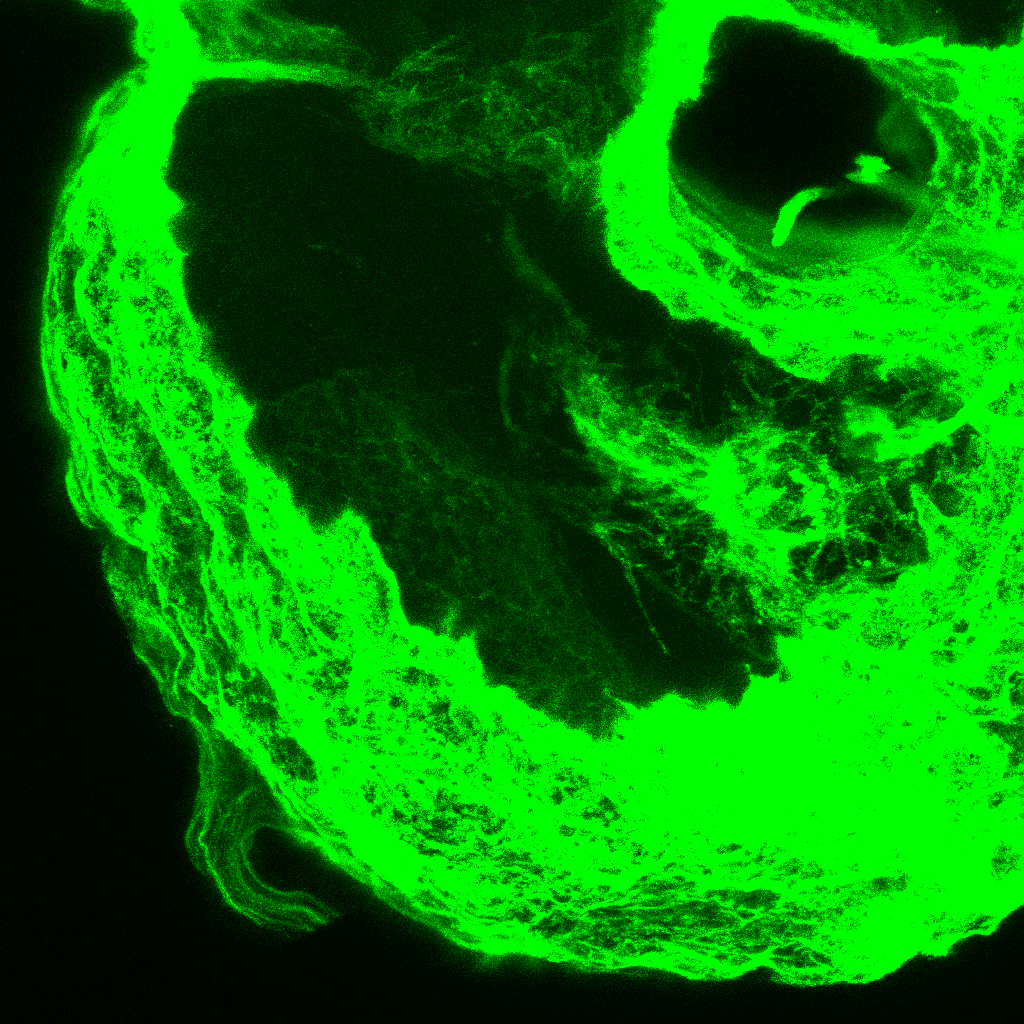

Supplement: Supplementary file 7 — Source data Fig. 3 [file 44319_2026_728_MOESM7_ESM.zip › Source_Data_Fig3/Fig3C-D/MAX_20251016_MoodyG4UASmCD8GFP_wL3_CNS_ISH_DAPI_647NimA_488GFP_561Repo.lif - 1_63X_GFP.tif]

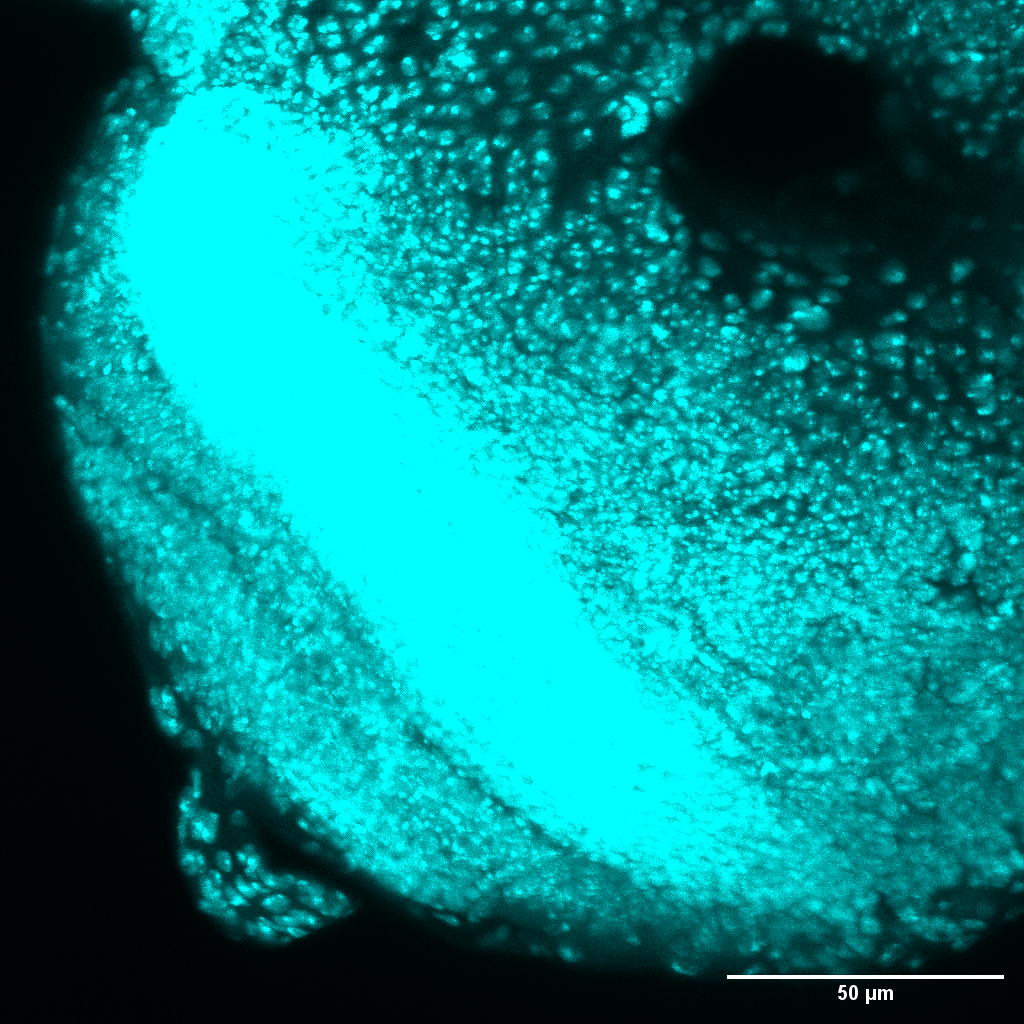

Supplement: Supplementary file 7 — Source data Fig. 3 [file 44319_2026_728_MOESM7_ESM.zip › Source_Data_Fig3/Fig3C-D/MAX_20251016_MoodyG4UASmCD8GFP_wL3_CNS_ISH_DAPI_647NimA_488GFP_561Repo.lif - 1_63X_DAPI.tif]

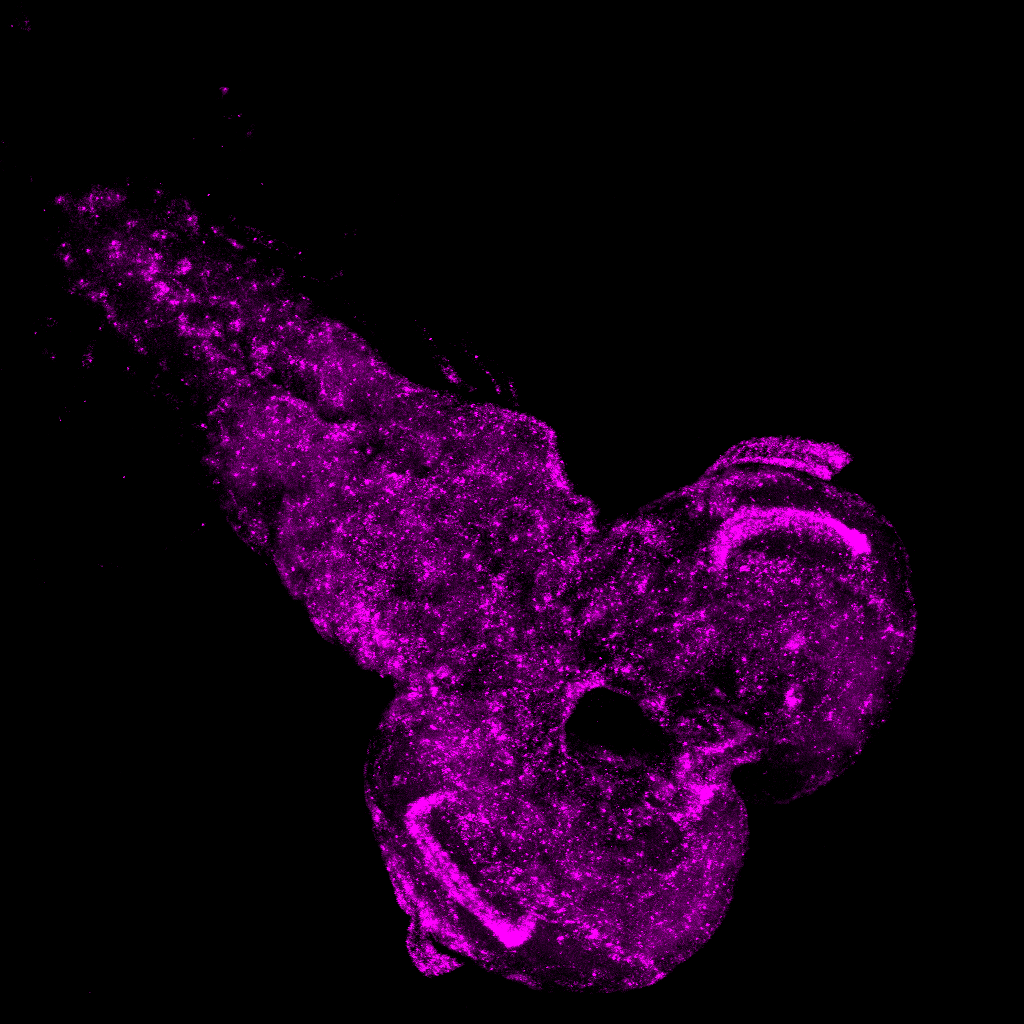

Supplement: Supplementary file 7 — Source data Fig. 3 [file 44319_2026_728_MOESM7_ESM.zip › Source_Data_Fig3/Fig3C-D/MAX_20251016_MoodyG4UASmCD8GFP_wL3_CNS_ISH_DAPI_647NimA_488GFP_561Repo.lif - 1_20X_Repo.tif]

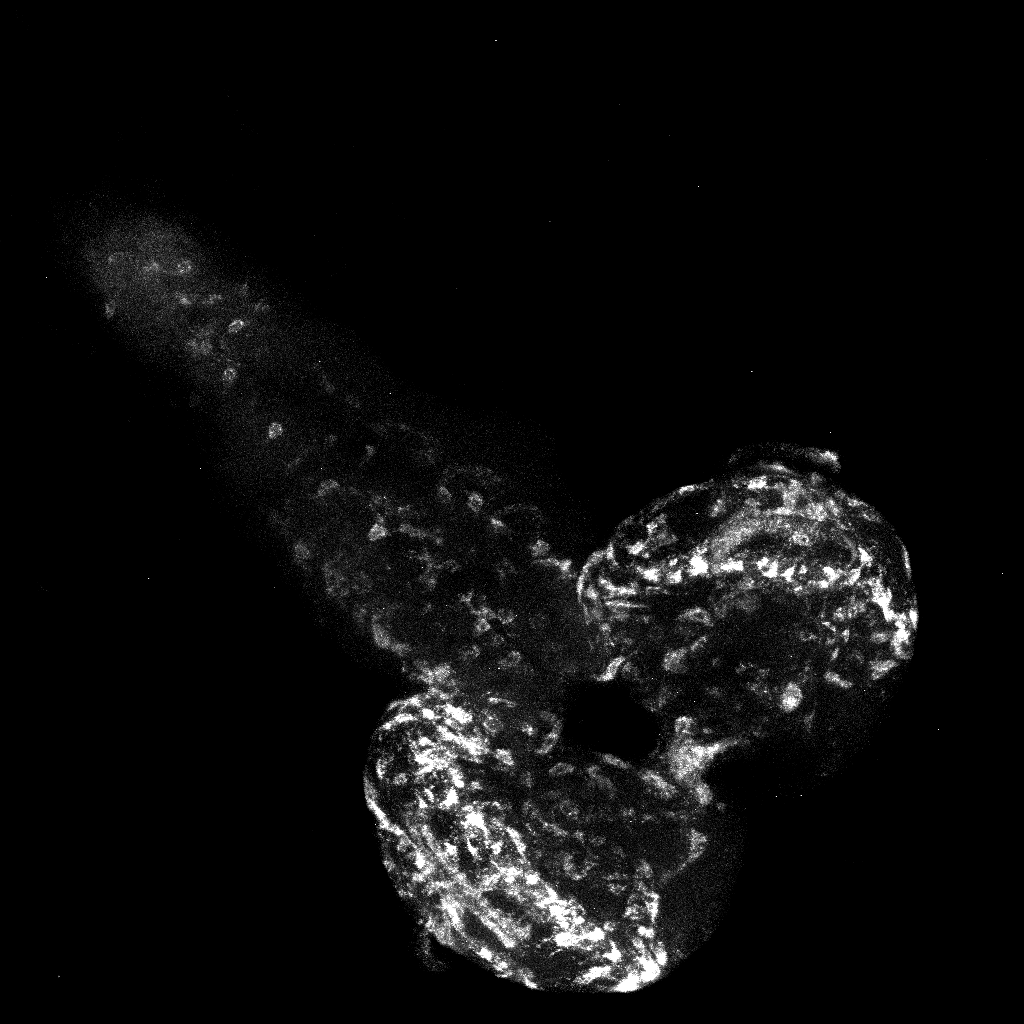

Supplement: Supplementary file 7 — Source data Fig. 3 [file 44319_2026_728_MOESM7_ESM.zip › Source_Data_Fig3/Fig3C-D/MAX_20251016_MoodyG4UASmCD8GFP_wL3_CNS_ISH_DAPI_647NimA_488GFP_561Repo.lif - 1_20X_NimA.tif]

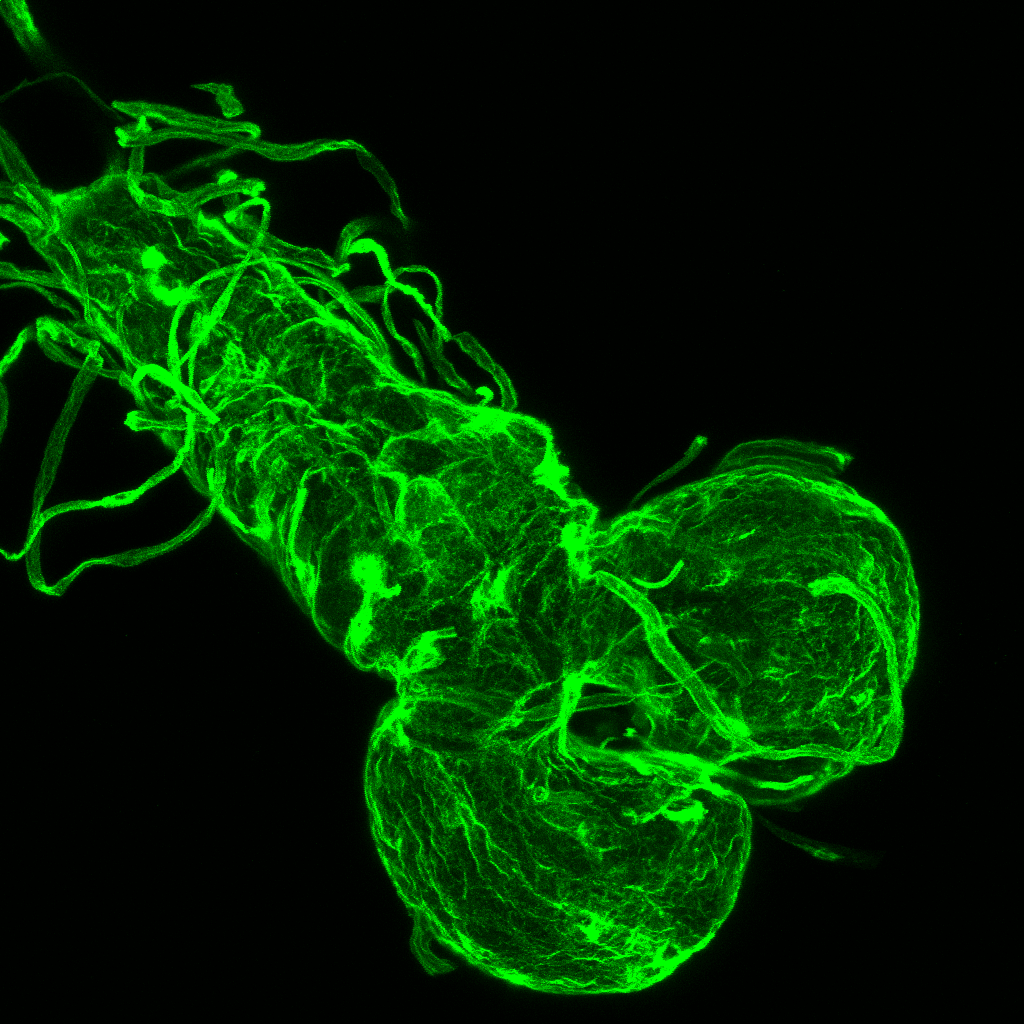

Supplement: Supplementary file 7 — Source data Fig. 3 [file 44319_2026_728_MOESM7_ESM.zip › Source_Data_Fig3/Fig3C-D/MAX_20251016_MoodyG4UASmCD8GFP_wL3_CNS_ISH_DAPI_647NimA_488GFP_561Repo.lif - 1_20X_GFP.tif]

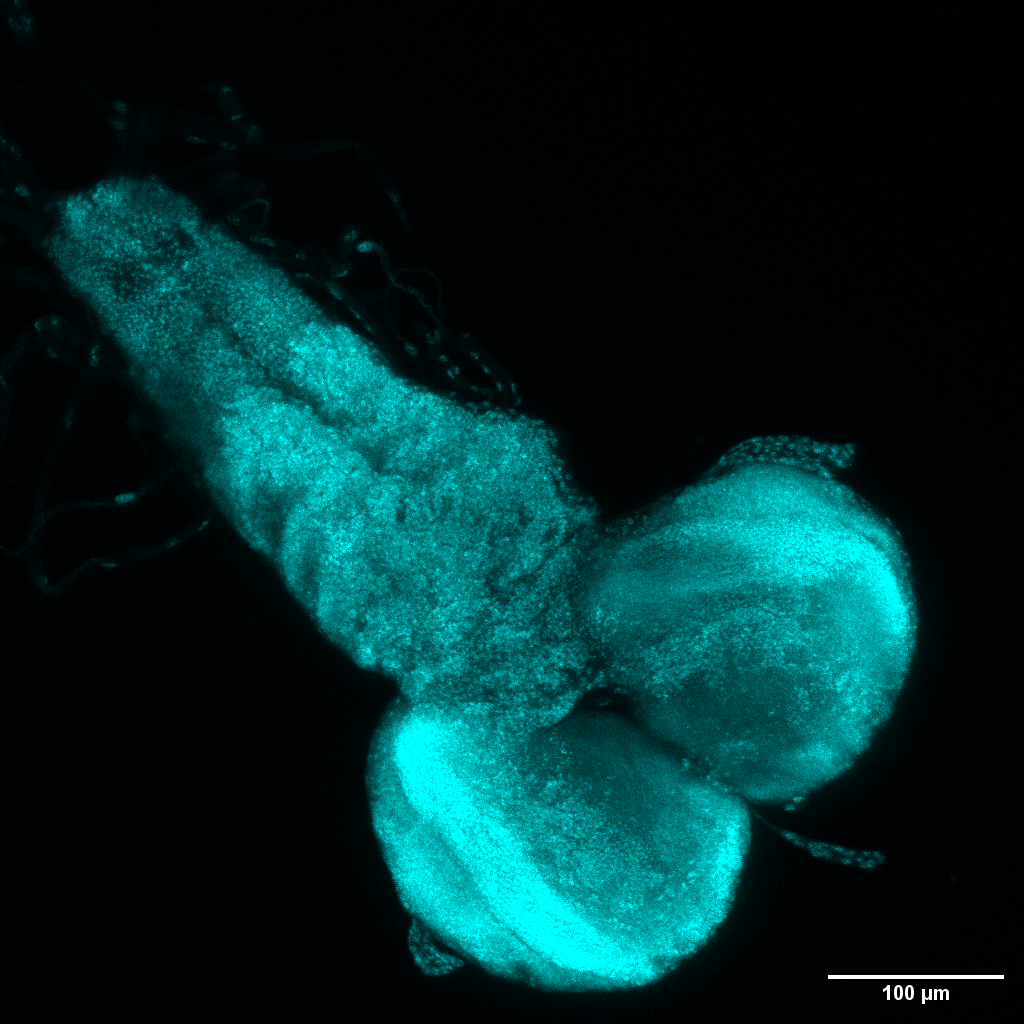

Supplement: Supplementary file 7 — Source data Fig. 3 [file 44319_2026_728_MOESM7_ESM.zip › Source_Data_Fig3/Fig3C-D/MAX_20251016_MoodyG4UASmCD8GFP_wL3_CNS_ISH_DAPI_647NimA_488GFP_561Repo.lif - 1_20X_DAPI.tif]

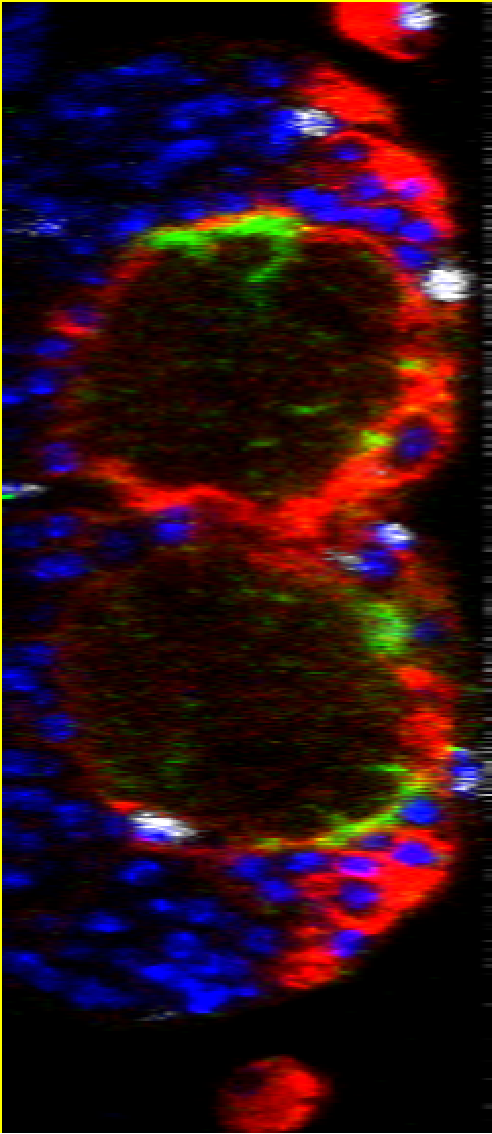

Supplement: Supplementary file 7 — Source data Fig. 3 [file 44319_2026_728_MOESM7_ESM.zip › Source_Data_Fig3/Fig3A-B/Source_Fig3Bz.tif]

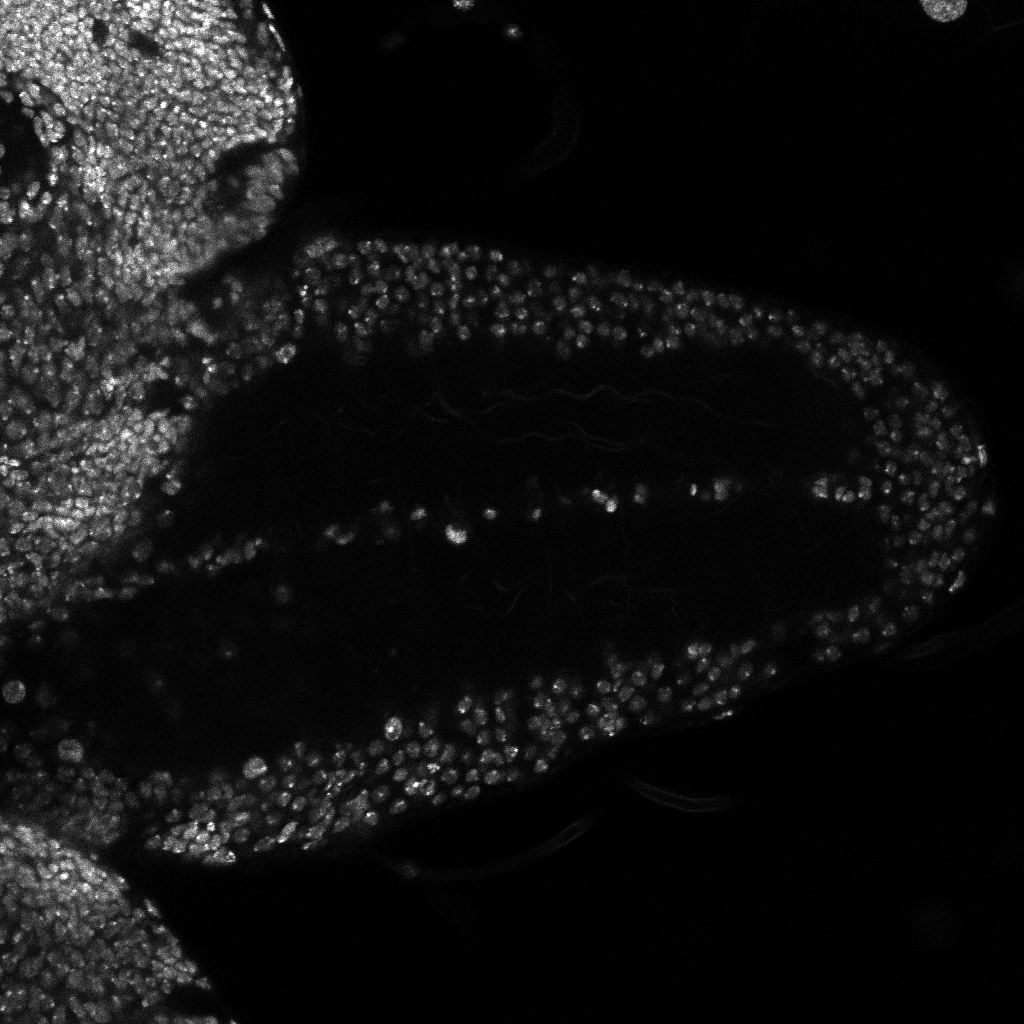

Supplement: Supplementary file 7 — Source data Fig. 3 [file 44319_2026_728_MOESM7_ESM.zip › Source_Data_Fig3/Fig3A-B/Source_Fig3B.tif]

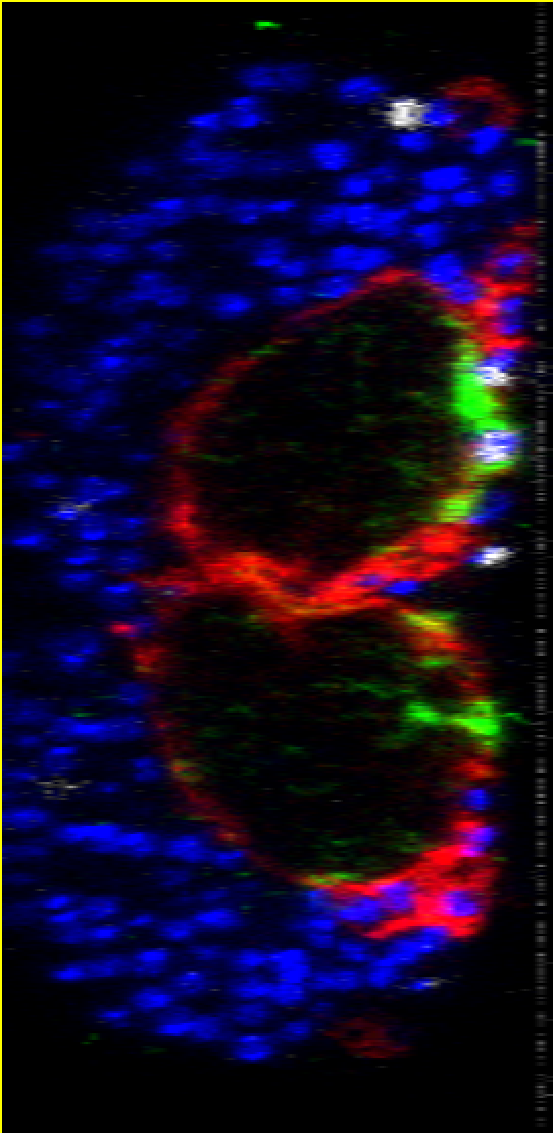

Supplement: Supplementary file 7 — Source data Fig. 3 [file 44319_2026_728_MOESM7_ESM.zip › Source_Data_Fig3/Fig3A-B/Source_Fig3Az.tif]

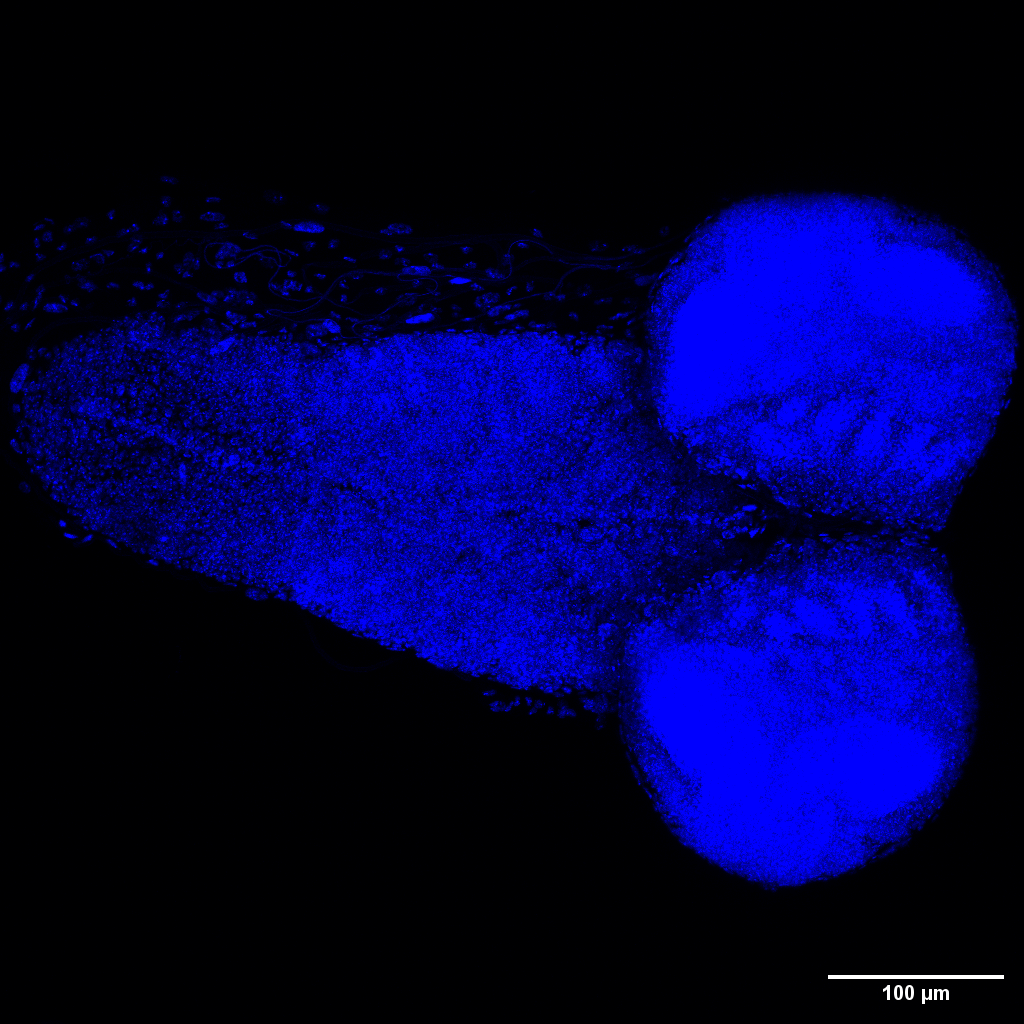

Supplement: Supplementary file 8 — Source data Fig. 4 [file 44319_2026_728_MOESM8_ESM.zip › Source_Data_Fig4/Fig4AB/MAX_20240719_NimAT2AG4HET_wL3_Fem_CNS_DAPI_A647PH3_FITCdpn_20X.lif - Series002_DAPI.tif]

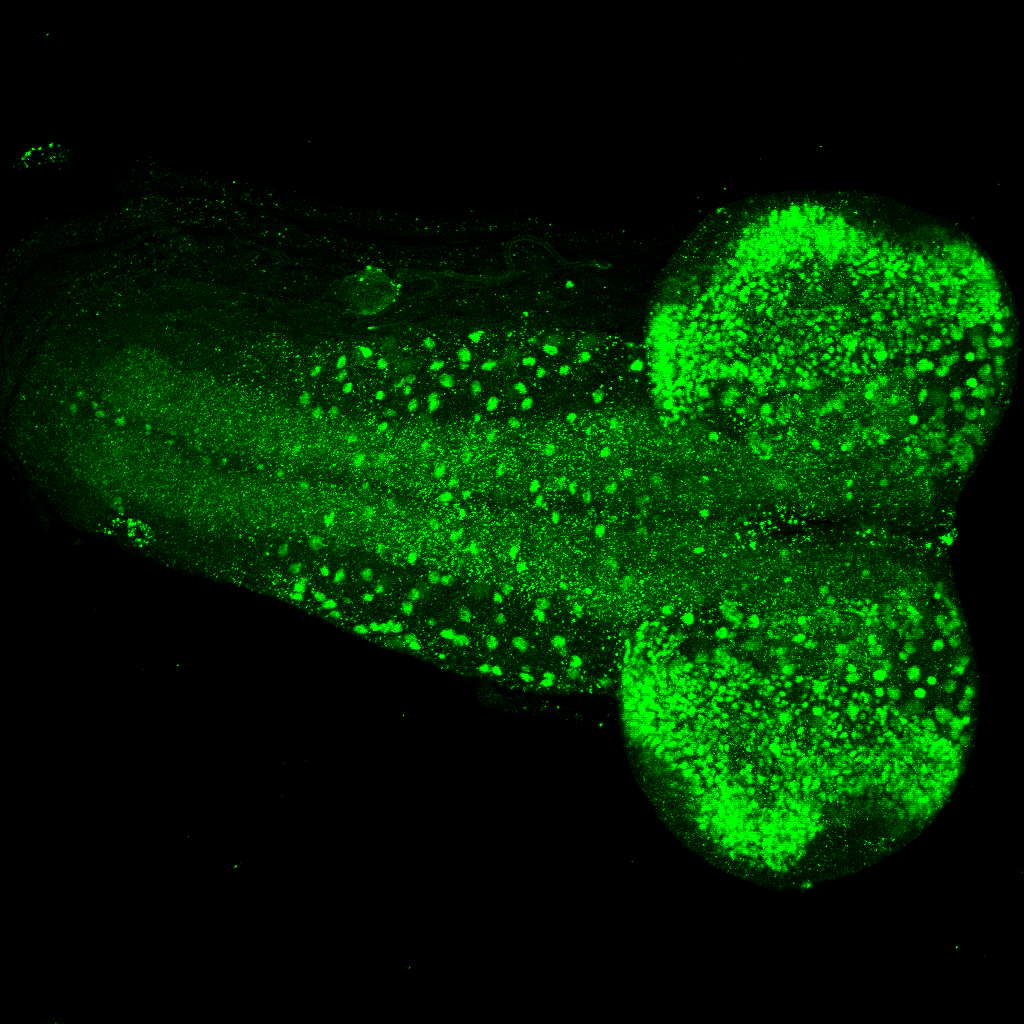

Supplement: Supplementary file 8 — Source data Fig. 4 [file 44319_2026_728_MOESM8_ESM.zip › Source_Data_Fig4/Fig4AB/MAX_20240719_NimAT2AG4HET_wL3_Fem_CNS_DAPI_A647PH3_FITCdpn_20X.lif - Series002_Dpn.tif]

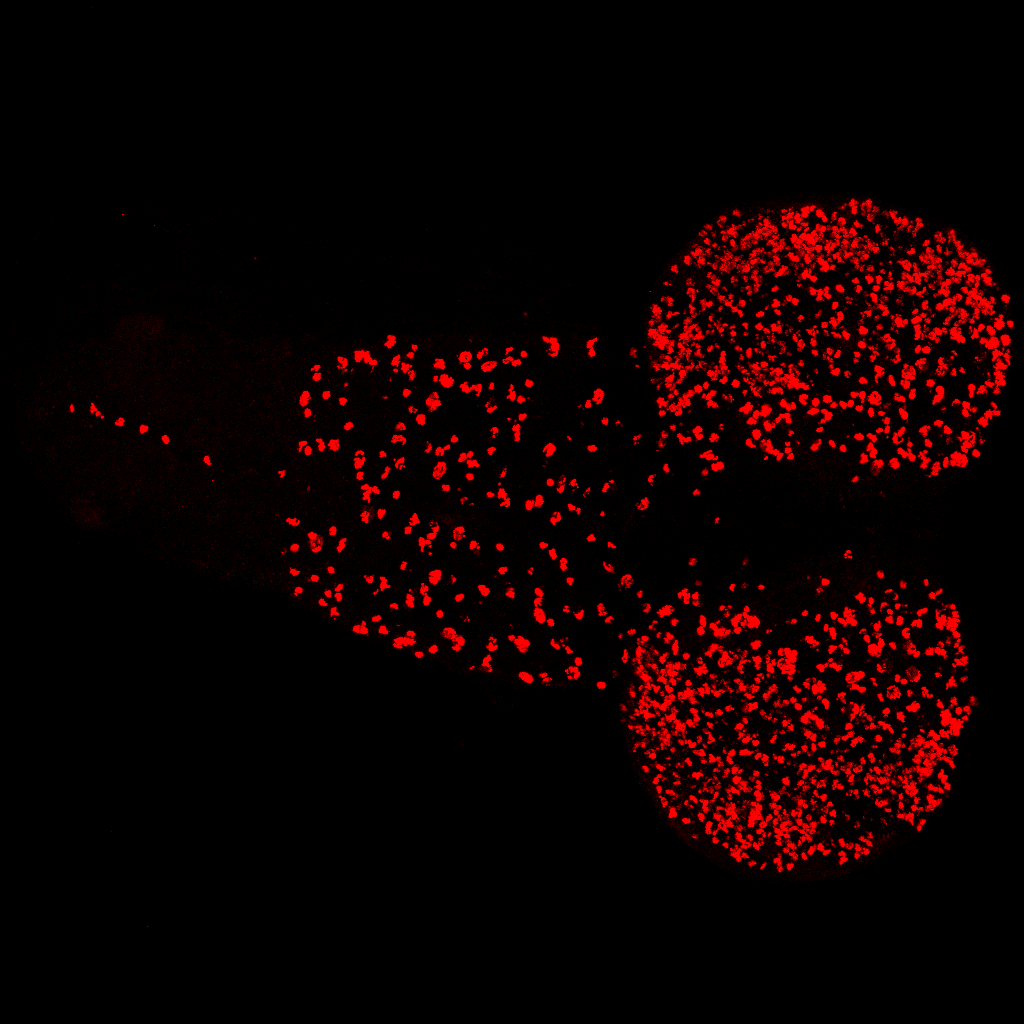

Supplement: Supplementary file 8 — Source data Fig. 4 [file 44319_2026_728_MOESM8_ESM.zip › Source_Data_Fig4/Fig4AB/MAX_20240719_NimAT2AG4HET_wL3_Fem_CNS_DAPI_A647PH3_FITCdpn_20X.lif - Series002_PH3.tif]

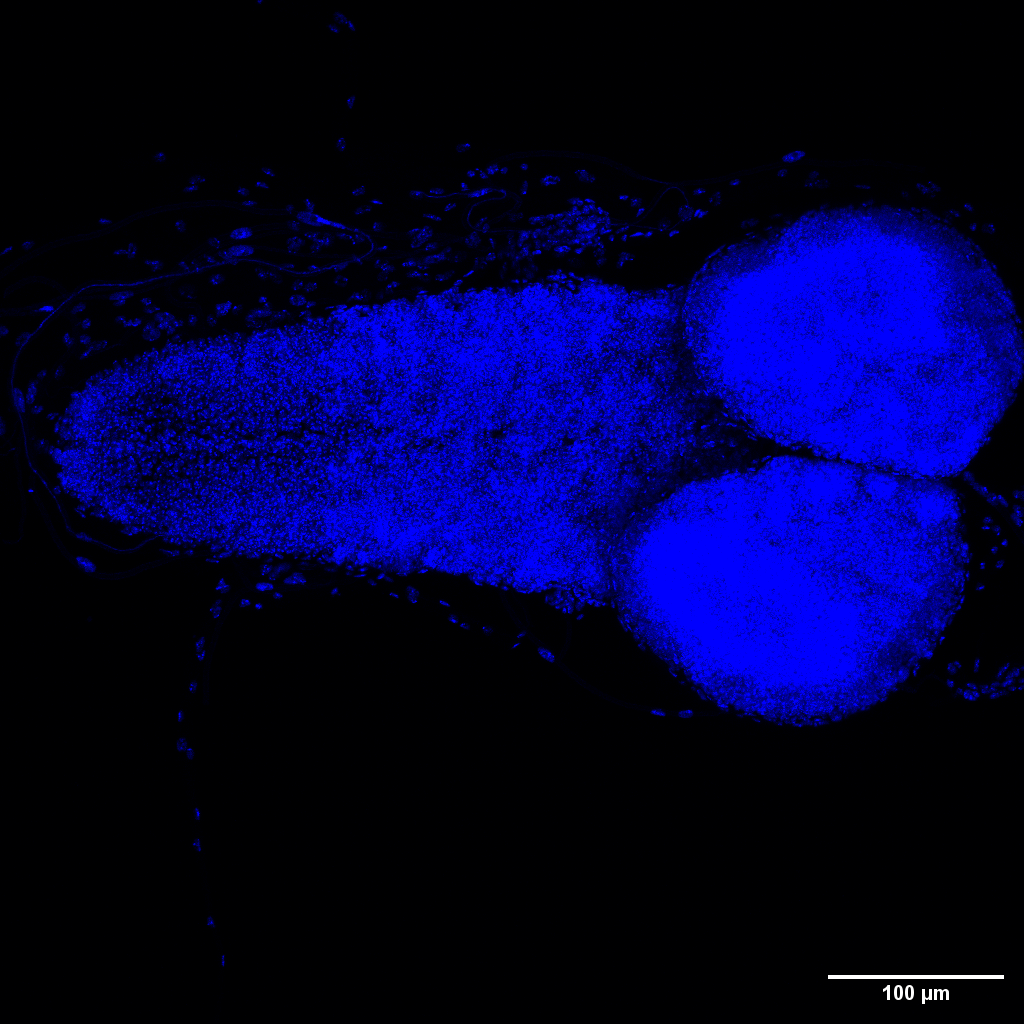

Supplement: Supplementary file 8 — Source data Fig. 4 [file 44319_2026_728_MOESM8_ESM.zip › Source_Data_Fig4/Fig4AB/MAX_20240719_NimAT2AG4HOMO_wL3_Fem_CNS_DAPI_A647PH3_FITCdpn_20X.lif - Series005_DAPI.tif]

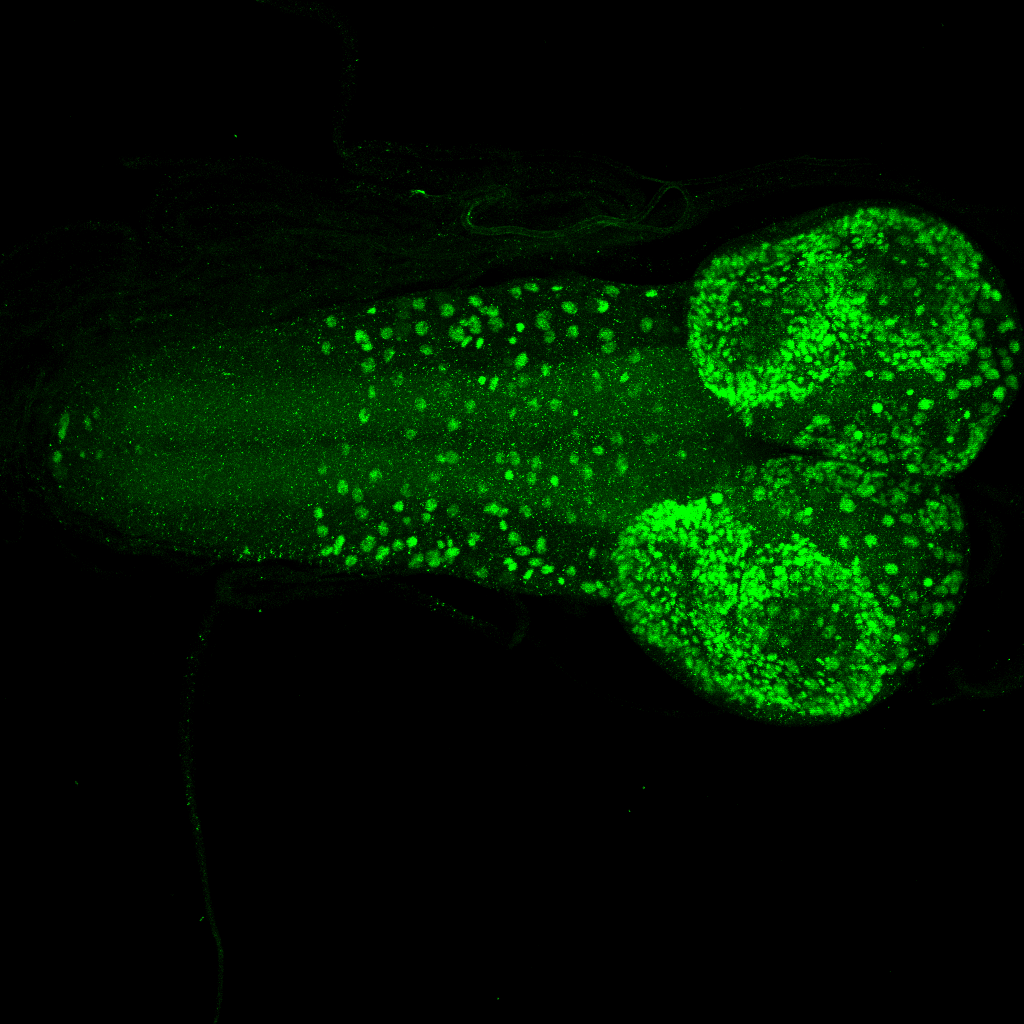

Supplement: Supplementary file 8 — Source data Fig. 4 [file 44319_2026_728_MOESM8_ESM.zip › Source_Data_Fig4/Fig4AB/MAX_20240719_NimAT2AG4HOMO_wL3_Fem_CNS_DAPI_A647PH3_FITCdpn_20X.lif - Series005_Dpn.tif]

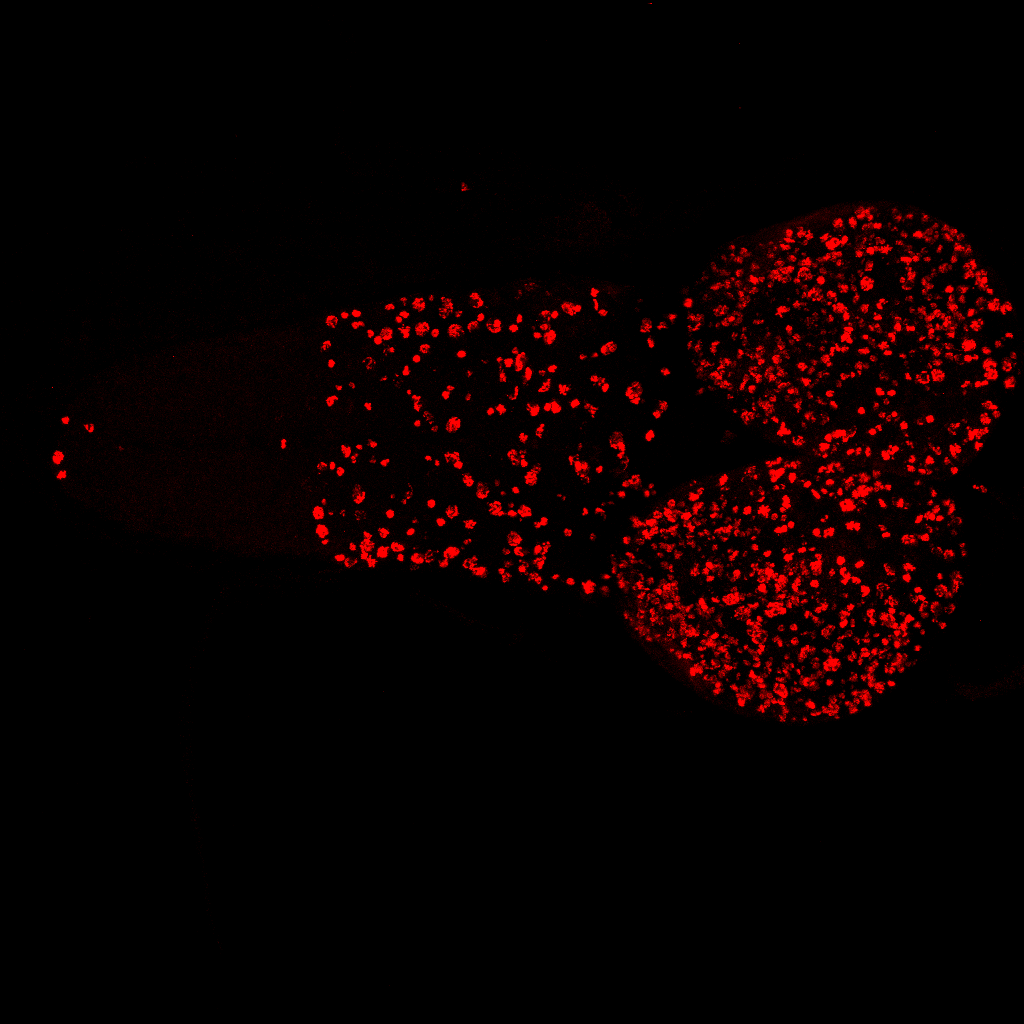

Supplement: Supplementary file 8 — Source data Fig. 4 [file 44319_2026_728_MOESM8_ESM.zip › Source_Data_Fig4/Fig4AB/MAX_20240719_NimAT2AG4HOMO_wL3_Fem_CNS_DAPI_A647PH3_FITCdpn_20X.lif - Series005_PH3.tif]

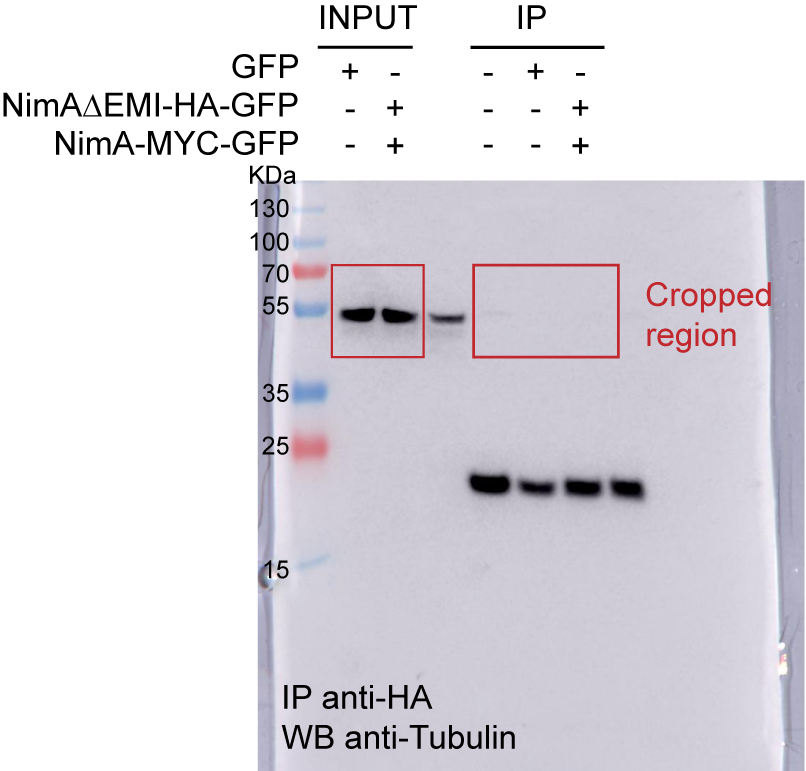

Supplement: Supplementary file 9 — Source data Fig. 5 [file 44319_2026_728_MOESM9_ESM.zip › Source_Data_Fig5/Fig5N/NimADeltaEMIHA_NimAMyc_Co-IP_WB_antiTubulin.tif]

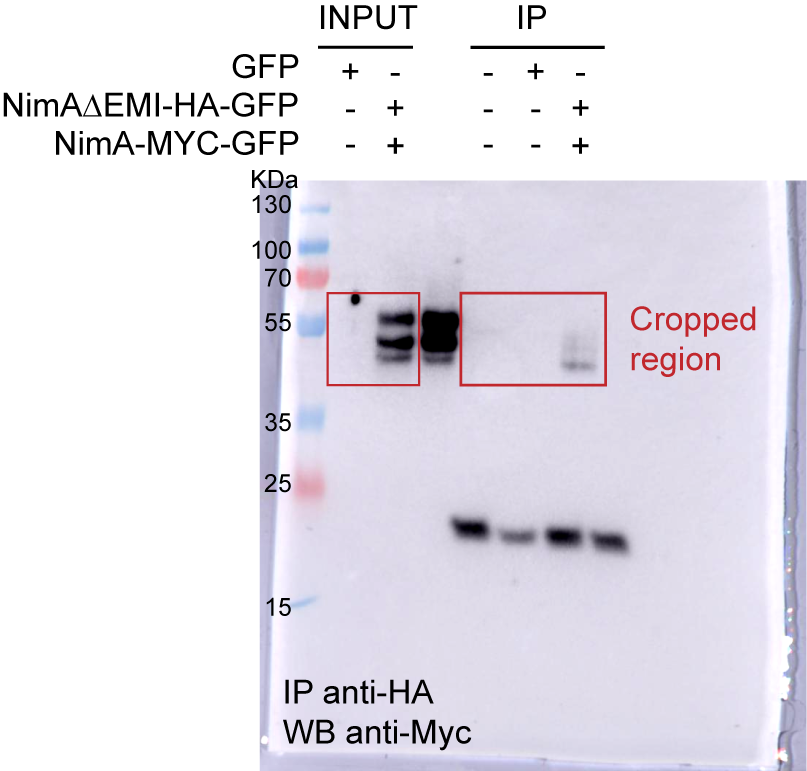

Supplement: Supplementary file 9 — Source data Fig. 5 [file 44319_2026_728_MOESM9_ESM.zip › Source_Data_Fig5/Fig5N/NimADeltaEMIHA_NimAMyc_Co-IP_WB_antiMyc.tif]

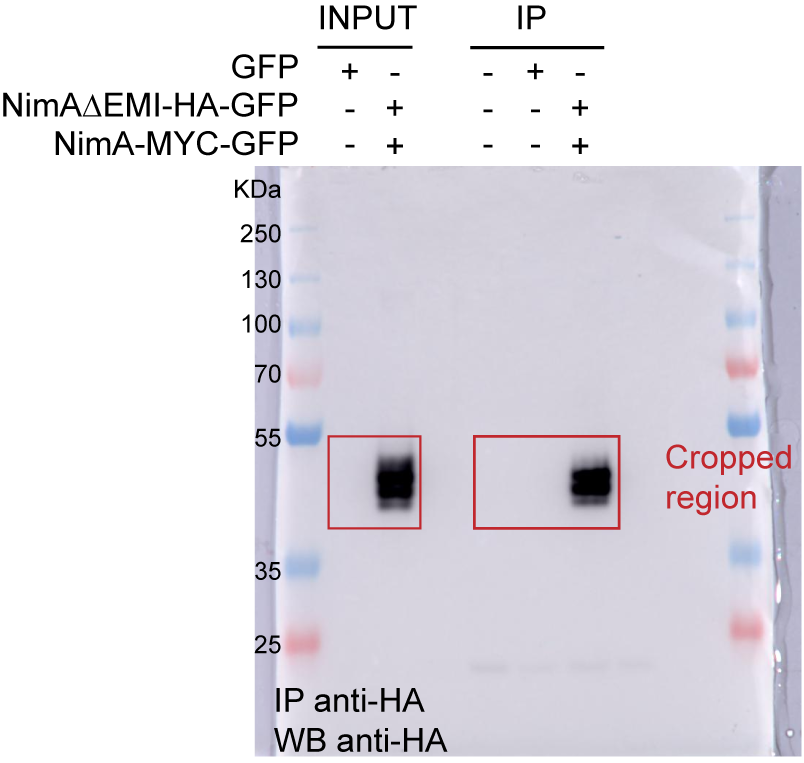

Supplement: Supplementary file 9 — Source data Fig. 5 [file 44319_2026_728_MOESM9_ESM.zip › Source_Data_Fig5/Fig5N/NimADeltaEMIHA_NimAMyc_Co-IP_WB_antiHA.tif]

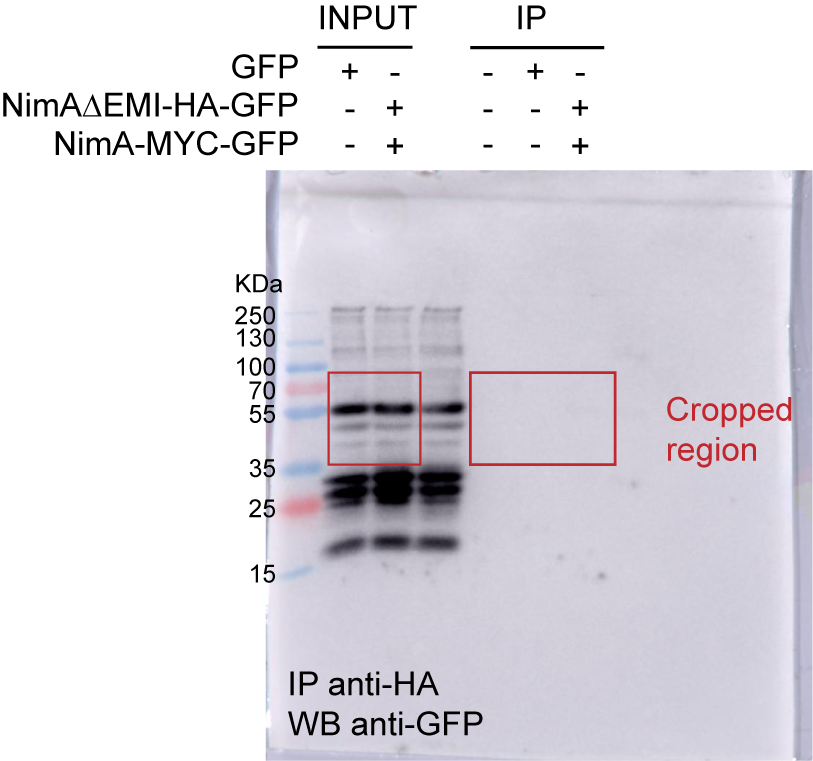

Supplement: Supplementary file 9 — Source data Fig. 5 [file 44319_2026_728_MOESM9_ESM.zip › Source_Data_Fig5/Fig5N/NimADeltaEMIHA_NimAMyc_Co-IP_WB_antiGFP.tif]

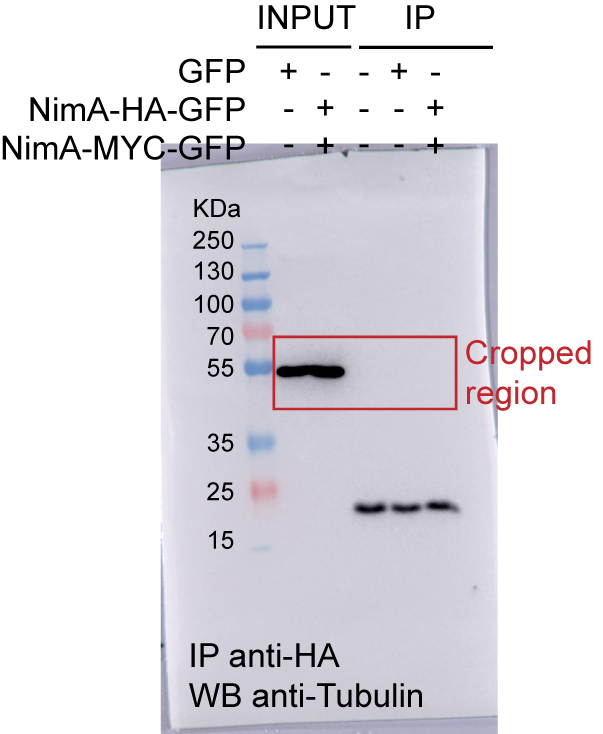

Supplement: Supplementary file 9 — Source data Fig. 5 [file 44319_2026_728_MOESM9_ESM.zip › Source_Data_Fig5/Fig5M/NimAHA_NimAMyc_Co-IP_WB_antiTubulin.tif]

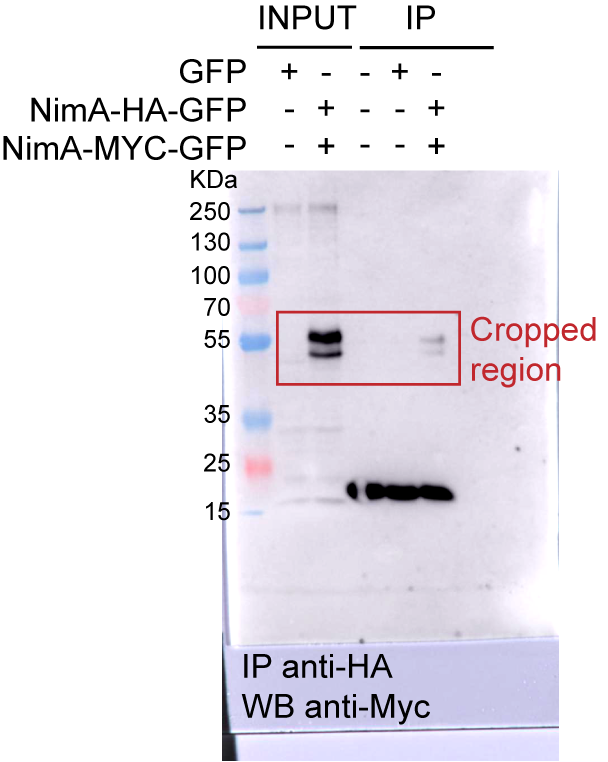

Supplement: Supplementary file 9 — Source data Fig. 5 [file 44319_2026_728_MOESM9_ESM.zip › Source_Data_Fig5/Fig5M/NimAHA_NimAMyc_Co-IP_WB_antiMyc.tif]

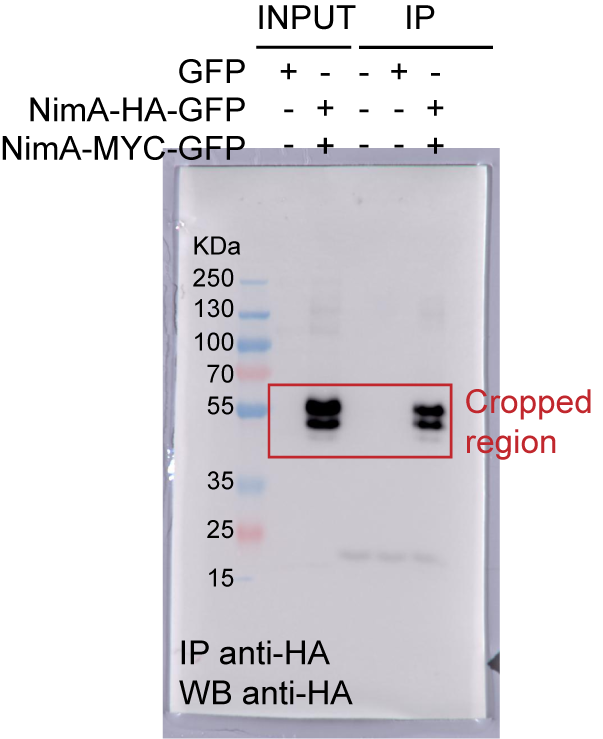

Supplement: Supplementary file 9 — Source data Fig. 5 [file 44319_2026_728_MOESM9_ESM.zip › Source_Data_Fig5/Fig5M/NimAHA_NimAMyc_Co-IP_WB_antiHA.tif]

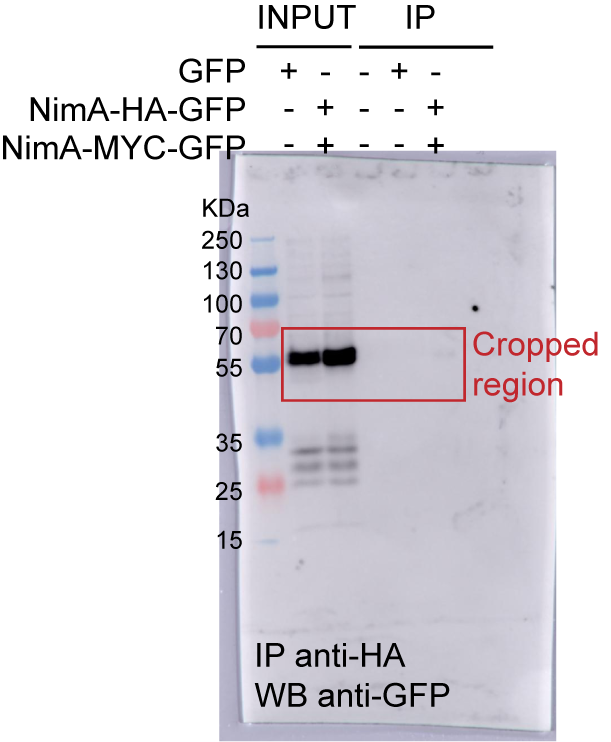

Supplement: Supplementary file 9 — Source data Fig. 5 [file 44319_2026_728_MOESM9_ESM.zip › Source_Data_Fig5/Fig5M/NimAHA_NimAMyc_Co-IP_WB_antiGFP.tif]

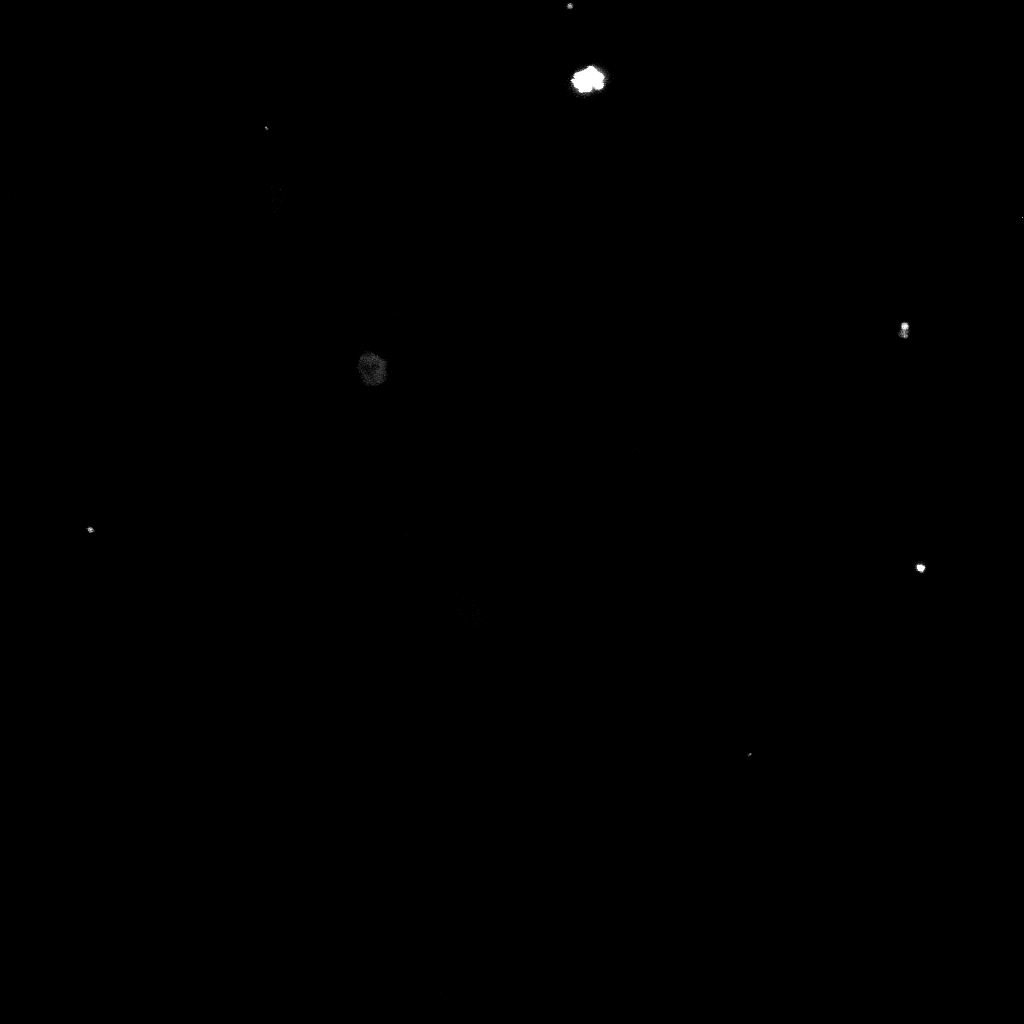

Supplement: Supplementary file 9 — Source data Fig. 5 [file 44319_2026_728_MOESM9_ESM.zip › Source_Data_Fig5/Fig5KL/MAX_20240718_pUASTattbT2AmCD8GFP_S2_DAPI_A647MYC_FITCGFP_CY3HA_63X.lif - Series001_Myc.tif]

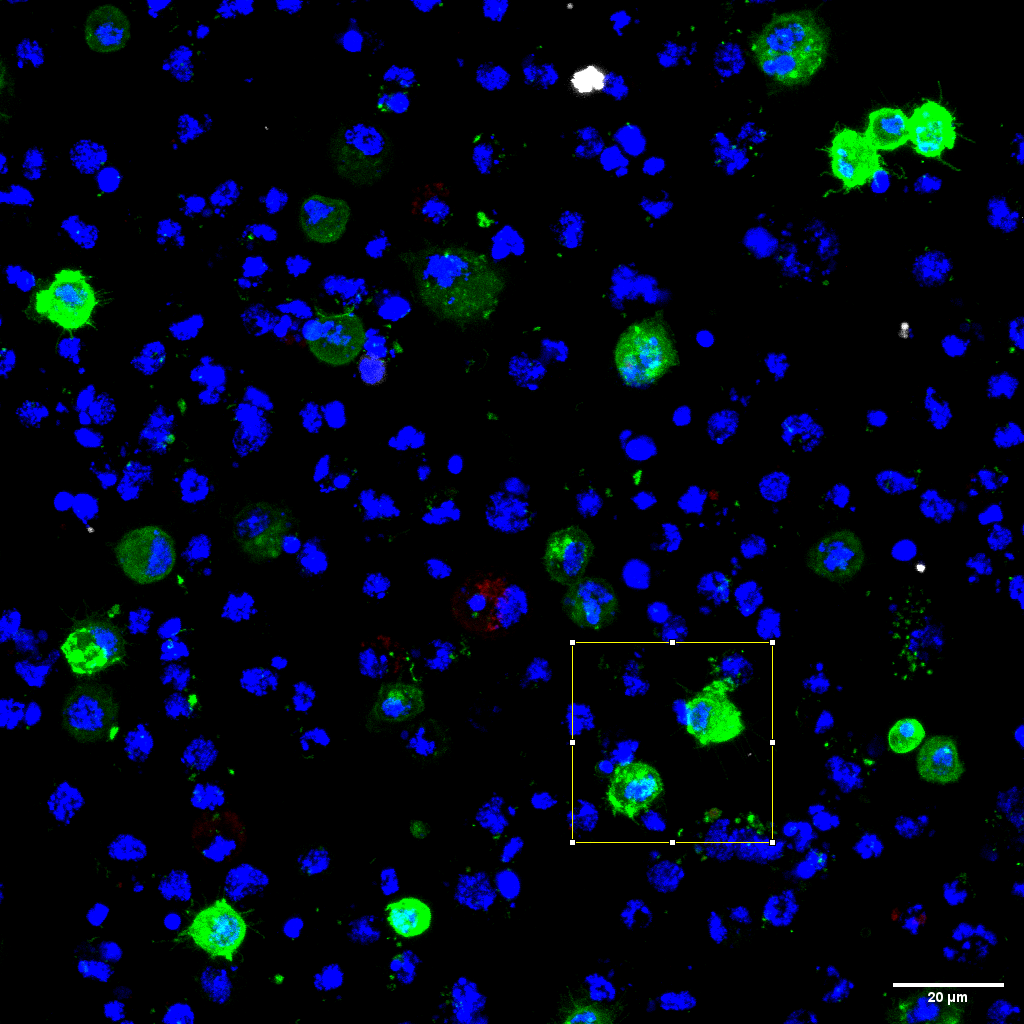

Supplement: Supplementary file 9 — Source data Fig. 5 [file 44319_2026_728_MOESM9_ESM.zip › Source_Data_Fig5/Fig5KL/MAX_20240718_pUASTattbT2AmCD8GFP_S2_DAPI_A647MYC_FITCGFP_CY3HA_63X.lif - Series001_Merge.tif]

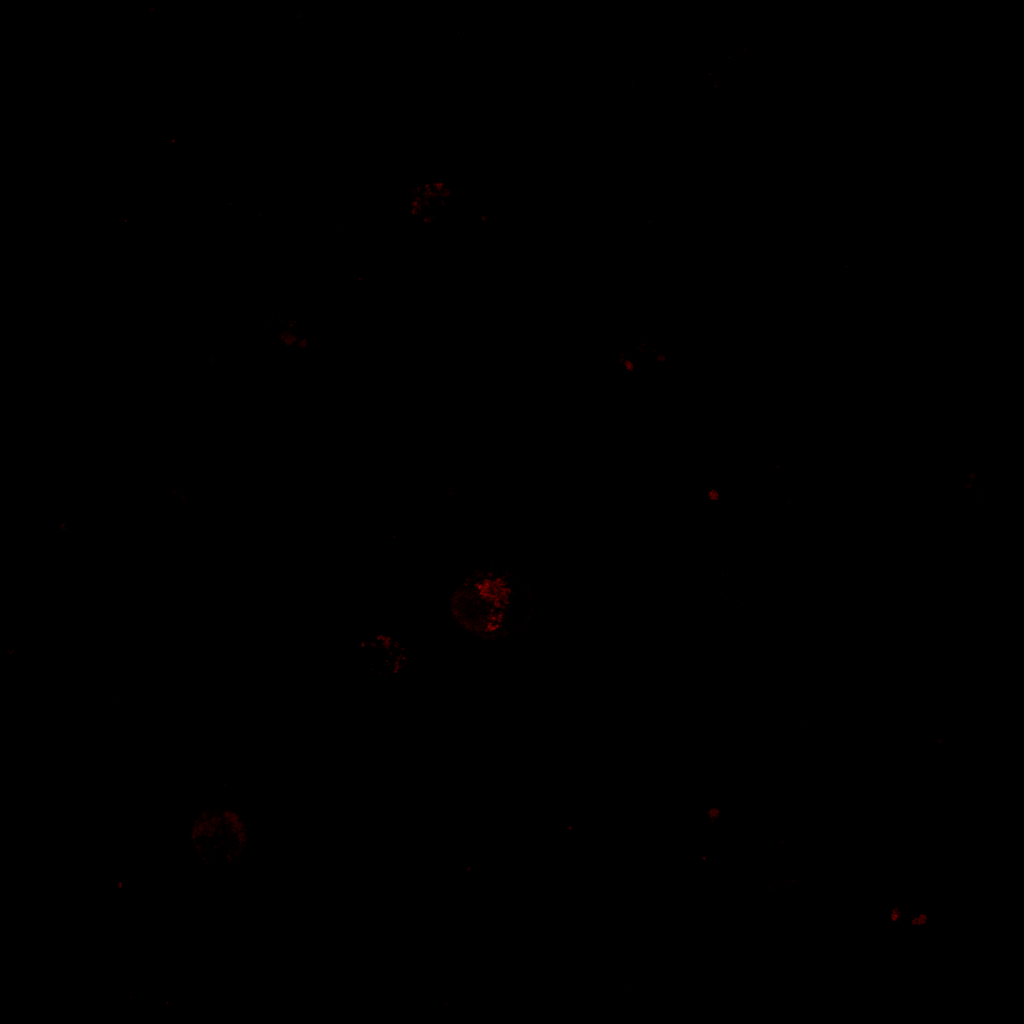

Supplement: Supplementary file 9 — Source data Fig. 5 [file 44319_2026_728_MOESM9_ESM.zip › Source_Data_Fig5/Fig5KL/MAX_20240718_pUASTattbT2AmCD8GFP_S2_DAPI_A647MYC_FITCGFP_CY3HA_63X.lif - Series001_HA.tif]

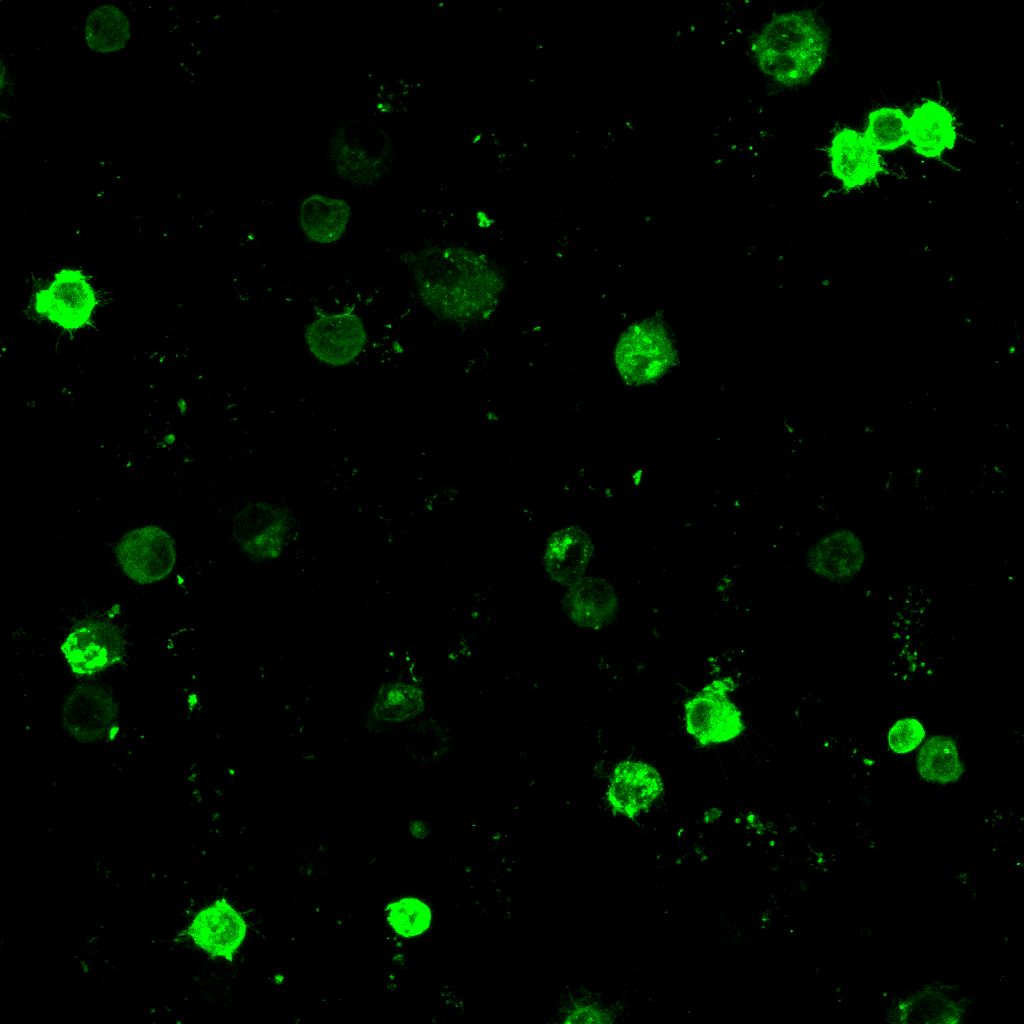

Supplement: Supplementary file 9 — Source data Fig. 5 [file 44319_2026_728_MOESM9_ESM.zip › Source_Data_Fig5/Fig5KL/MAX_20240718_pUASTattbT2AmCD8GFP_S2_DAPI_A647MYC_FITCGFP_CY3HA_63X.lif - Series001_GFP.tif]

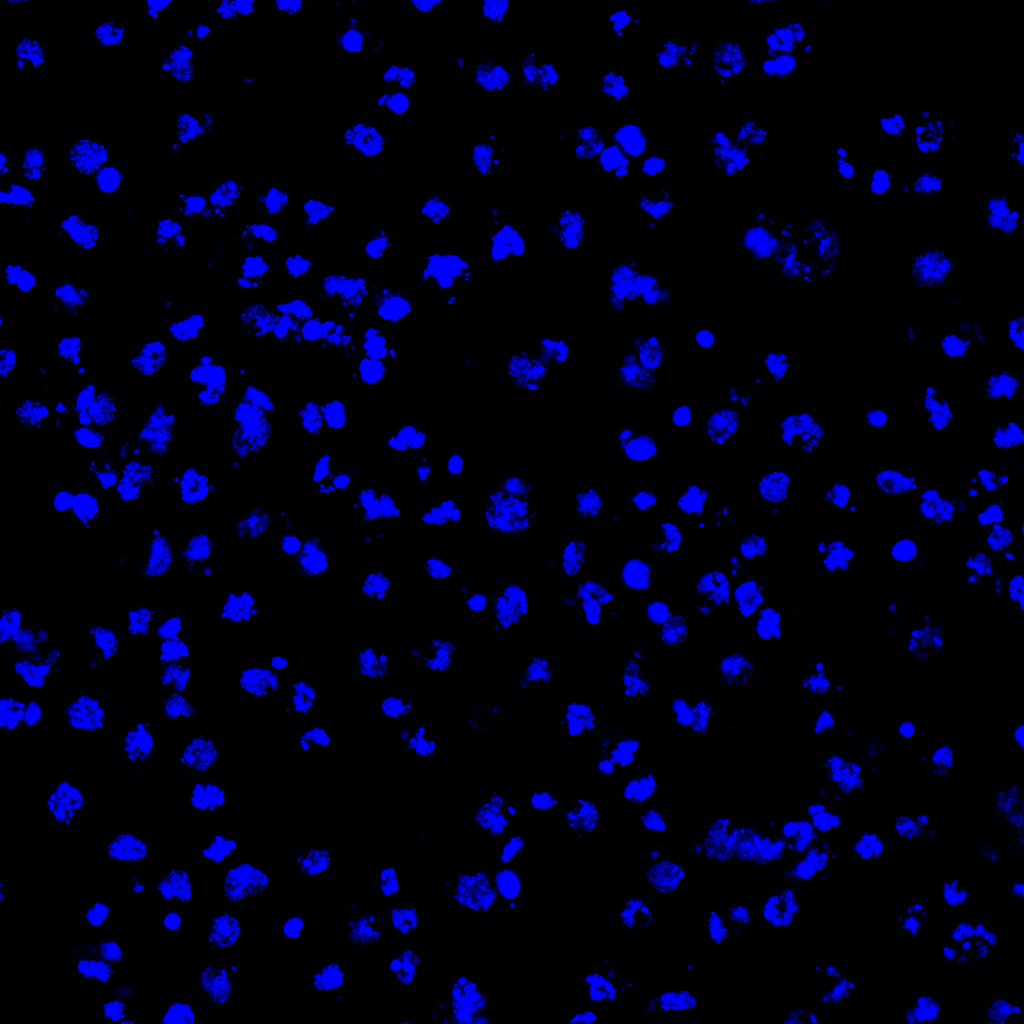

Supplement: Supplementary file 9 — Source data Fig. 5 [file 44319_2026_728_MOESM9_ESM.zip › Source_Data_Fig5/Fig5KL/MAX_20240718_pUASTattbT2AmCD8GFP_S2_DAPI_A647MYC_FITCGFP_CY3HA_63X.lif - Series001_DAPI.tif]

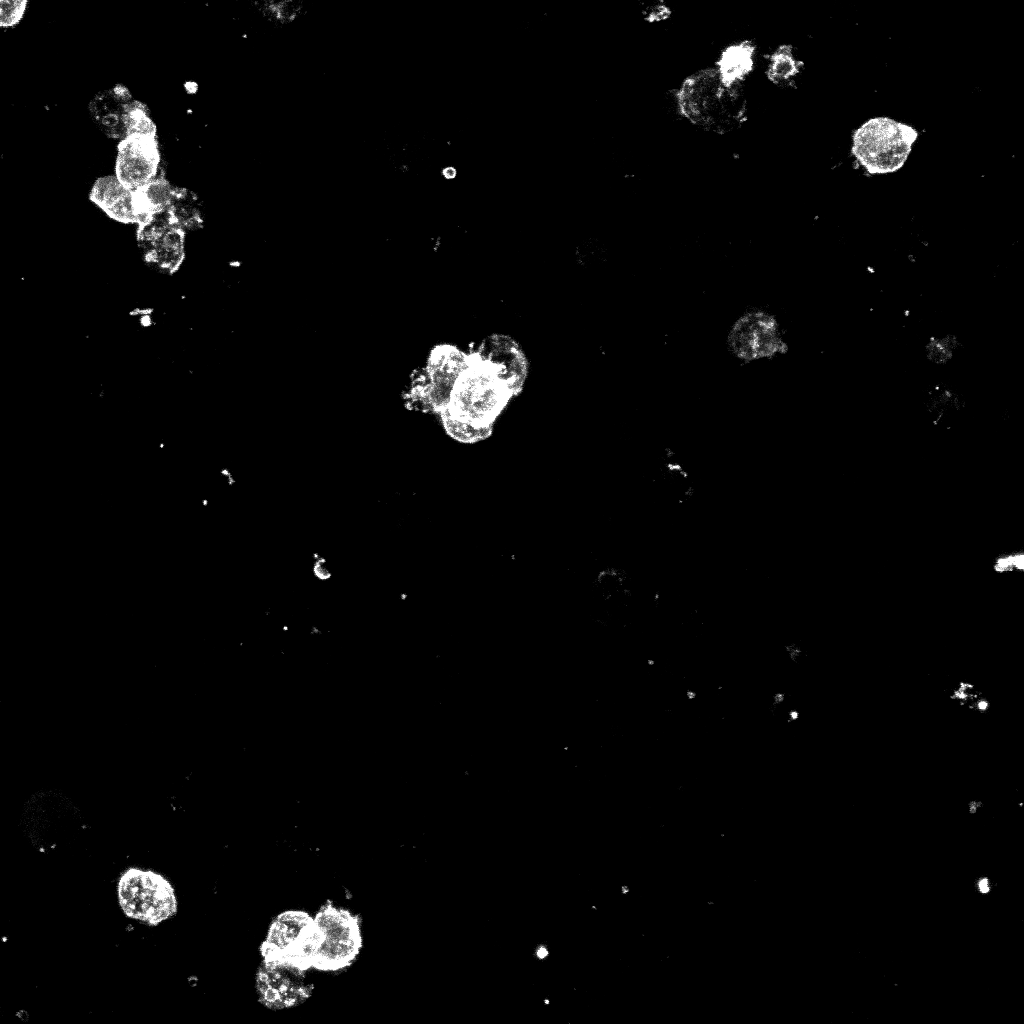

Supplement: Supplementary file 9 — Source data Fig. 5 [file 44319_2026_728_MOESM9_ESM.zip › Source_Data_Fig5/Fig5KL/MAX_20240718_pUASTattbNimAHA-NimAMYC-T2AmCD8GFP_S2_DAPI_A647MYC_FITCGFP_CY3HA_63X.lif - Series001_Myc.tif]

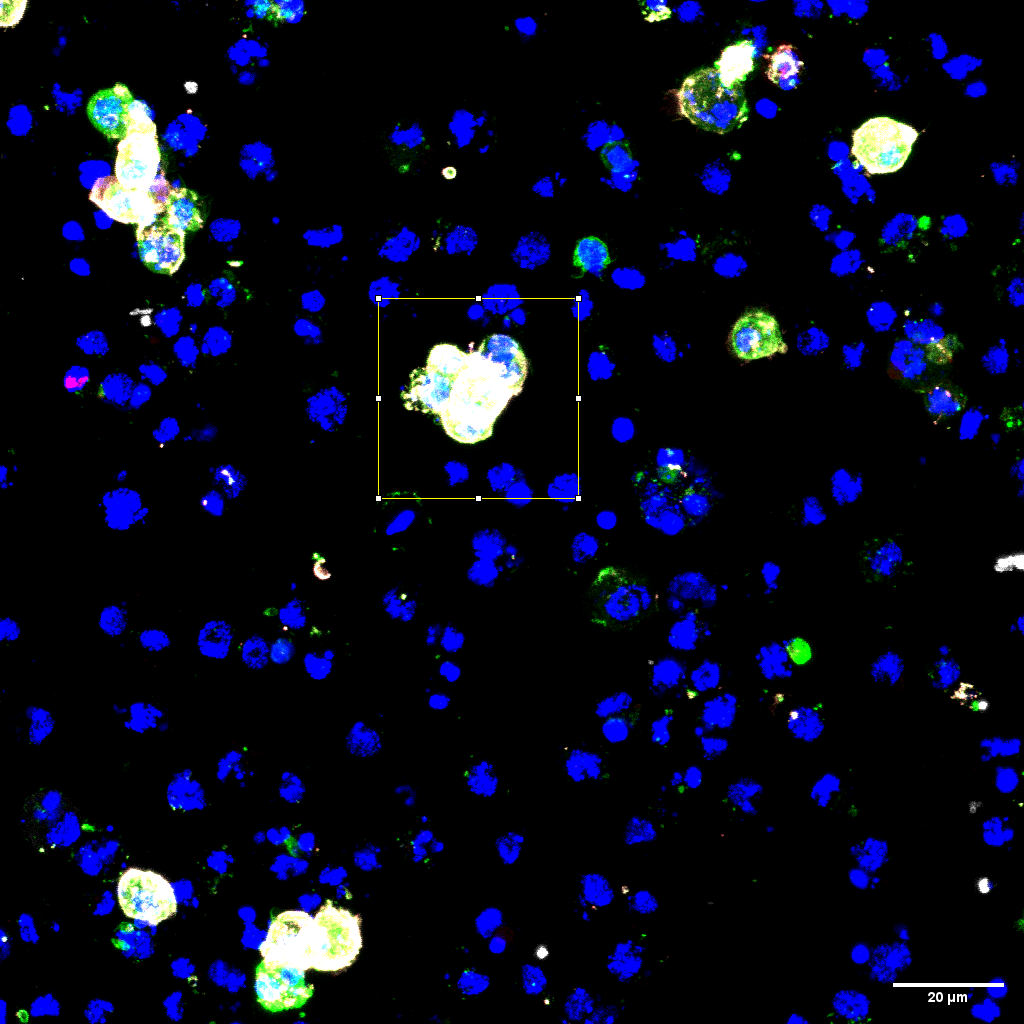

Supplement: Supplementary file 9 — Source data Fig. 5 [file 44319_2026_728_MOESM9_ESM.zip › Source_Data_Fig5/Fig5KL/MAX_20240718_pUASTattbNimAHA-NimAMYC-T2AmCD8GFP_S2_DAPI_A647MYC_FITCGFP_CY3HA_63X.lif - Series001_Merge.tif]

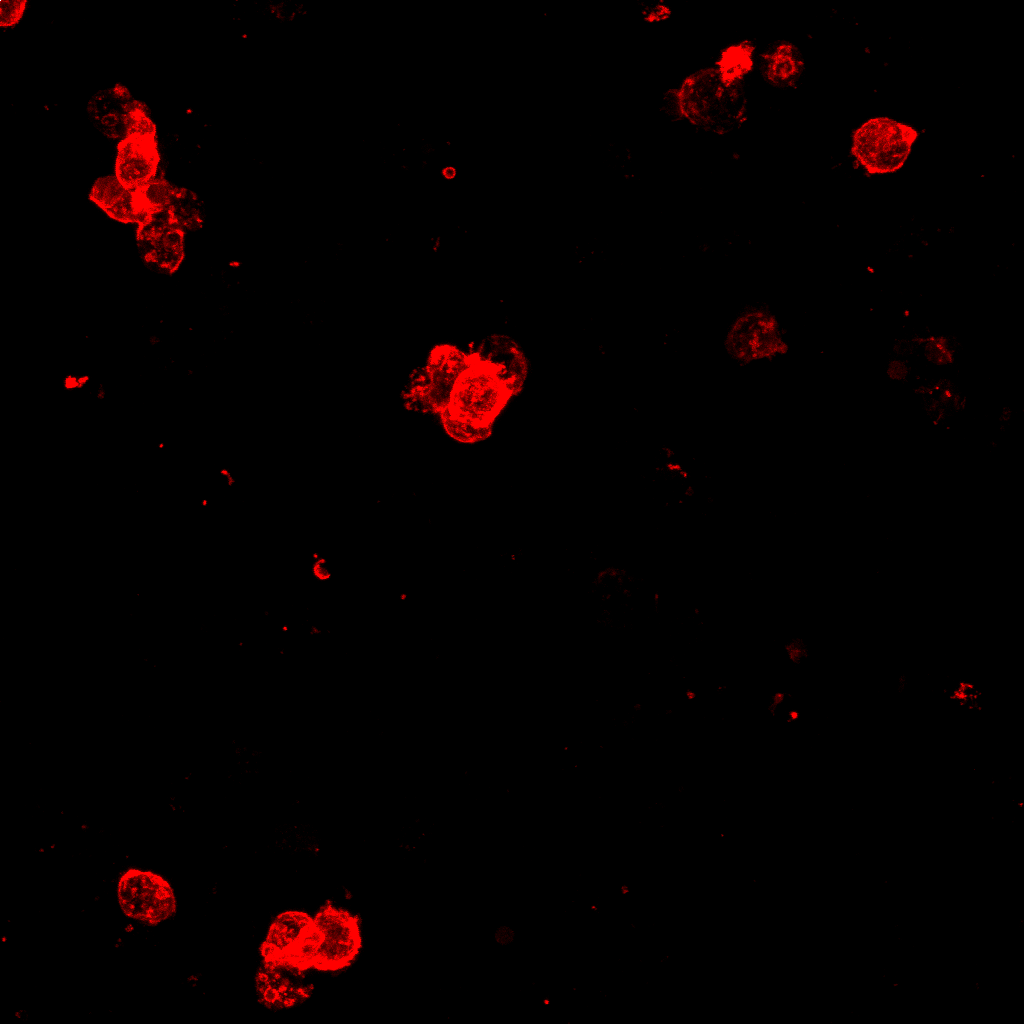

Supplement: Supplementary file 9 — Source data Fig. 5 [file 44319_2026_728_MOESM9_ESM.zip › Source_Data_Fig5/Fig5KL/MAX_20240718_pUASTattbNimAHA-NimAMYC-T2AmCD8GFP_S2_DAPI_A647MYC_FITCGFP_CY3HA_63X.lif - Series001_HA.tif]

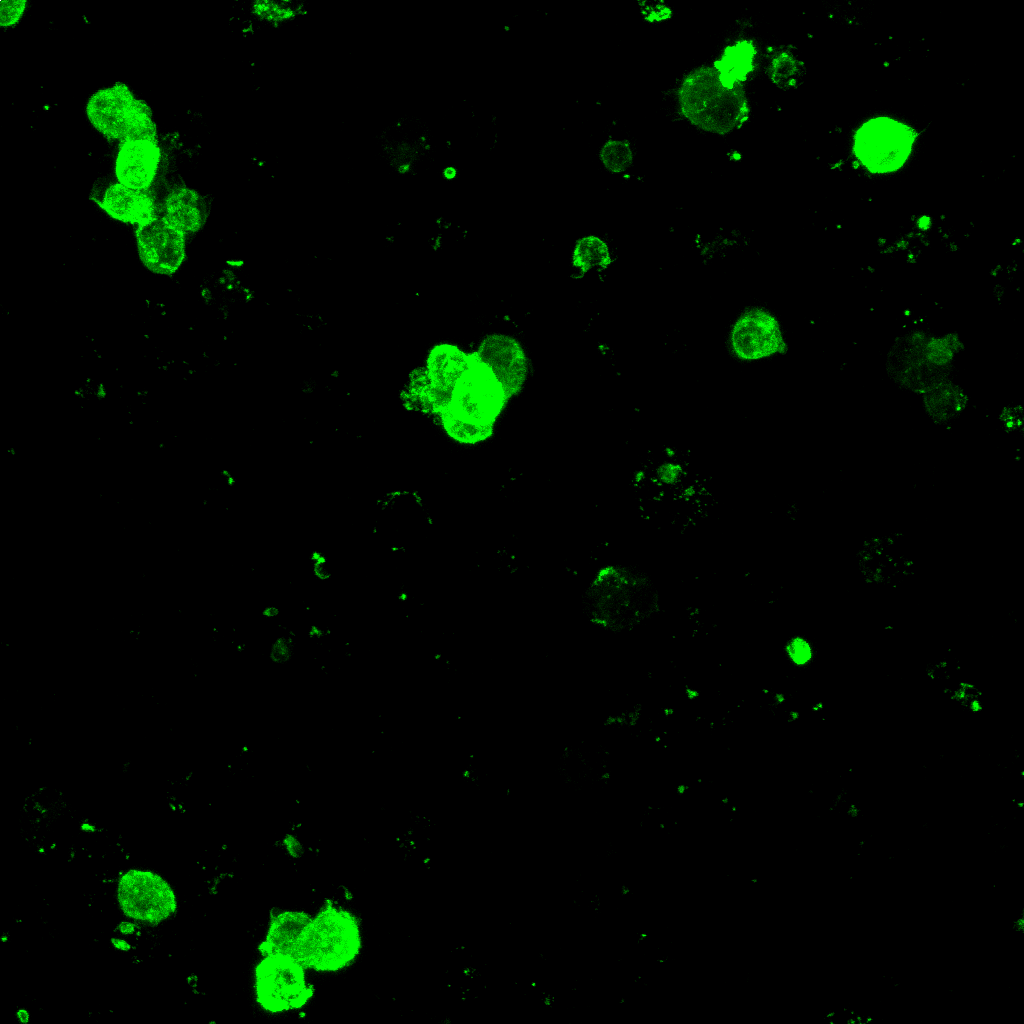

Supplement: Supplementary file 9 — Source data Fig. 5 [file 44319_2026_728_MOESM9_ESM.zip › Source_Data_Fig5/Fig5KL/MAX_20240718_pUASTattbNimAHA-NimAMYC-T2AmCD8GFP_S2_DAPI_A647MYC_FITCGFP_CY3HA_63X.lif - Series001_GFP.tif]

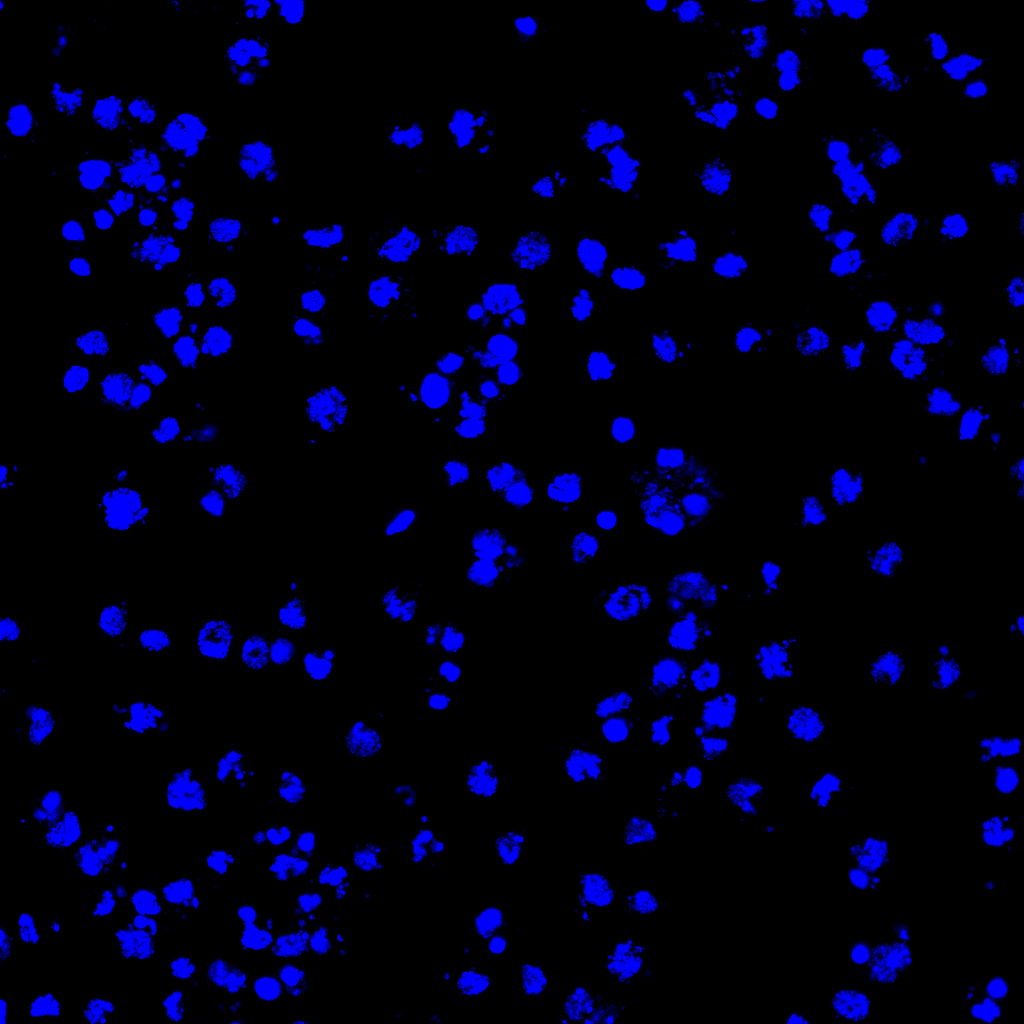

Supplement: Supplementary file 9 — Source data Fig. 5 [file 44319_2026_728_MOESM9_ESM.zip › Source_Data_Fig5/Fig5KL/MAX_20240718_pUASTattbNimAHA-NimAMYC-T2AmCD8GFP_S2_DAPI_A647MYC_FITCGFP_CY3HA_63X.lif - Series001_DAPI.tif]

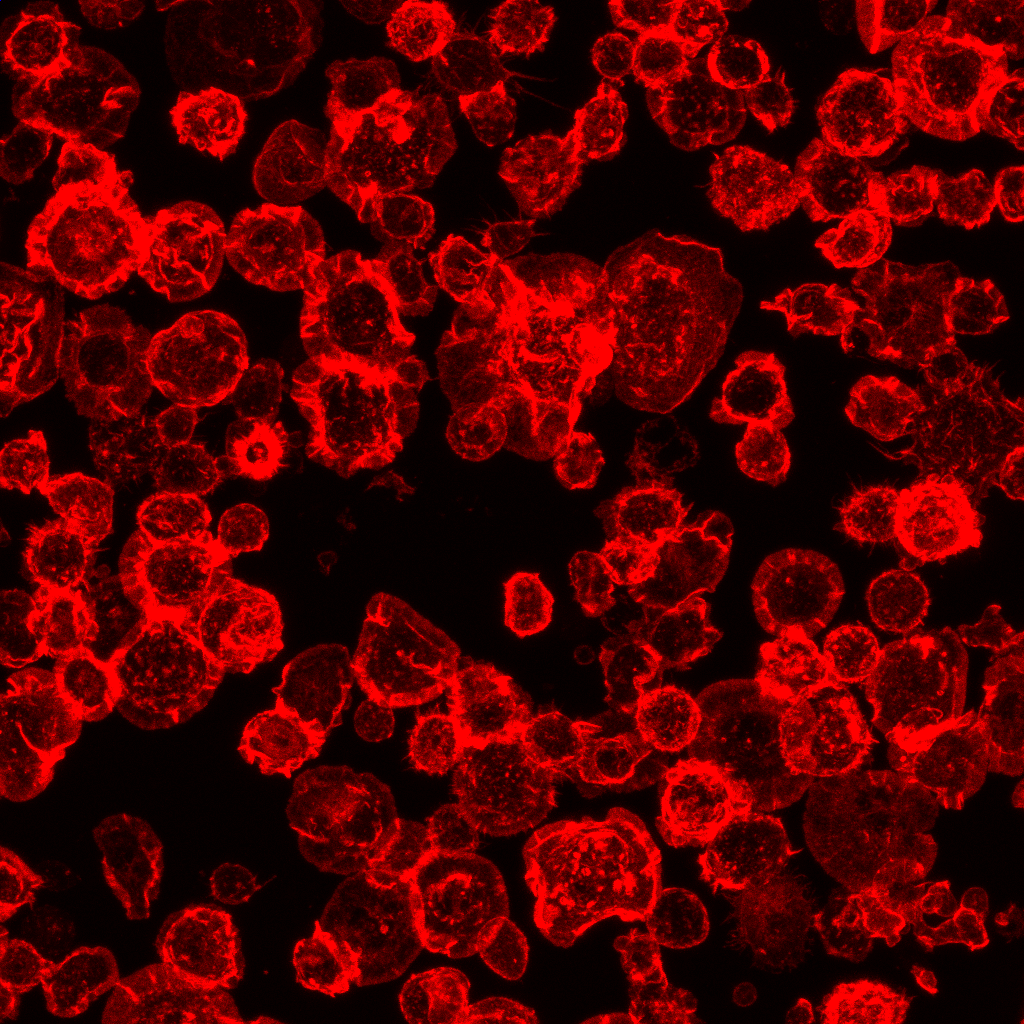

Supplement: Supplementary file 9 — Source data Fig. 5 [file 44319_2026_728_MOESM9_ESM.zip › Source_Data_Fig5/Fig5HI/MAX_20250214_pSKG4-pUASTattbT2AmCD8GFP_S2_DAPI_A647HA_FITCGFP_TRITCphall_63X.lif - Series002_Phalloidin.tif]

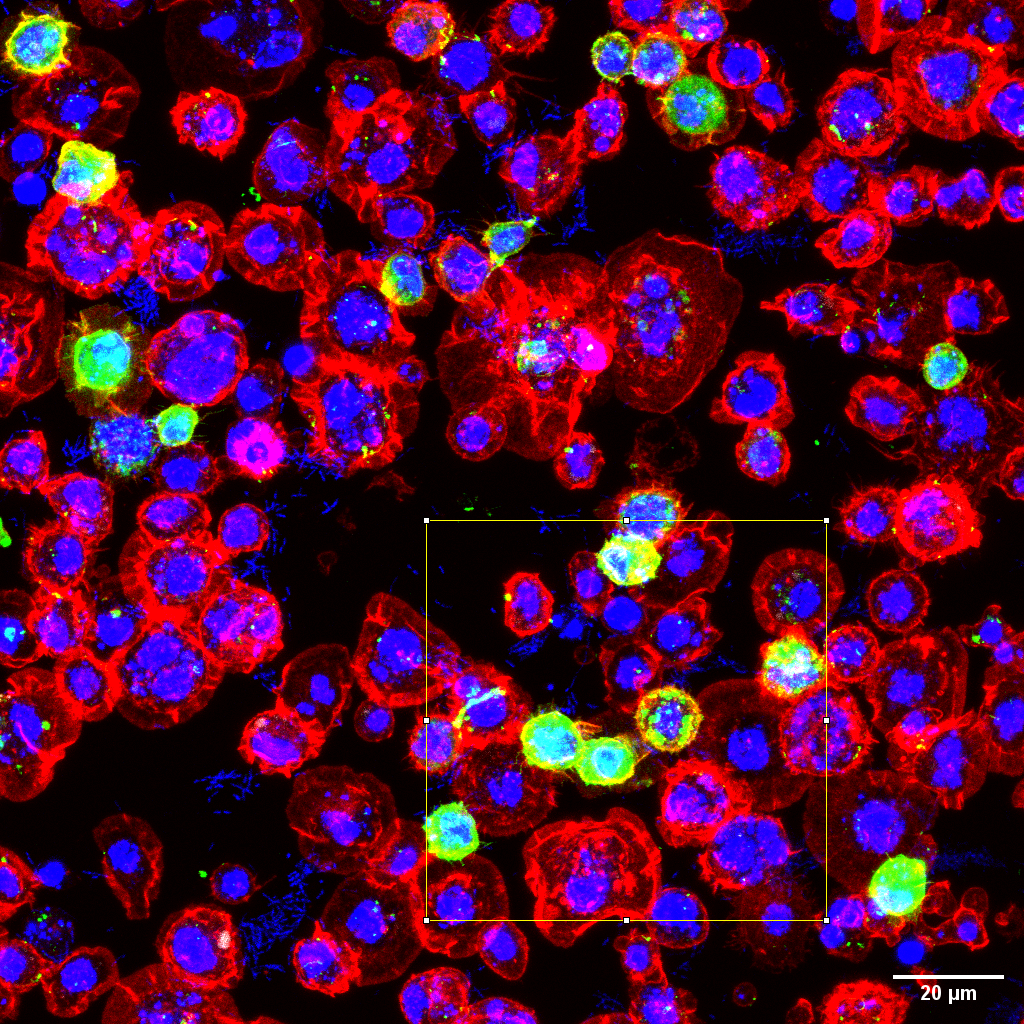

Supplement: Supplementary file 9 — Source data Fig. 5 [file 44319_2026_728_MOESM9_ESM.zip › Source_Data_Fig5/Fig5HI/MAX_20250214_pSKG4-pUASTattbT2AmCD8GFP_S2_DAPI_A647HA_FITCGFP_TRITCphall_63X.lif - Series002_Merge.tif]

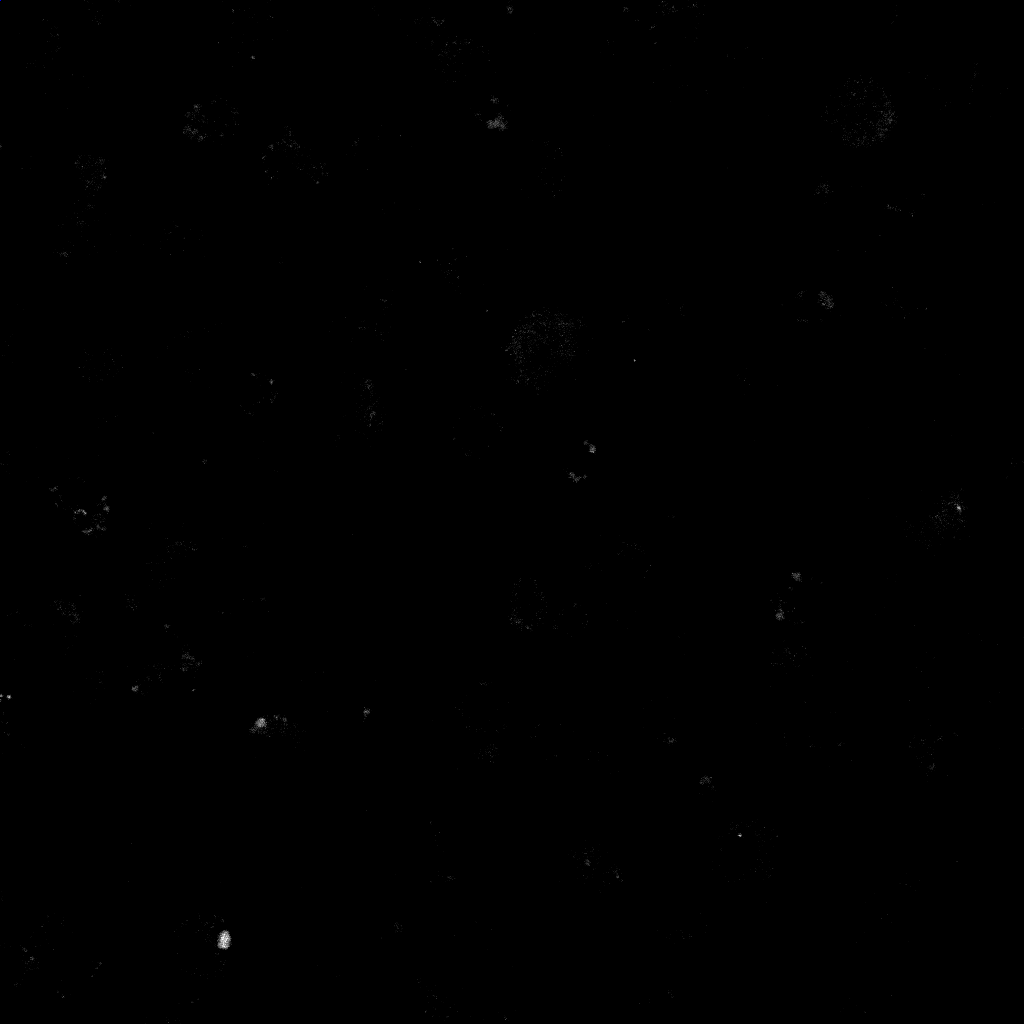

Supplement: Supplementary file 9 — Source data Fig. 5 [file 44319_2026_728_MOESM9_ESM.zip › Source_Data_Fig5/Fig5HI/MAX_20250214_pSKG4-pUASTattbT2AmCD8GFP_S2_DAPI_A647HA_FITCGFP_TRITCphall_63X.lif - Series002_HA.tif]

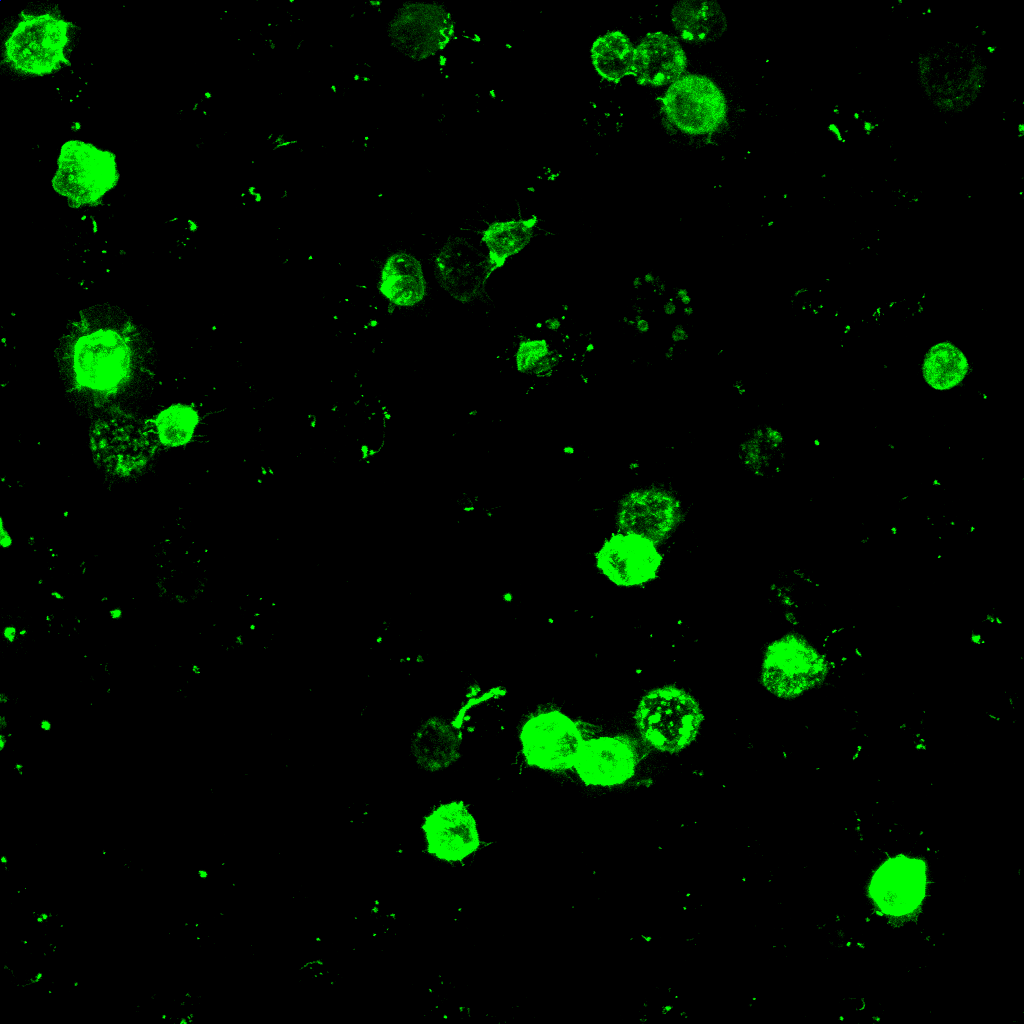

Supplement: Supplementary file 9 — Source data Fig. 5 [file 44319_2026_728_MOESM9_ESM.zip › Source_Data_Fig5/Fig5HI/MAX_20250214_pSKG4-pUASTattbT2AmCD8GFP_S2_DAPI_A647HA_FITCGFP_TRITCphall_63X.lif - Series002_GFP.tif]

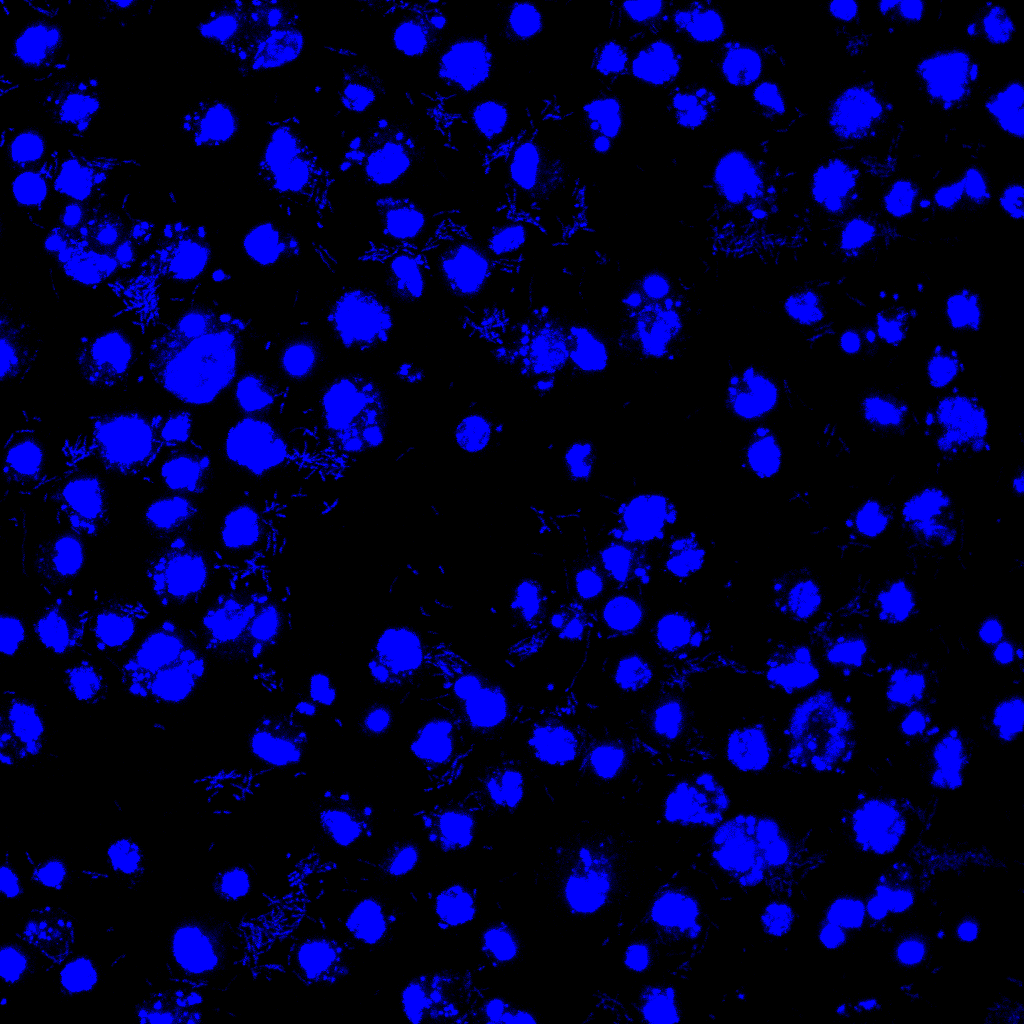

Supplement: Supplementary file 9 — Source data Fig. 5 [file 44319_2026_728_MOESM9_ESM.zip › Source_Data_Fig5/Fig5HI/MAX_20250214_pSKG4-pUASTattbT2AmCD8GFP_S2_DAPI_A647HA_FITCGFP_TRITCphall_63X.lif - Series002_DAPI.tif]

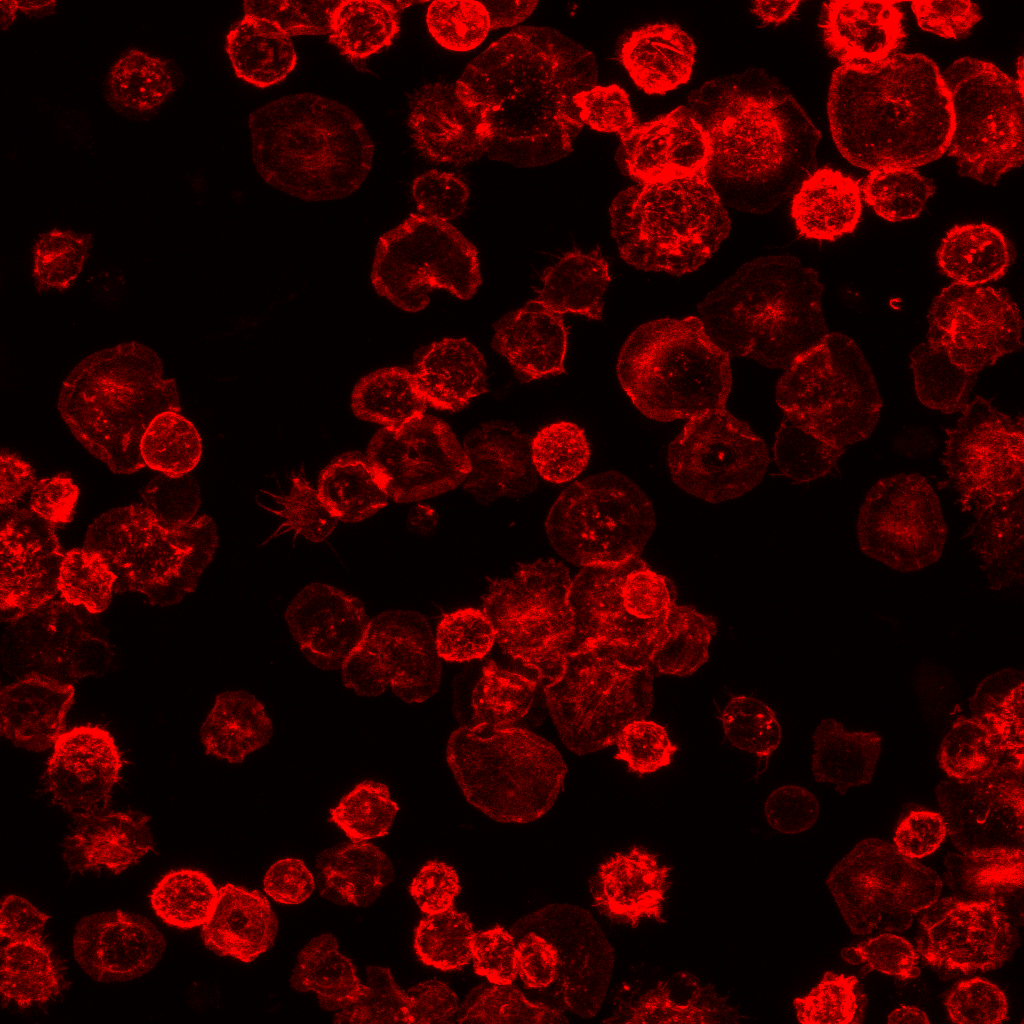

Supplement: Supplementary file 9 — Source data Fig. 5 [file 44319_2026_728_MOESM9_ESM.zip › Source_Data_Fig5/Fig5BCDE/MAX_20240712_pUASTattbT2A-mCD8GFP_S2_DAPI_Cy5mHA_FITCGFP_RodPhall_63X.lif - Series001_Phalloidin.tif]

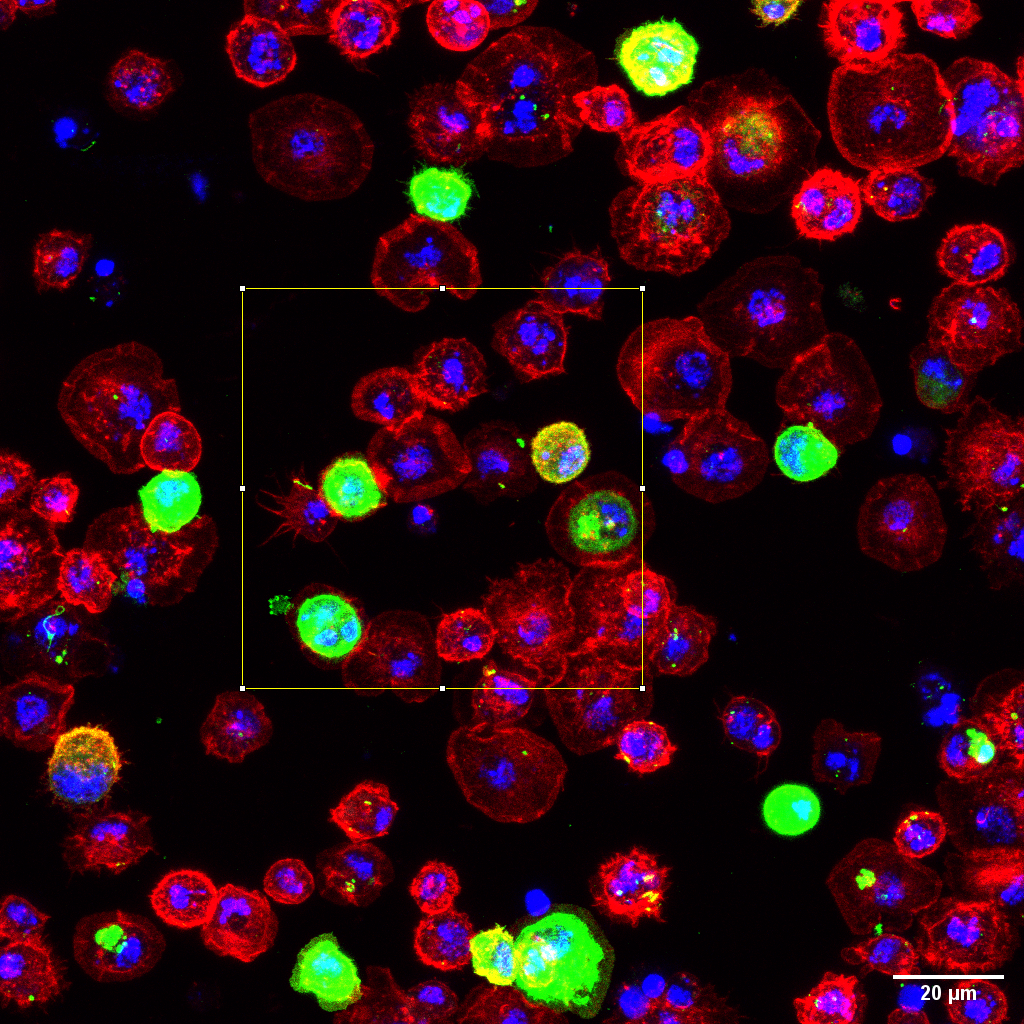

Supplement: Supplementary file 9 — Source data Fig. 5 [file 44319_2026_728_MOESM9_ESM.zip › Source_Data_Fig5/Fig5BCDE/MAX_20240712_pUASTattbT2A-mCD8GFP_S2_DAPI_Cy5mHA_FITCGFP_RodPhall_63X.lif - Series001_Merge.tif]

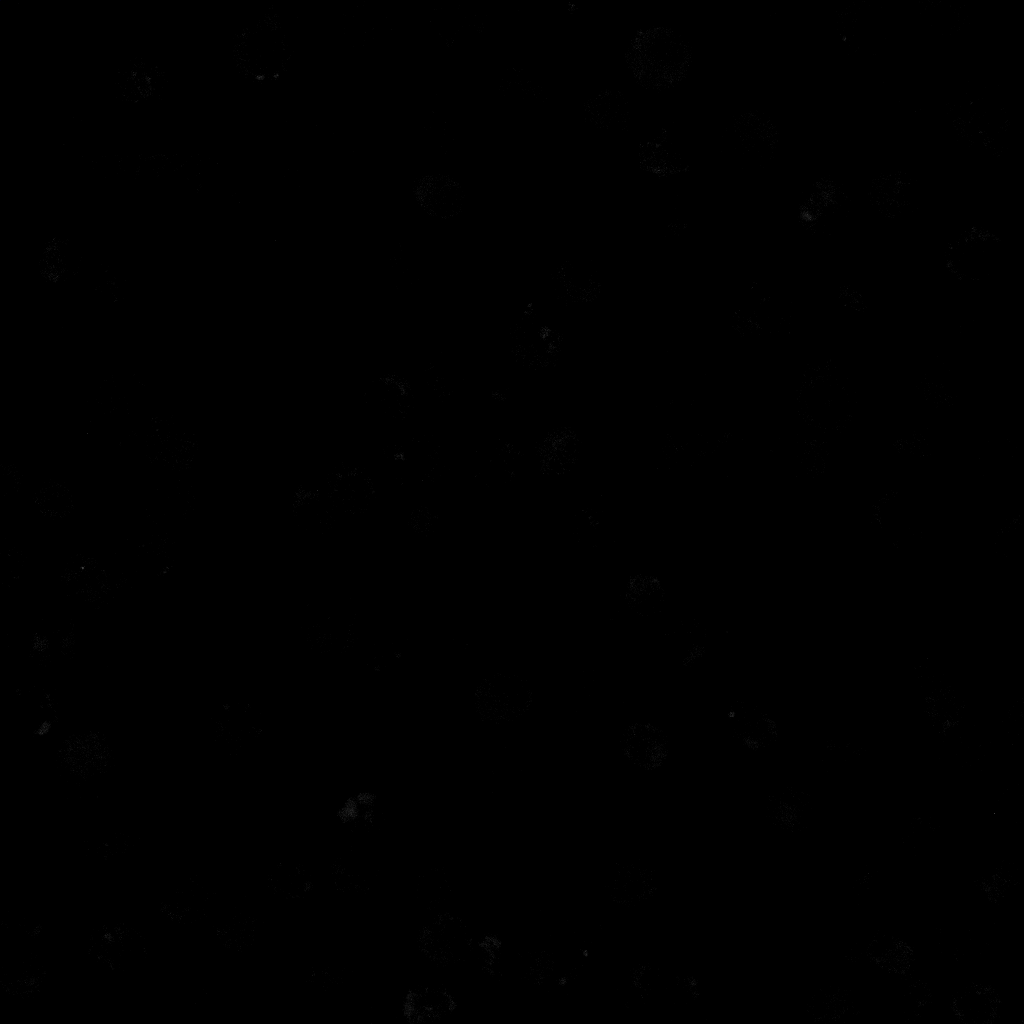

Supplement: Supplementary file 9 — Source data Fig. 5 [file 44319_2026_728_MOESM9_ESM.zip › Source_Data_Fig5/Fig5BCDE/MAX_20240712_pUASTattbT2A-mCD8GFP_S2_DAPI_Cy5mHA_FITCGFP_RodPhall_63X.lif - Series001_HA.tif]

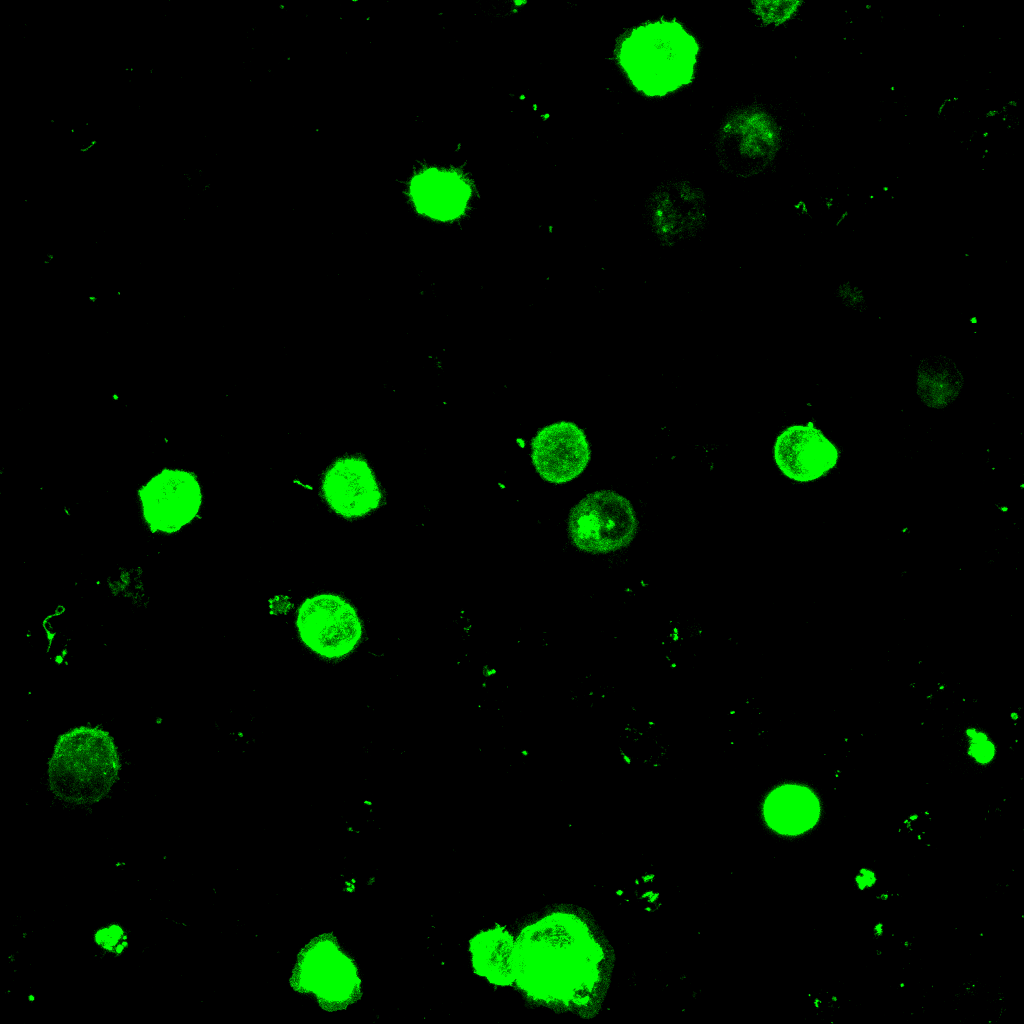

Supplement: Supplementary file 9 — Source data Fig. 5 [file 44319_2026_728_MOESM9_ESM.zip › Source_Data_Fig5/Fig5BCDE/MAX_20240712_pUASTattbT2A-mCD8GFP_S2_DAPI_Cy5mHA_FITCGFP_RodPhall_63X.lif - Series001_GFP.tif]

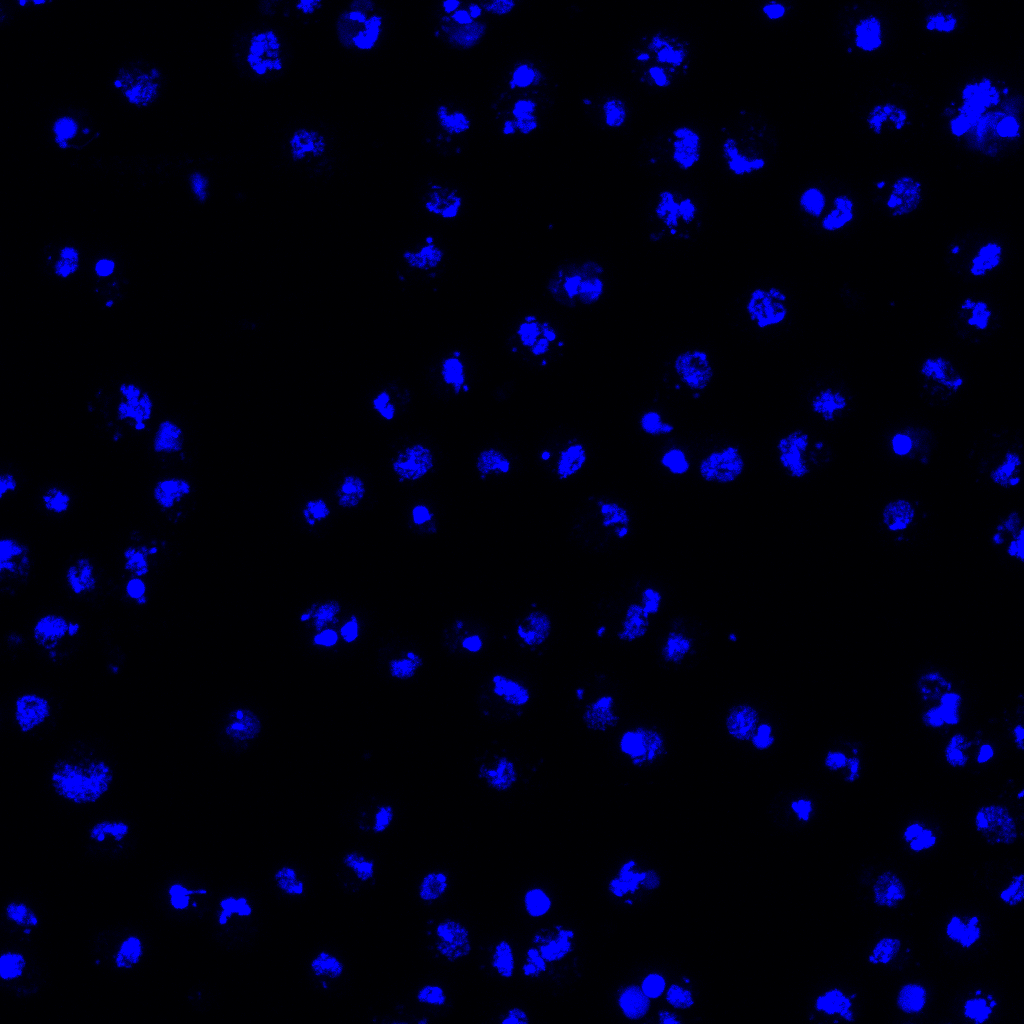

Supplement: Supplementary file 9 — Source data Fig. 5 [file 44319_2026_728_MOESM9_ESM.zip › Source_Data_Fig5/Fig5BCDE/MAX_20240712_pUASTattbT2A-mCD8GFP_S2_DAPI_Cy5mHA_FITCGFP_RodPhall_63X.lif - Series001_DAPI.tif]

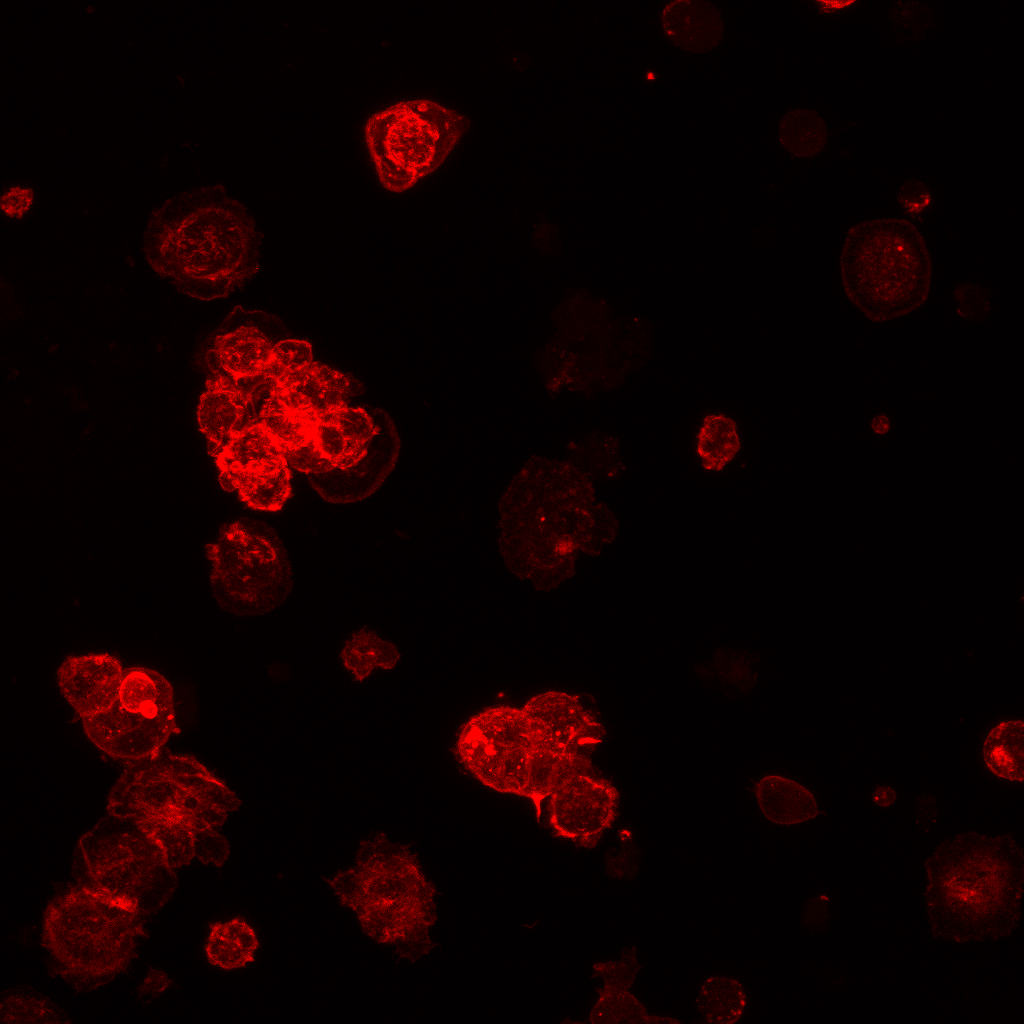

Supplement: Supplementary file 9 — Source data Fig. 5 [file 44319_2026_728_MOESM9_ESM.zip › Source_Data_Fig5/Fig5BCDE/MAX_20240712_pUASTattbNimAHA-T2A-mCD8GFP_S2_DAPI_Cy5mHA_FITCGFP_RodPhall_63X.lif - Series003_Phalloidin.tif]

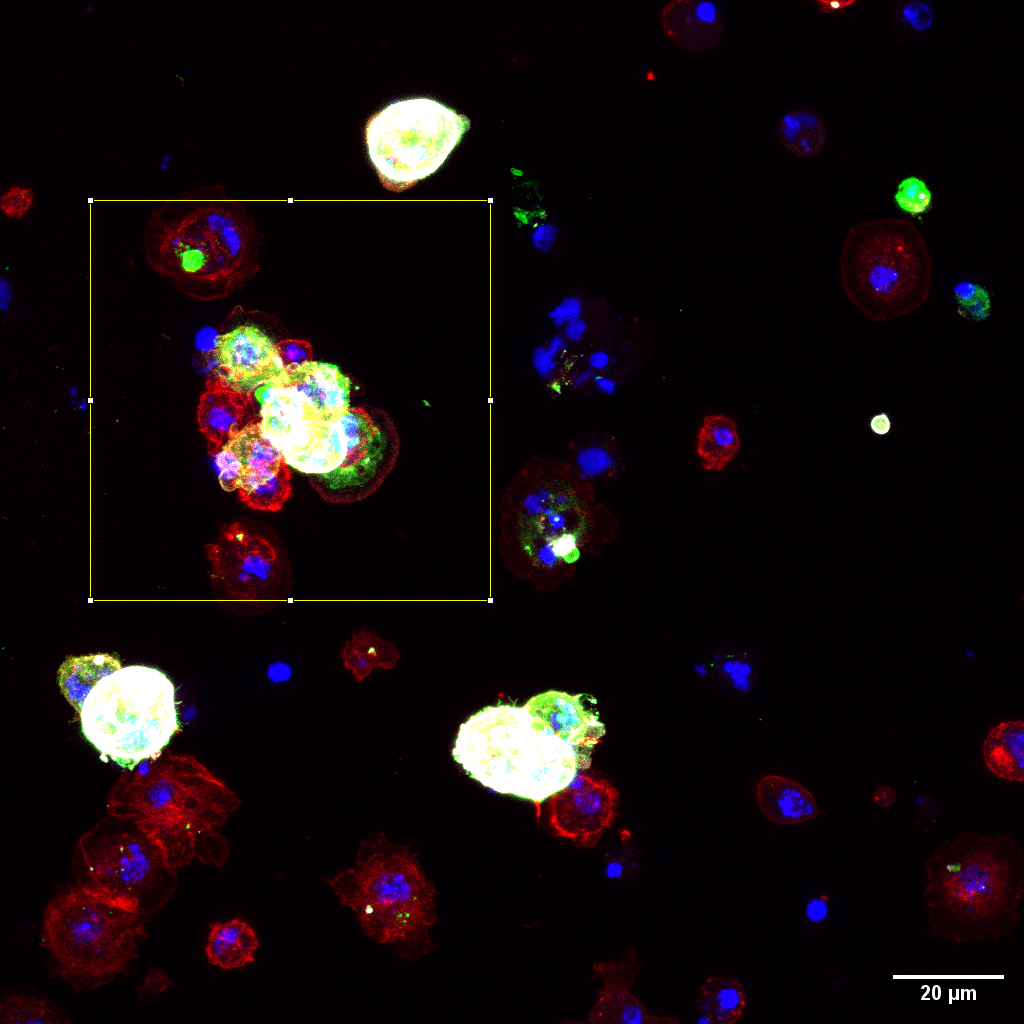

Supplement: Supplementary file 9 — Source data Fig. 5 [file 44319_2026_728_MOESM9_ESM.zip › Source_Data_Fig5/Fig5BCDE/MAX_20240712_pUASTattbNimAHA-T2A-mCD8GFP_S2_DAPI_Cy5mHA_FITCGFP_RodPhall_63X.lif - Series003_Merge.tif]

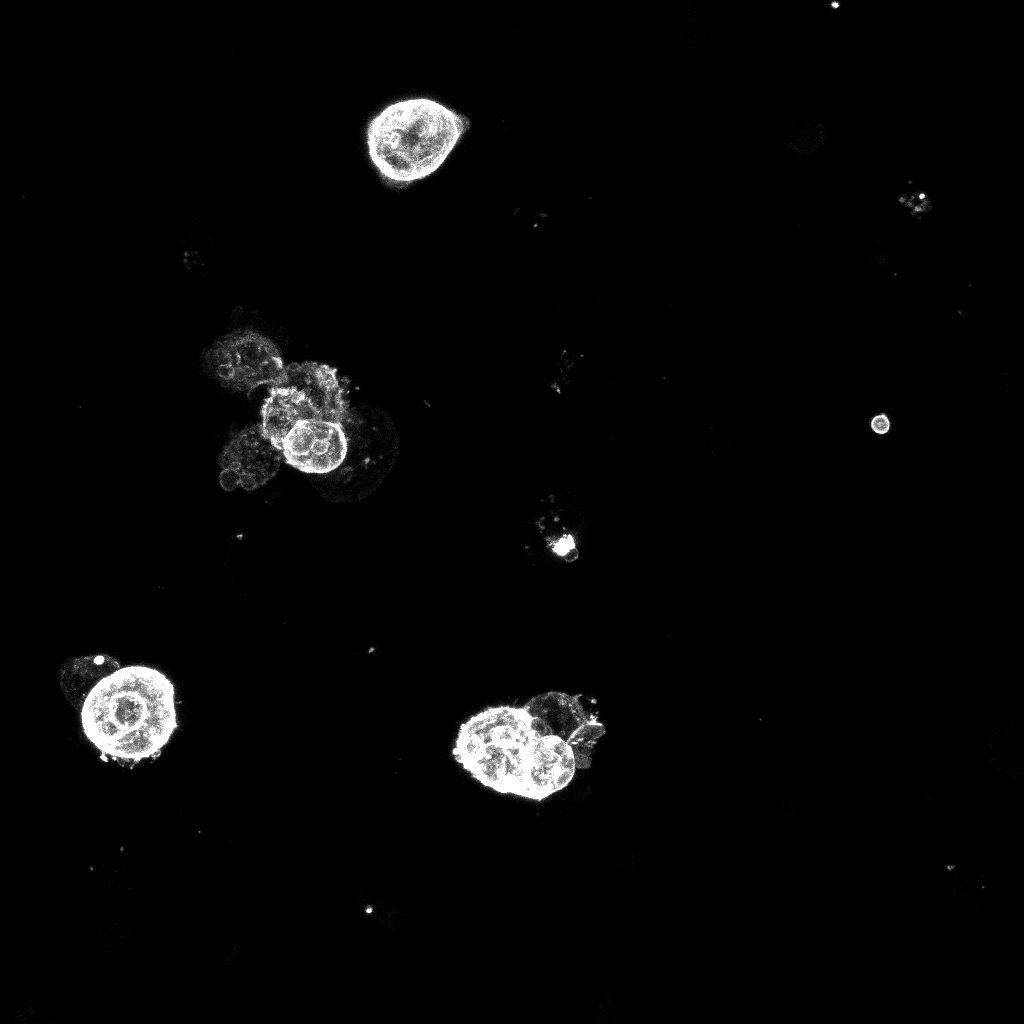

Supplement: Supplementary file 9 — Source data Fig. 5 [file 44319_2026_728_MOESM9_ESM.zip › Source_Data_Fig5/Fig5BCDE/MAX_20240712_pUASTattbNimAHA-T2A-mCD8GFP_S2_DAPI_Cy5mHA_FITCGFP_RodPhall_63X.lif - Series003_HA.tif]

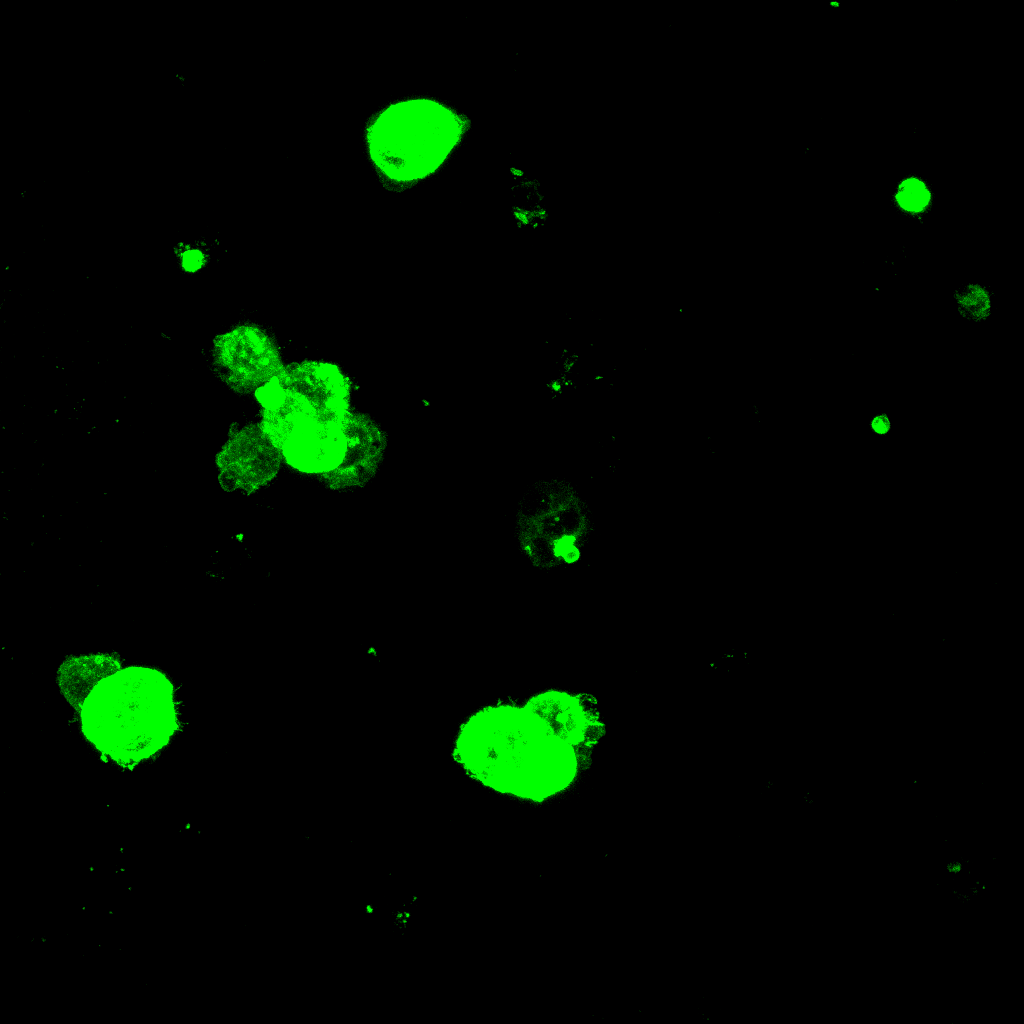

Supplement: Supplementary file 9 — Source data Fig. 5 [file 44319_2026_728_MOESM9_ESM.zip › Source_Data_Fig5/Fig5BCDE/MAX_20240712_pUASTattbNimAHA-T2A-mCD8GFP_S2_DAPI_Cy5mHA_FITCGFP_RodPhall_63X.lif - Series003_GFP.tif]

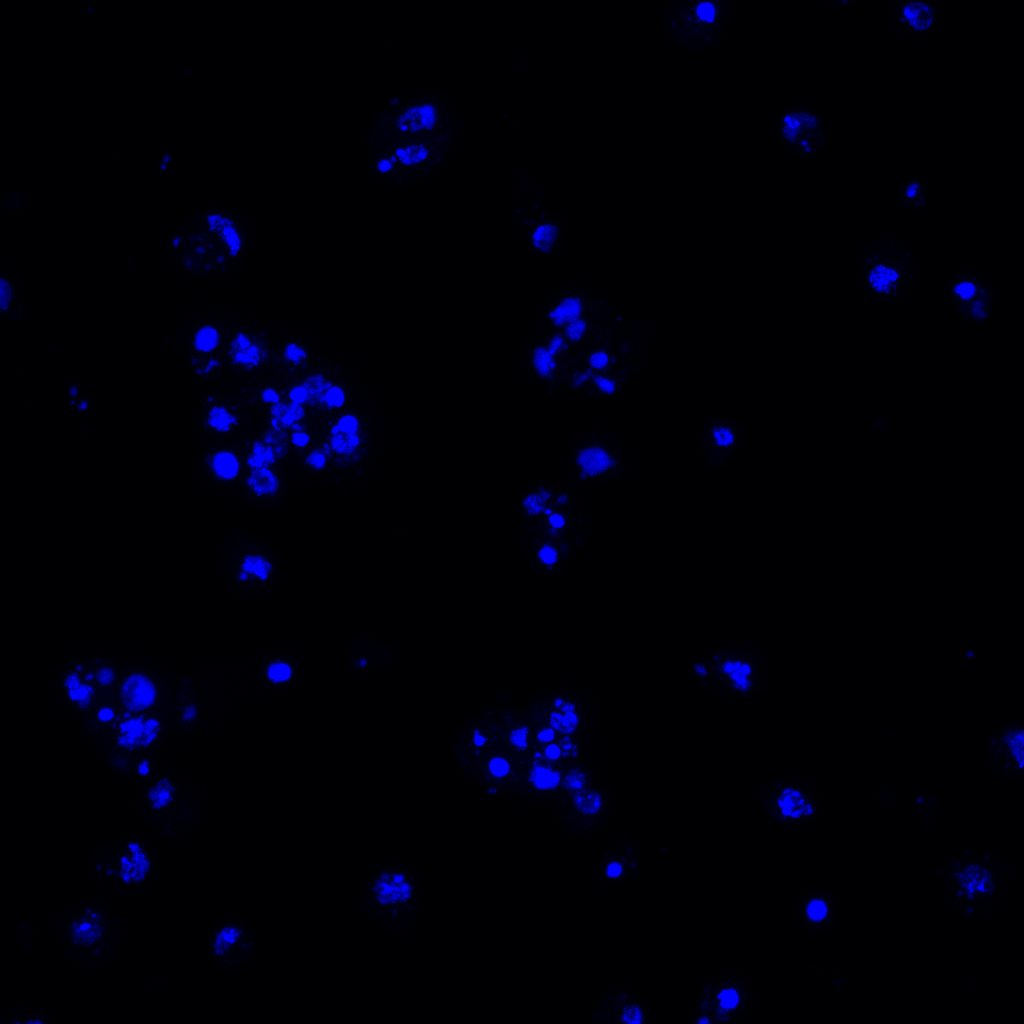

Supplement: Supplementary file 9 — Source data Fig. 5 [file 44319_2026_728_MOESM9_ESM.zip › Source_Data_Fig5/Fig5BCDE/MAX_20240712_pUASTattbNimAHA-T2A-mCD8GFP_S2_DAPI_Cy5mHA_FITCGFP_RodPhall_63X.lif - Series003_DAPI.tif]
